# Supplementary material for: Decade-Long Sustained Cellular Immunity Induced by Sequential and Repeated Vaccination with Four Heterologous HIV Vaccines in Rhesus Macaques
Source: Vaccines (Basel). 2025 Mar 21;13(4):338. doi: 10.3390/vaccines13040338 (PMC12031043; doi:10.3390/vaccines13040338)
Supplement: Supplementary file 1 [file vaccines-13-00338-s001.zip › Supplementary Materials File S1_Gating strategy.pdf]

Gating strategy：

A standardized FlowJo (Tree Star Inc., Ashland, OR) analysis template was constructed. A synchronized group feature was used and all gate adjustments were identical for the mock result and all antigen results within a given sample. This template included a gate to define the lymphocyte population on a forward scatter versus side scatter plot and a CD3 with a gate on the $CD3^+$ population. A CD4 and CD8 dot plot (gated on $CD3^+$ lymphocytes) was displayed with gates drawn $CD3^+CD4^+$ and $CD3^+$ $CD8^+$ double-positive populations. For each T-cell subset, $CD3^+CD4^+$ and $CD3^+$ $CD8^+$, three dot plots were displayed as side scatter versus IFN-γ, IL-2, or IFN-γ + IL-2, A gate on each of these graphs was set to include positive cells (see Figure 5). These results were exported from FlowJo into a spreadsheet and normalized to events per 0.1 million lymphocytes for the reported final results. These normalized values are hereafter referred to as the responses. The data analysis program FACSDiva (Version 6.0A) was used to generate the graphical representation in Figure. 2.
