# Supplementary material for: Decade-Long Sustained Cellular Immunity Induced by Sequential and Repeated Vaccination with Four Heterologous HIV Vaccines in Rhesus Macaques
Source: Vaccines (Basel). 2025 Mar 21;13(4):338. doi: 10.3390/vaccines13040338 (PMC12031043; doi:10.3390/vaccines13040338)
Supplement: Supplementary file 1 [file vaccines-13-00338-s001.zip › Supplementary Materials File S2_Figures for ICS.pdf]

negative control

FACSDiva Version 6.0

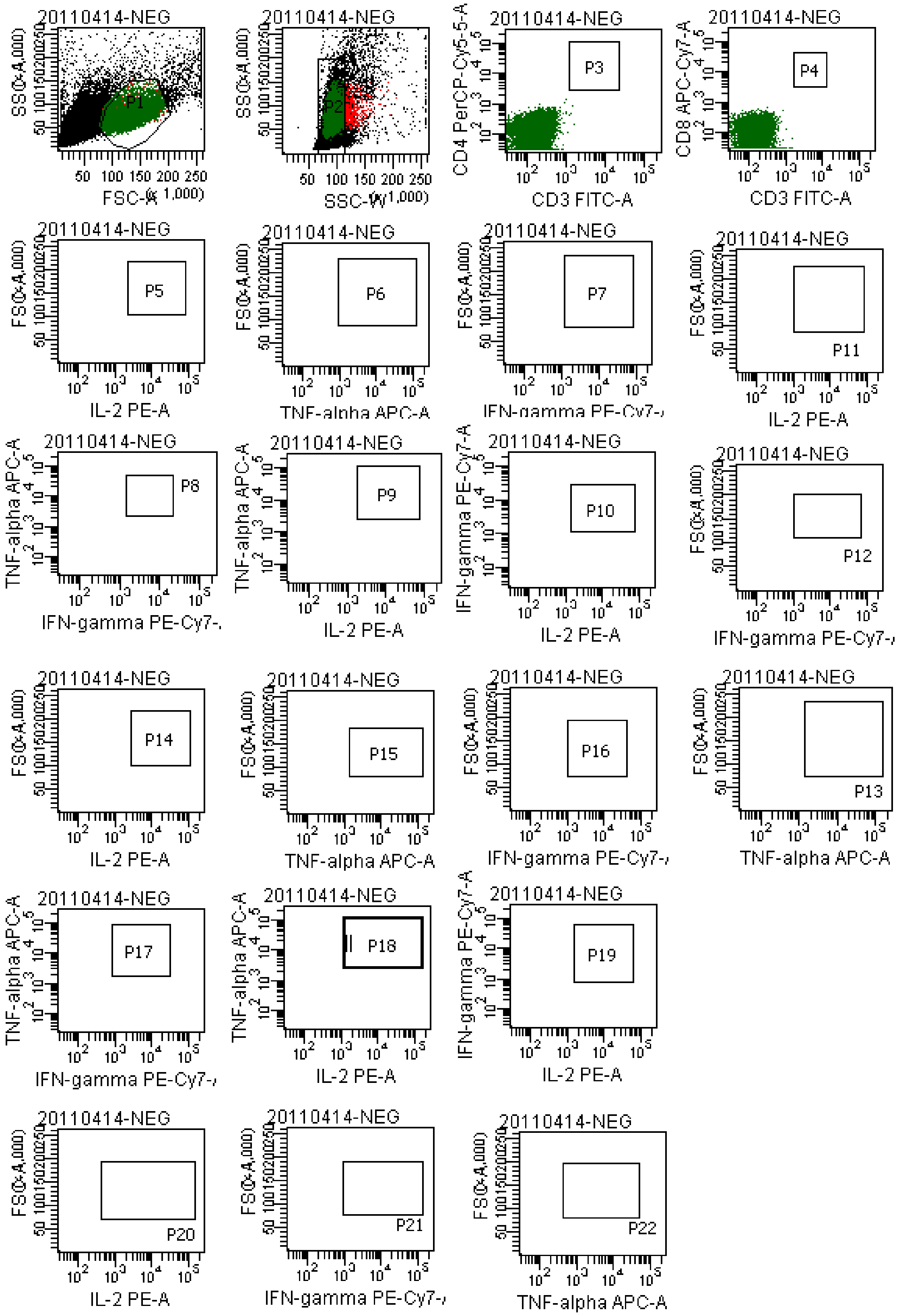

negative control

FACSDiva Version 6.0

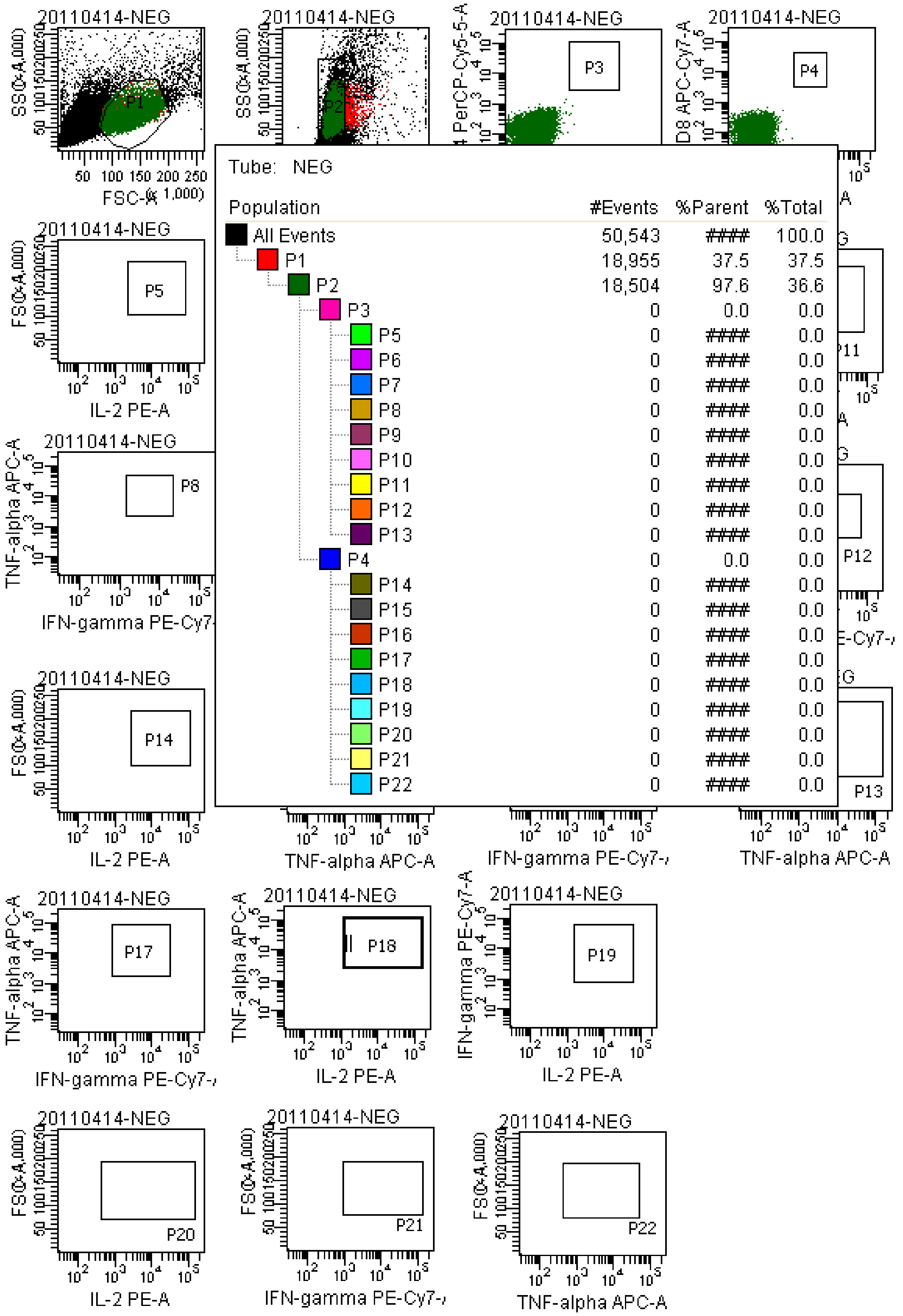

Tube: NEG

Population	#Events	%Parent	%Total
All Events	50,543	####	100.0
P1	18,955	37.5	37.5
P2	18,504	97.6	36.6
P3	0	0.0	0.0
P5	0	####	0.0
P6	0	####	0.0
P7	0	####	0.0
P8	0	####	0.0
P9	0	####	0.0
P10	0	####	0.0
P11	0	####	0.0
P12	0	####	0.0
P13	0	####	0.0
P4	0	0.0	0.0
P14	0	####	0.0
P15	0	####	0.0
P16	0	####	0.0
P17	0	####	0.0
P18	0	####	0.0
P19	0	####	0.0
P20	0	####	0.0
P21	0	####	0.0
P22	0	####	0.0

positive control

FACSDiva Version 6.0

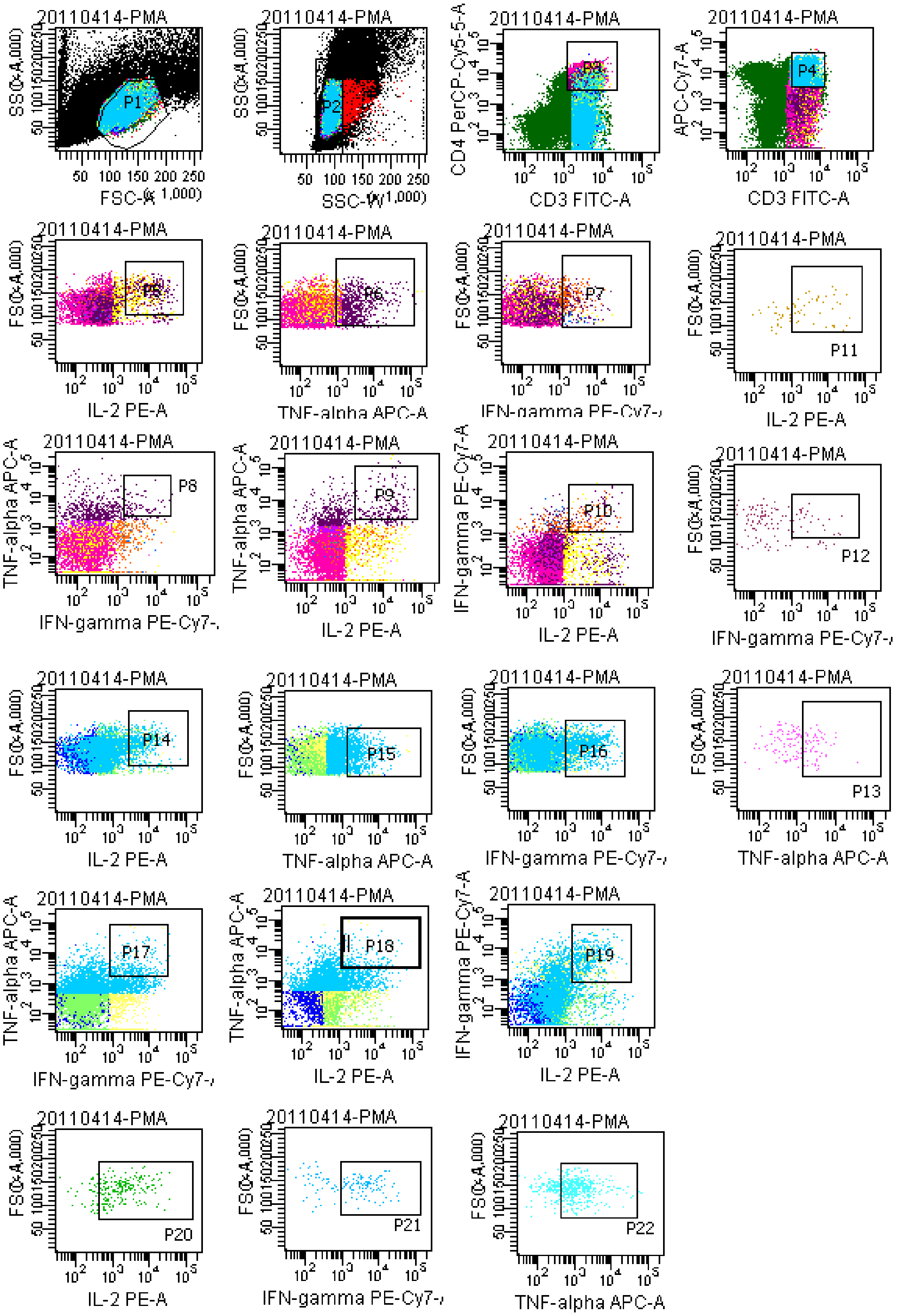

positive control

FACSDiva Version 6.0

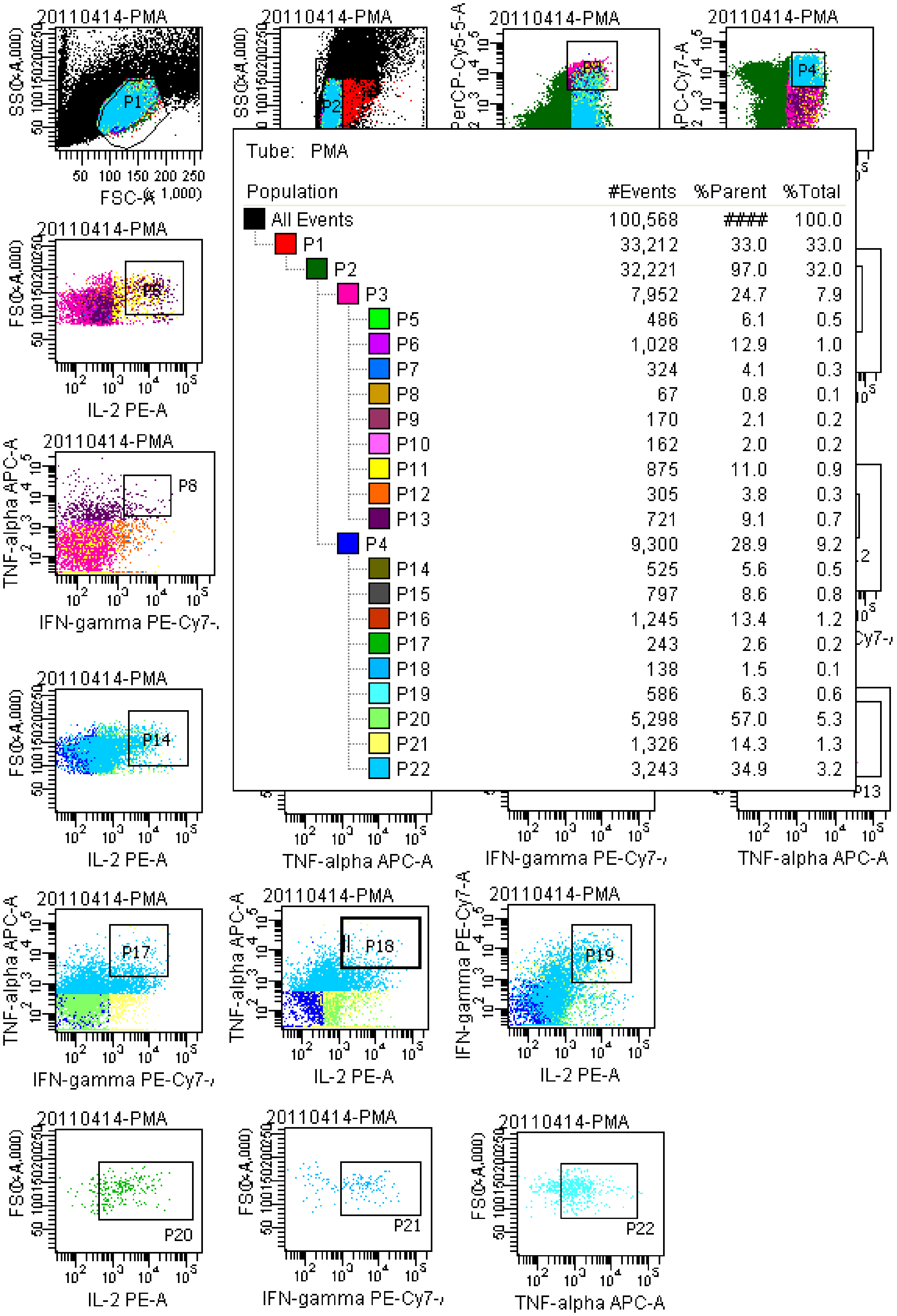

Tube: PMA

Population	#Events	%Parent	%Total
All Events	100,568	####	100.0
P1	33,212	33.0	33.0
P2	32,221	97.0	32.0
P3	7,952	24.7	7.9
P5	486	6.1	0.5
P6	1,028	12.9	1.0
P7	324	4.1	0.3
P8	67	0.8	0.1
P9	170	2.1	0.2
P10	162	2.0	0.2
P11	875	11.0	0.9
P12	305	3.8	0.3
P13	721	9.1	0.7
P4	9,300	28.9	9.2
P14	525	5.6	0.5
P15	797	8.6	0.8
P16	1,245	13.4	1.2
P17	243	2.6	0.2
P18	138	1.5	0.1
P19	586	6.3	0.6
P20	5,298	57.0	5.3
P21	1,326	14.3	1.3
P22	3,243	34.9	3.2

248W-4vector:98R0053 (peptide)

FACSDiva Version 6.0

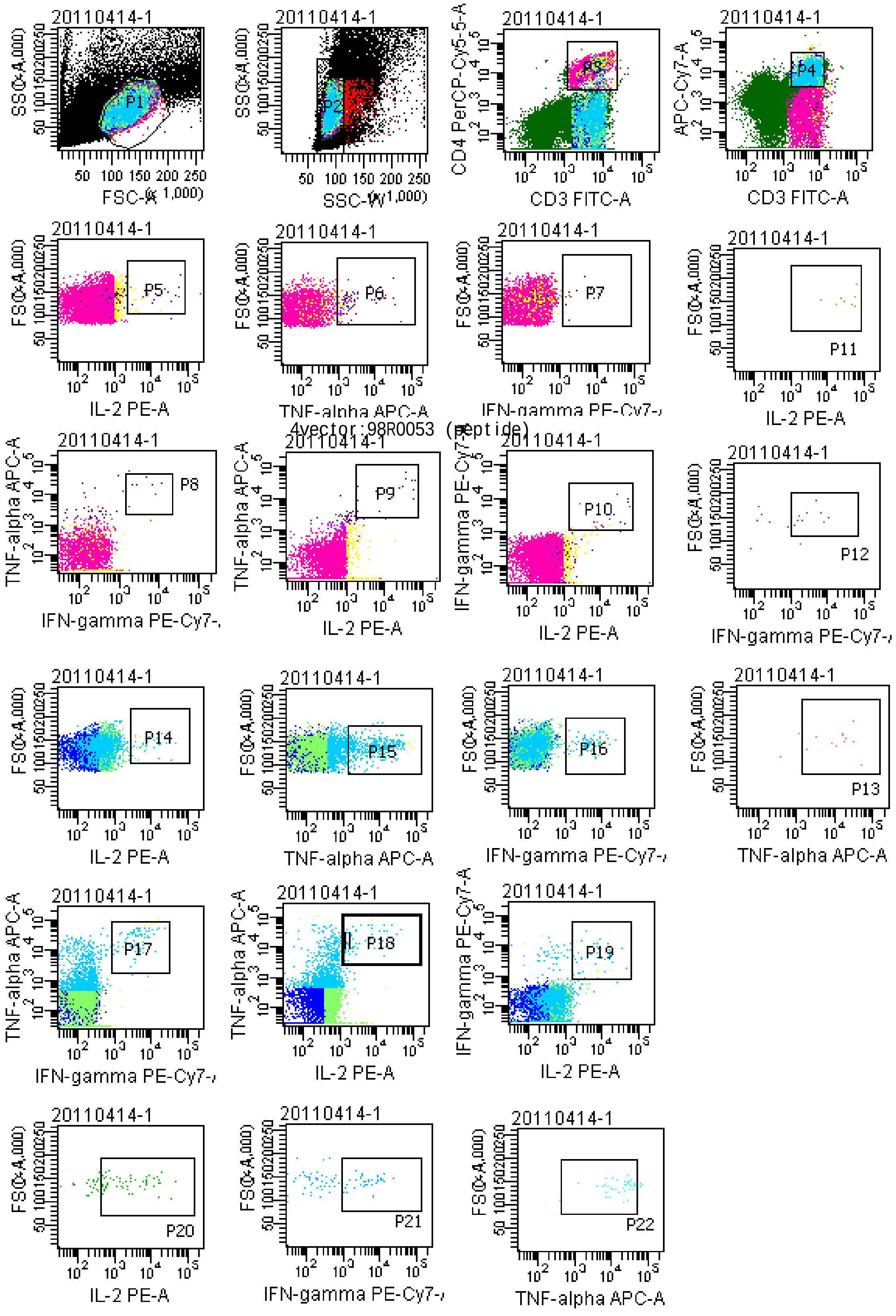

248W-4vector:98R0053 (peptide)

FACSDiva Version 6.0

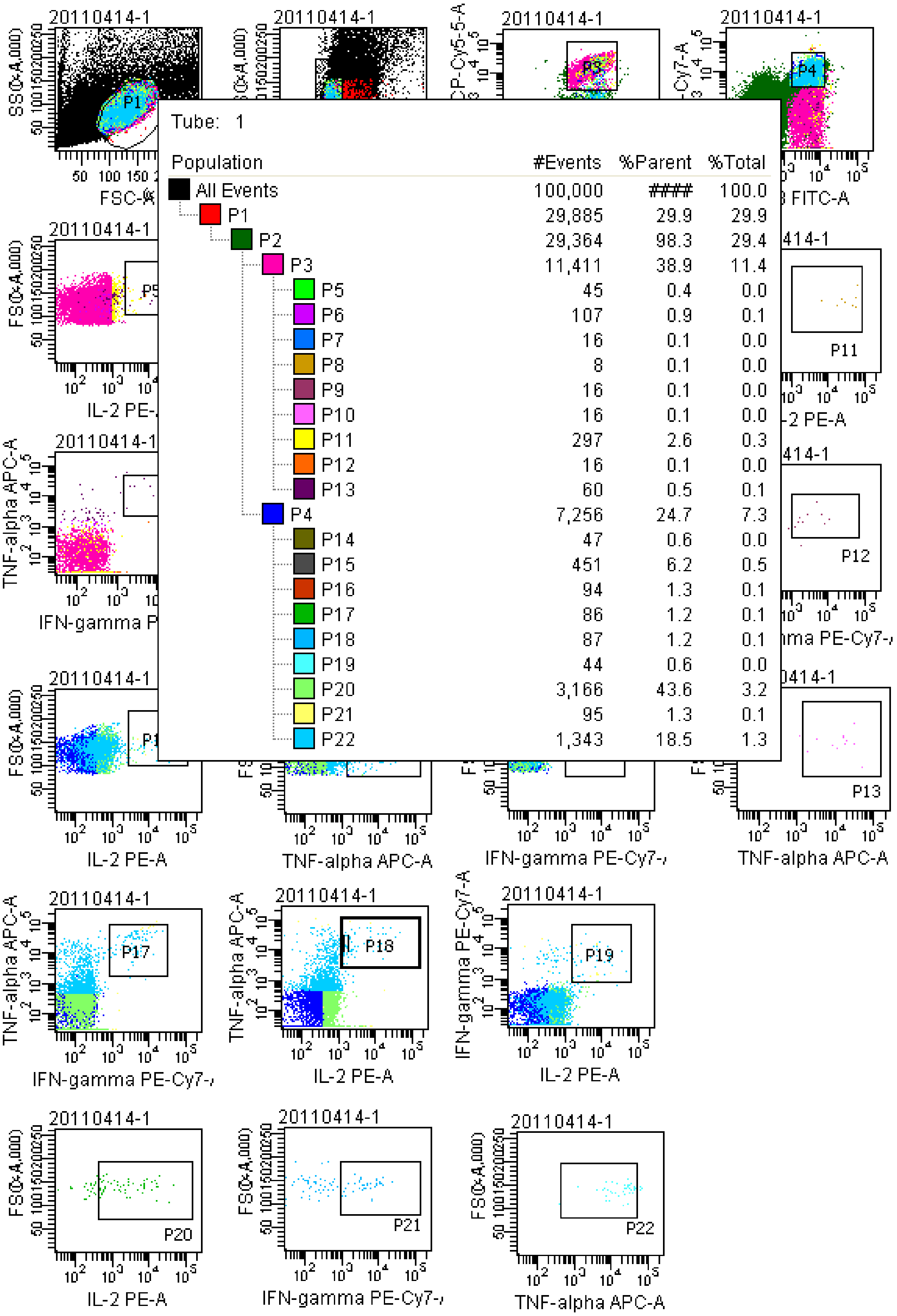

Tube: 1

Population	#Events	%Parent	%Total
All Events	100,000	####	100.0
P1	29,885	29.9	29.9
P2	29,364	98.3	29.4
P3	11,411	38.9	11.4
P5	45	0.4	0.0
P6	107	0.9	0.1
P7	16	0.1	0.0
P8	8	0.1	0.0
P9	16	0.1	0.0
P10	16	0.1	0.0
P11	297	2.6	0.3
P12	16	0.1	0.0
P13	60	0.5	0.1
P4	7,256	24.7	7.3
P14	47	0.6	0.0
P15	451	6.2	0.5
P16	94	1.3	0.1
P17	86	1.2	0.1
P18	87	1.2	0.1
P19	44	0.6	0.0
P20	3,166	43.6	3.2
P21	95	1.3	0.1
P22	1,343	18.5	1.3

FACSDiva Version 6.0

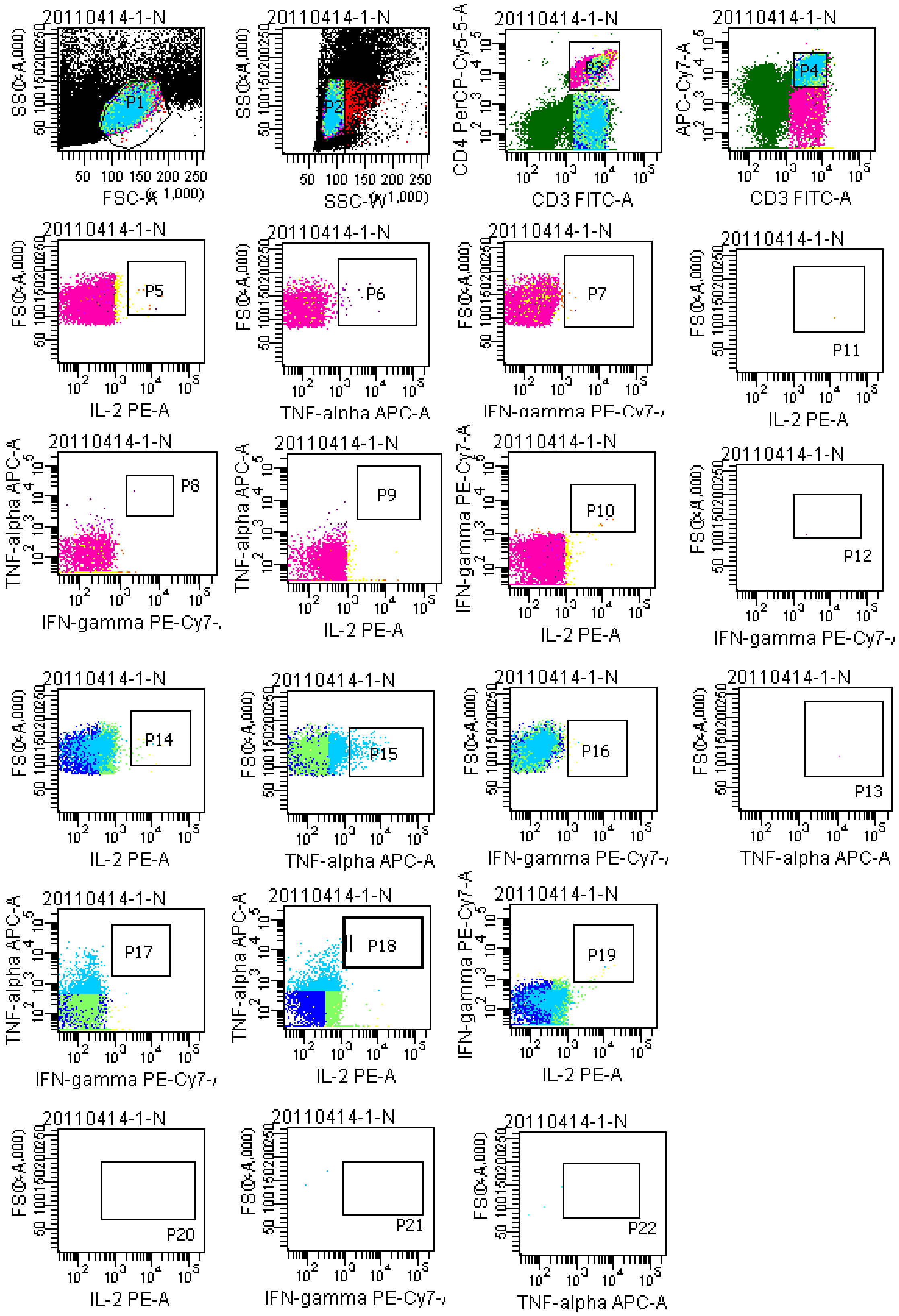

FACSDiva Version 6.0

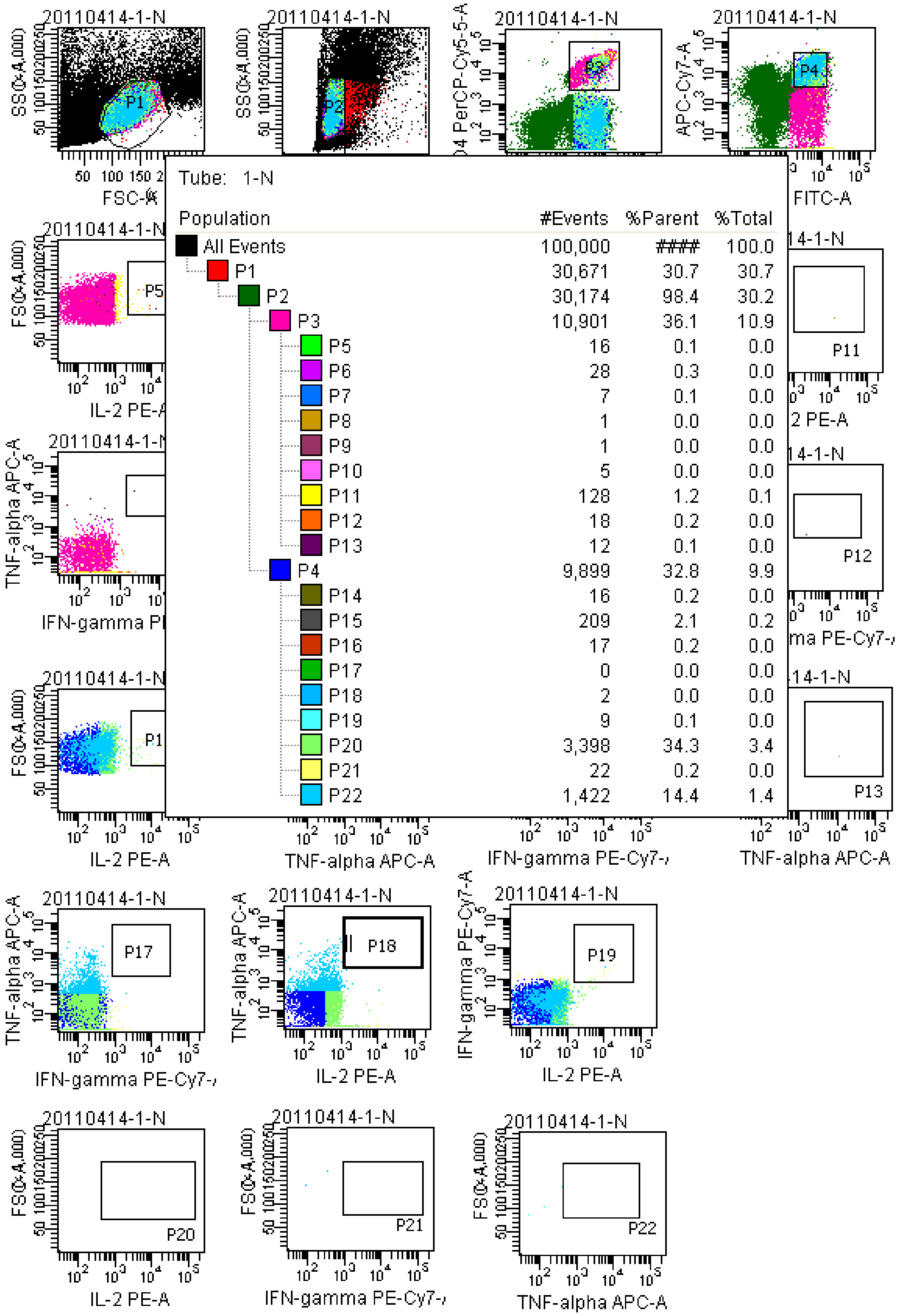

Tube: 1-N

Population	#Events	%Parent	%Total
All Events	100,000	####	100.0
P1	30,671	30.7	30.7
P2	30,174	98.4	30.2
P3	10,901	36.1	10.9
P5	16	0.1	0.0
P6	28	0.3	0.0
P7	7	0.1	0.0
P8	1	0.0	0.0
P9	1	0.0	0.0
P10	5	0.0	0.0
P11	128	1.2	0.1
P12	18	0.2	0.0
P13	12	0.1	0.0
P4	9,899	32.8	9.9
P14	16	0.2	0.0
P15	209	2.1	0.2
P16	17	0.2	0.0
P17	0	0.0	0.0
P18	2	0.0	0.0
P19	9	0.1	0.0
P20	3,398	34.3	3.4
P21	22	0.2	0.0
P22	1,422	14.4	1.4

FACSDiva Version 6.0

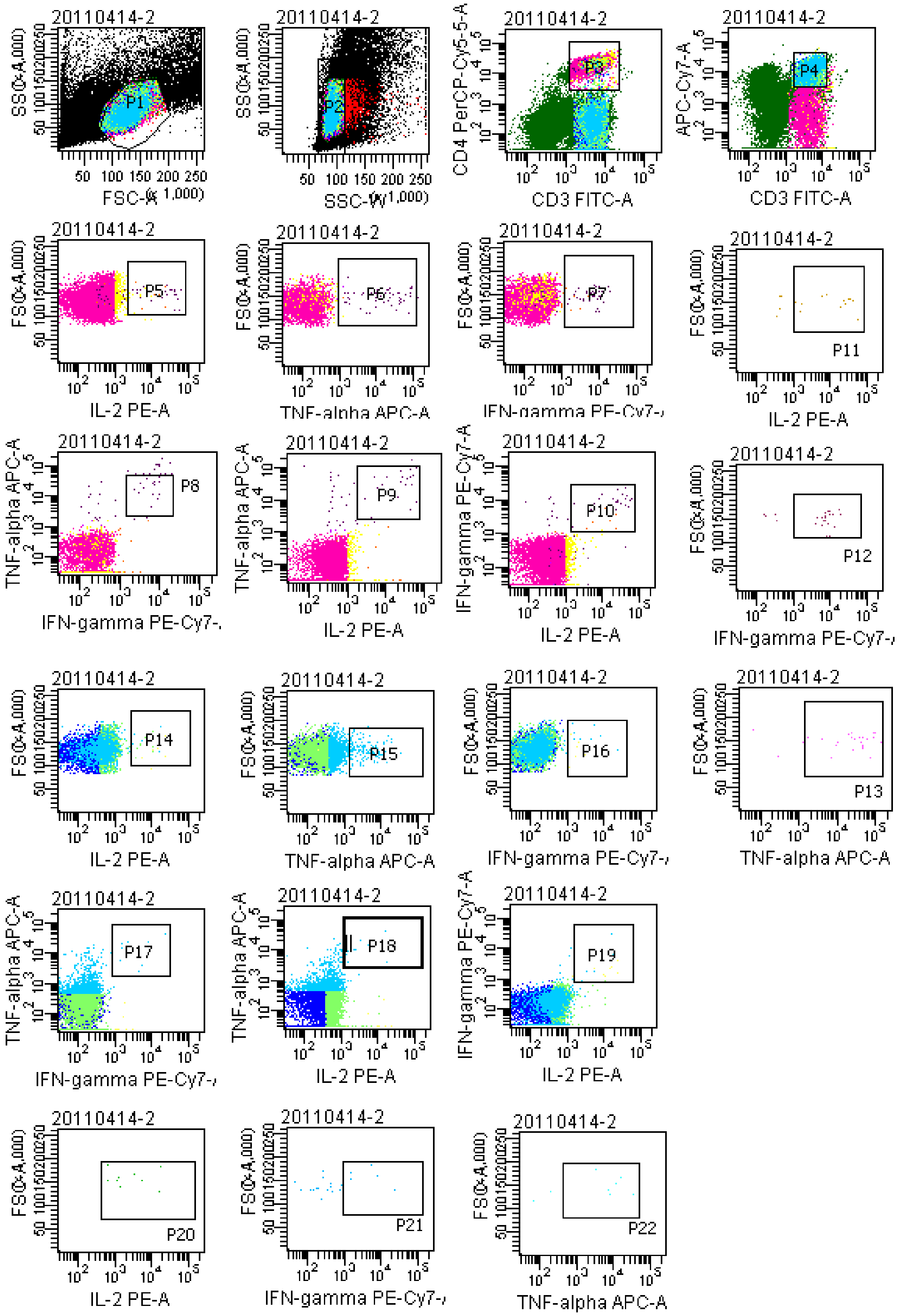

FACSDiva Version 6.0

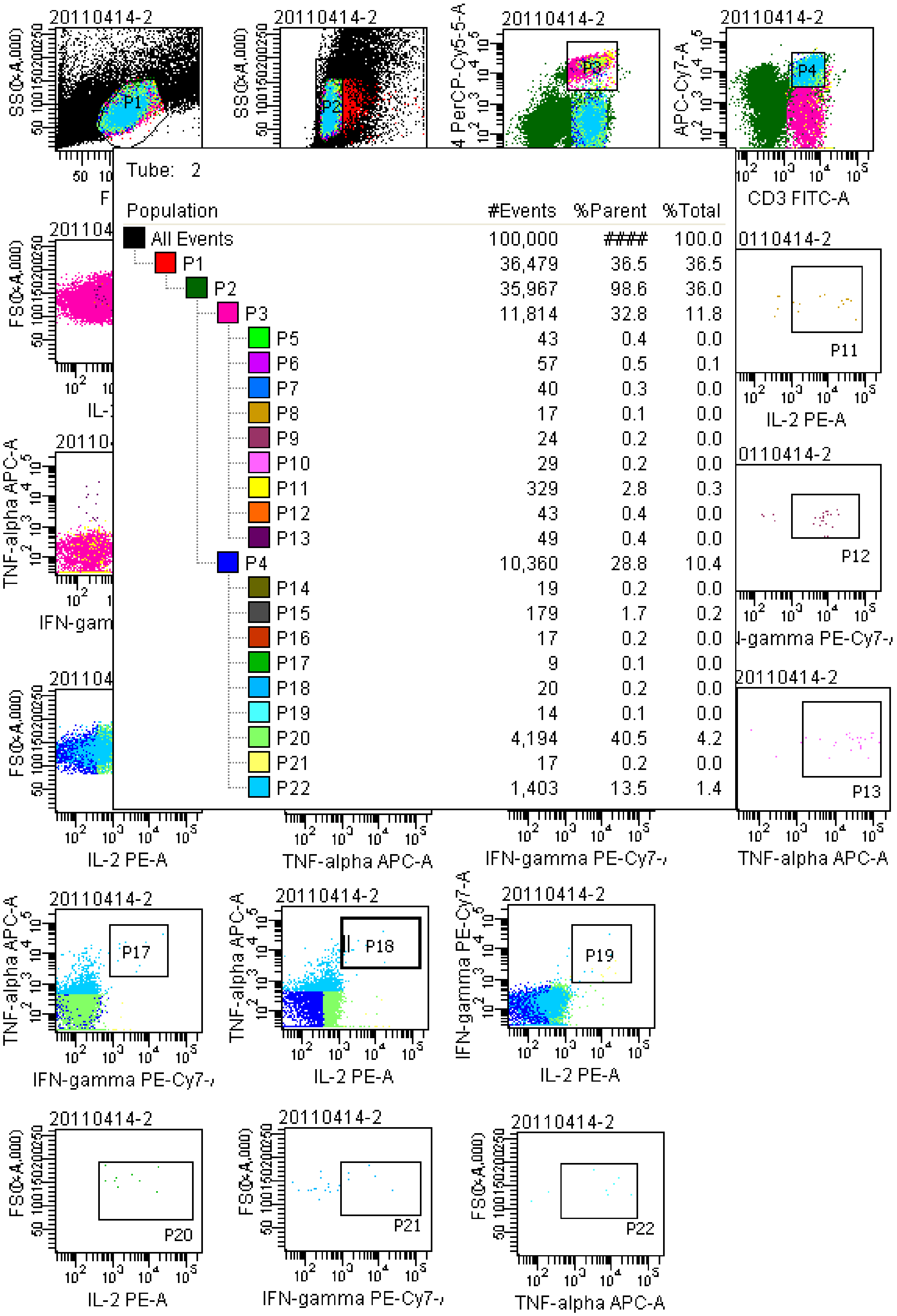

Tube: 2

Population	#Events	%Parent	%Total
All Events	100,000	####	100.0
P1	36,479	36.5	36.5
P2	35,967	98.6	36.0
P3	11,814	32.8	11.8
P5	43	0.4	0.0
P6	57	0.5	0.1
P7	40	0.3	0.0
P8	17	0.1	0.0
P9	24	0.2	0.0
P10	29	0.2	0.0
P11	329	2.8	0.3
P12	43	0.4	0.0
P13	49	0.4	0.0
P4	10,360	28.8	10.4
P14	19	0.2	0.0
P15	179	1.7	0.2
P16	17	0.2	0.0
P17	9	0.1	0.0
P18	20	0.2	0.0
P19	14	0.1	0.0
P20	4,194	40.5	4.2
P21	17	0.2	0.0
P22	1,403	13.5	1.4

248W-4vector:00R0019 (negative)

FACSDiva Version 6.0

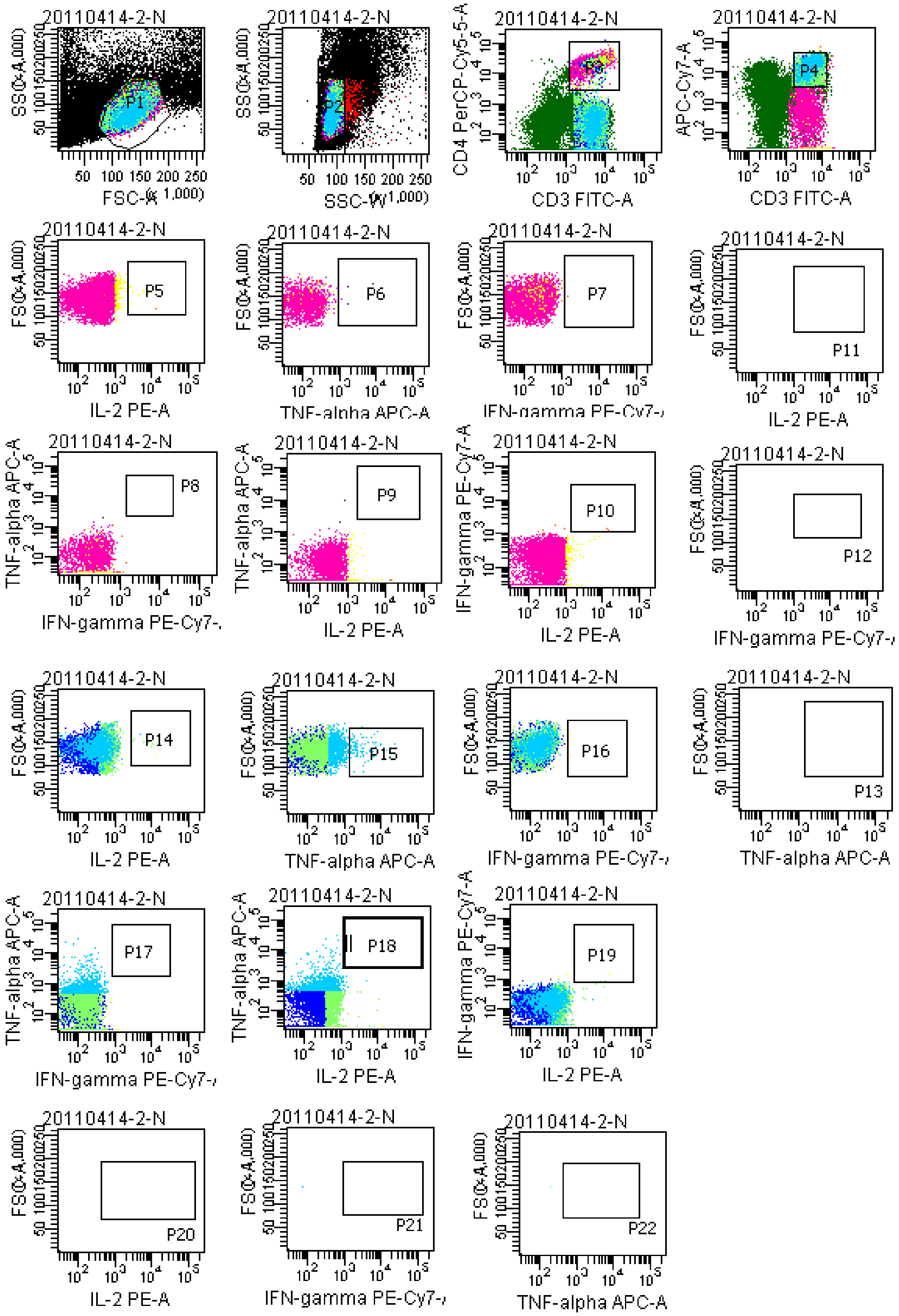

248W-4vector:00R0019 (negative)

FACSDiva Version 6.0

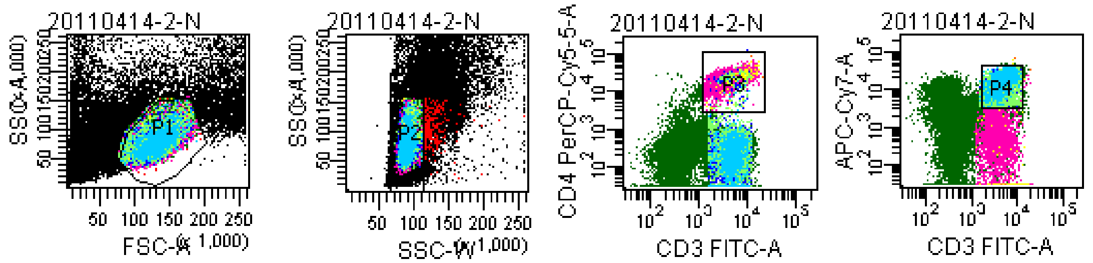

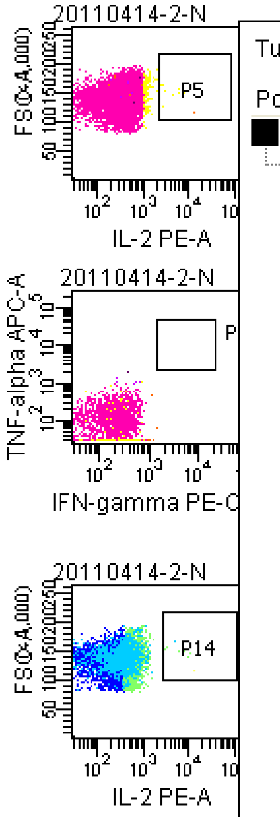

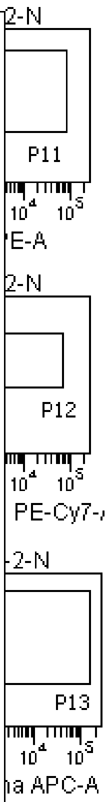

Tube: 2-N

Population	#Events	%Parent	%Total
All Events	100,000	####	100.0
P1	35,196	35.2	35.2
P2	34,850	99.0	34.8
P3	10,169	29.2	10.2
P5	14	0.1	0.0
P6	9	0.1	0.0
P7	6	0.1	0.0
P8	0	0.0	0.0
P9	0	0.0	0.0
P10	3	0.0	0.0
P11	138	1.4	0.1
P12	9	0.1	0.0
P13	3	0.0	0.0
P4	10,520	30.2	10.5
P14	11	0.1	0.0
P15	109	1.0	0.1
P16	4	0.0	0.0
P17	0	0.0	0.0
P18	3	0.0	0.0
P19	4	0.0	0.0
P20	3,800	36.1	3.8
P21	5	0.0	0.0
P22	1,420	13.5	1.4

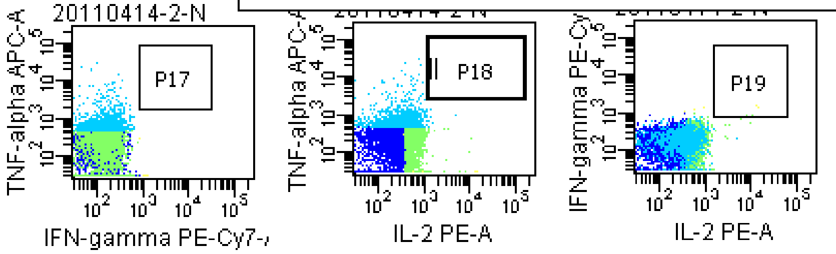

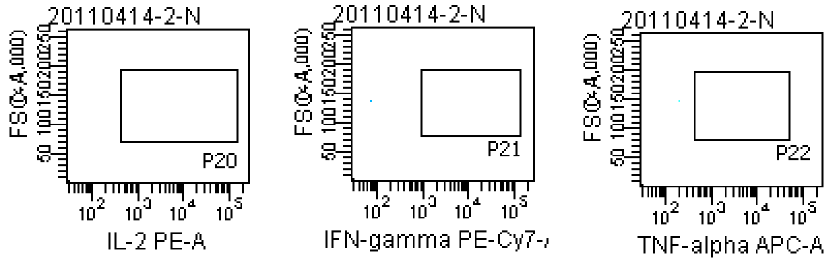

248W-2vector:98R0037 (peptide)

FACSDiva Version 6.0

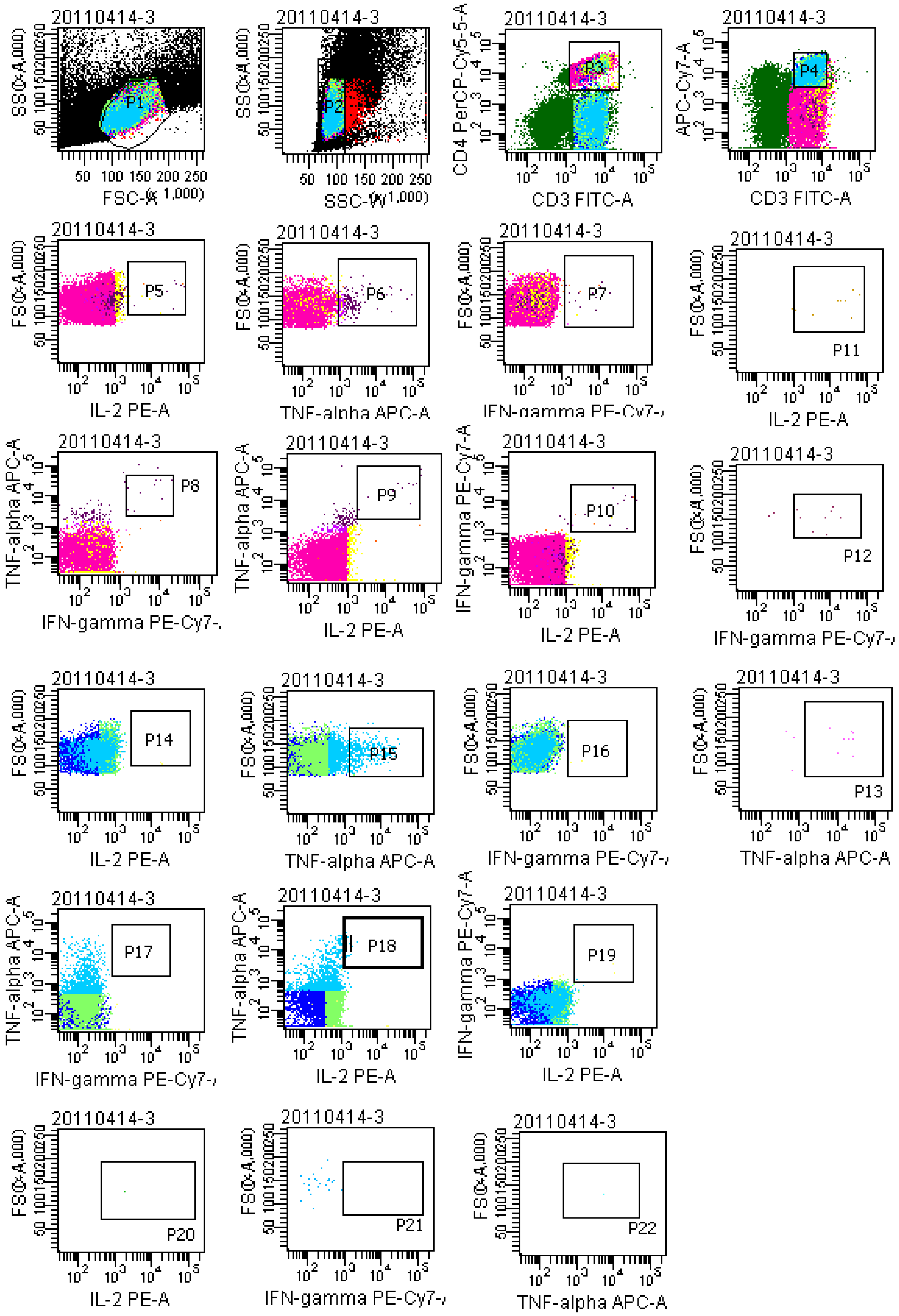

FACSDiva Version 6.0

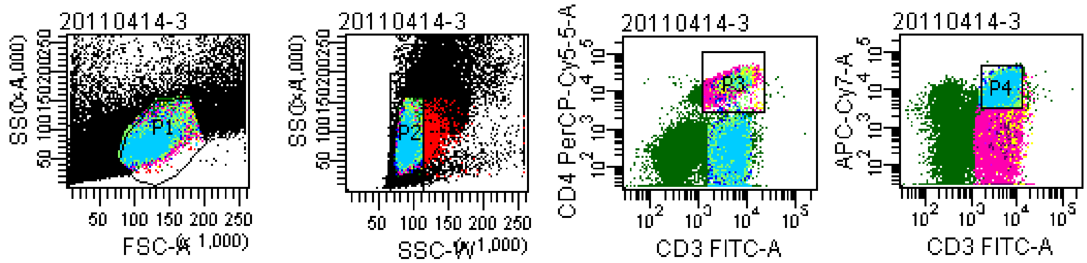

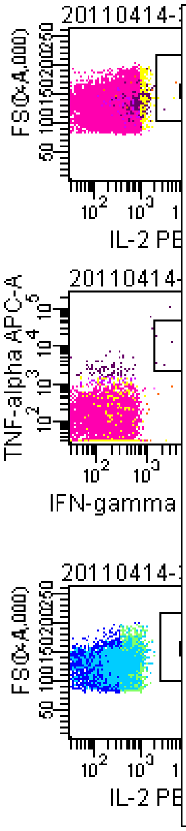

Tube: 3

Population	#Events	%Parent	%Total
All Events	100,000	####	100.0
P1	52,791	52.8	52.8
P2	51,901	98.3	51.9
P3	24,880	47.9	24.9
P5	20	0.1	0.0
P6	215	0.9	0.2
P7	19	0.1	0.0
P8	9	0.0	0.0
P9	10	0.0	0.0
P10	12	0.0	0.0
P11	387	1.6	0.4
P12	21	0.1	0.0
P13	119	0.5	0.1
P4	12,582	24.2	12.6
P14	2	0.0	0.0
P15	387	3.1	0.4
P16	5	0.0	0.0
P17	1	0.0	0.0
P18	26	0.2	0.0
P19	3	0.0	0.0
P20	4,947	39.3	4.9
P21	6	0.0	0.0
P22	1,592	12.7	1.6

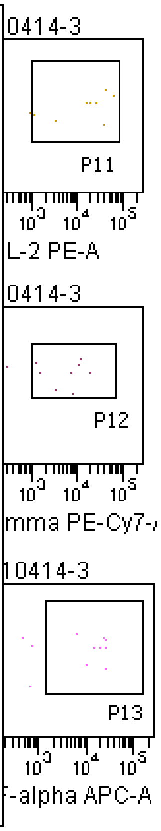

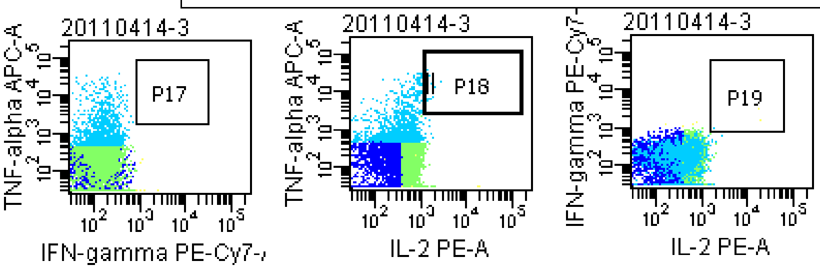

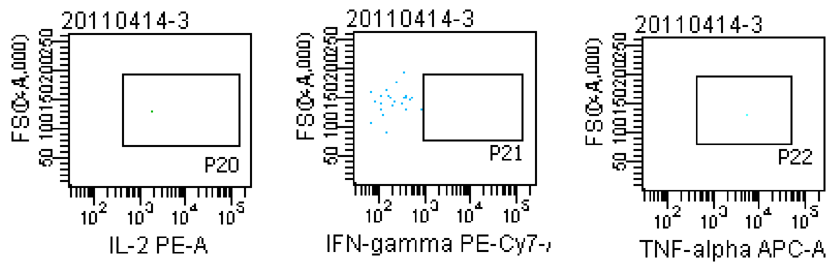

FACSDiva Version 6.0

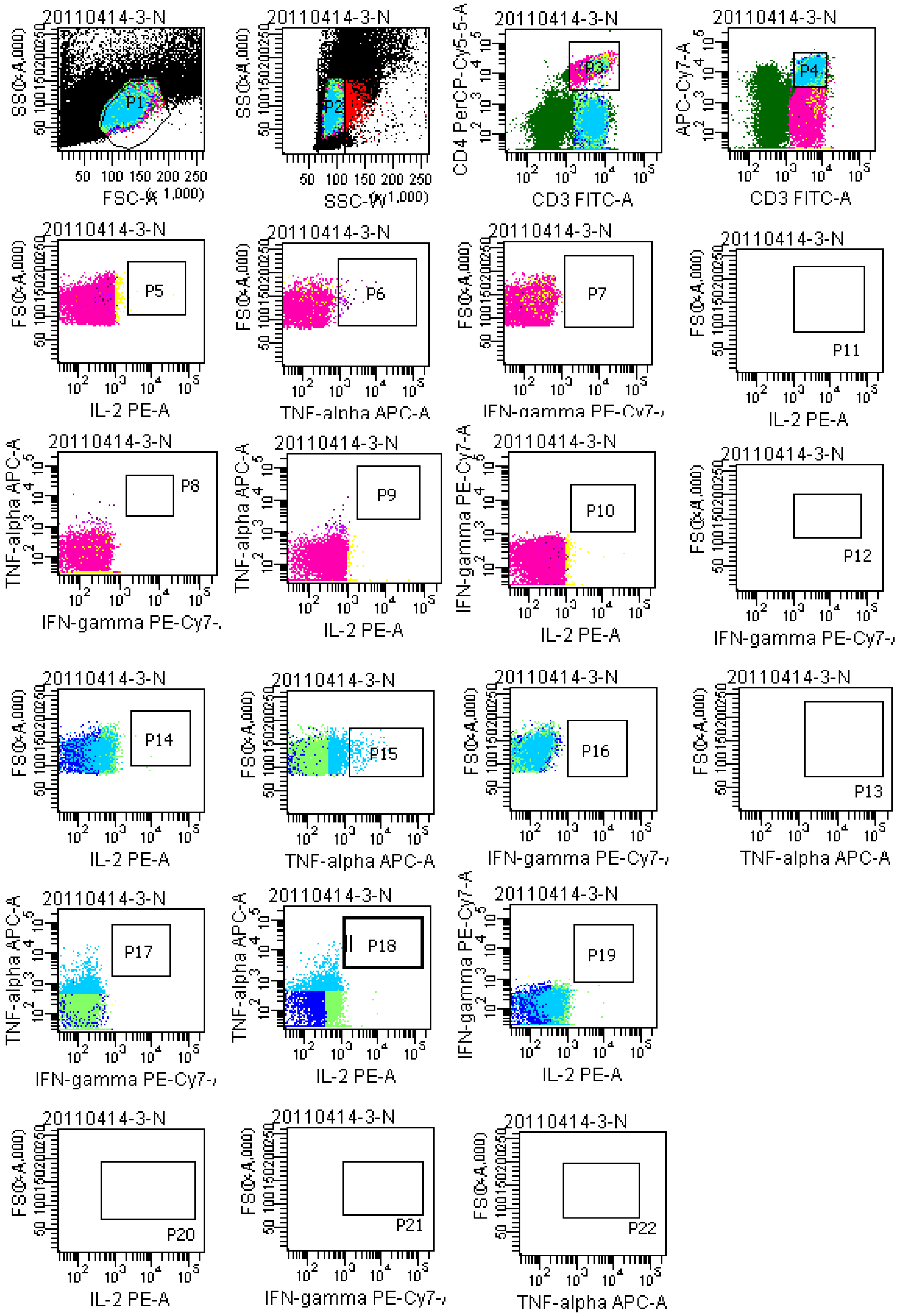

248W-2vector:98R0037 (negative)

FACSDiva Version 6.0

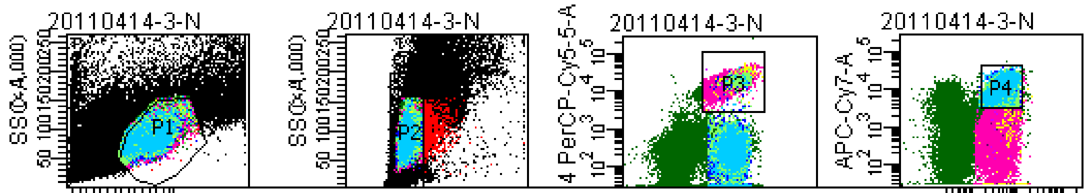

Tube: 3-N

Population	#Events	%Parent	%Total
All Events	100,000	####	100.0
P1	43,432	43.4	43.4
P2	42,628	98.1	42.6
P3	17,410	40.8	17.4
P5	4	0.0	0.0
P6	55	0.3	0.1
P7	0	0.0	0.0
P8	0	0.0	0.0
P9	0	0.0	0.0
P10	0	0.0	0.0
P11	179	1.0	0.2
P12	3	0.0	0.0
P13	18	0.1	0.0
P4	10,176	23.9	10.2
P14	2	0.0	0.0
P15	165	1.6	0.2
P16	1	0.0	0.0
P17	0	0.0	0.0
P18	1	0.0	0.0
P19	0	0.0	0.0
P20	4,027	39.6	4.0
P21	2	0.0	0.0
P22	1,470	14.4	1.5

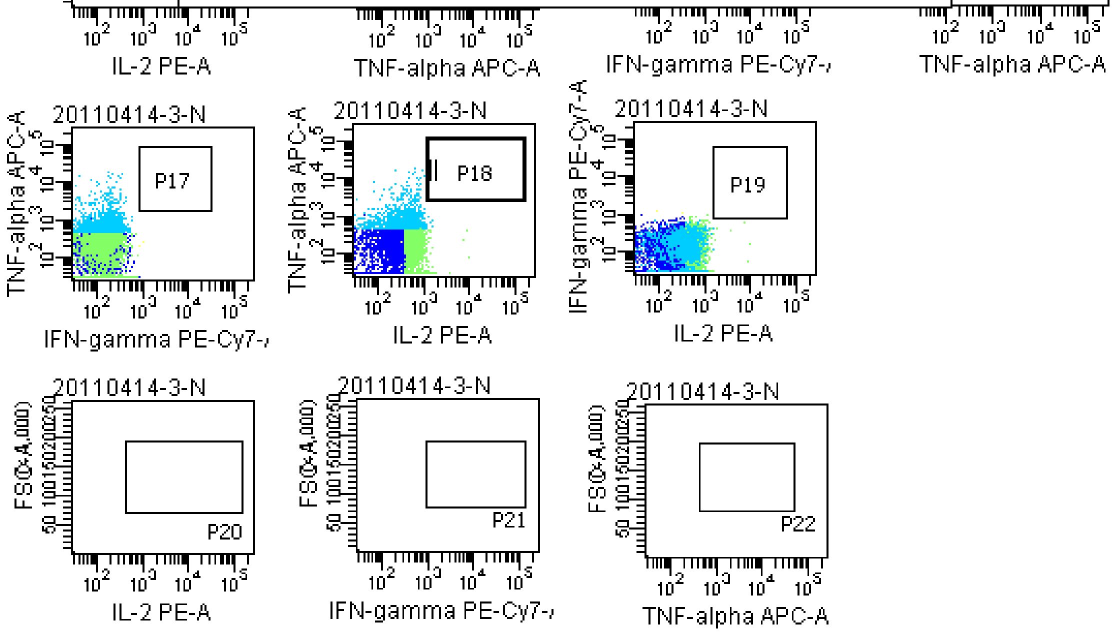

248W-4vector:99R0035 (peptide)

FACSDiva Version 6.0

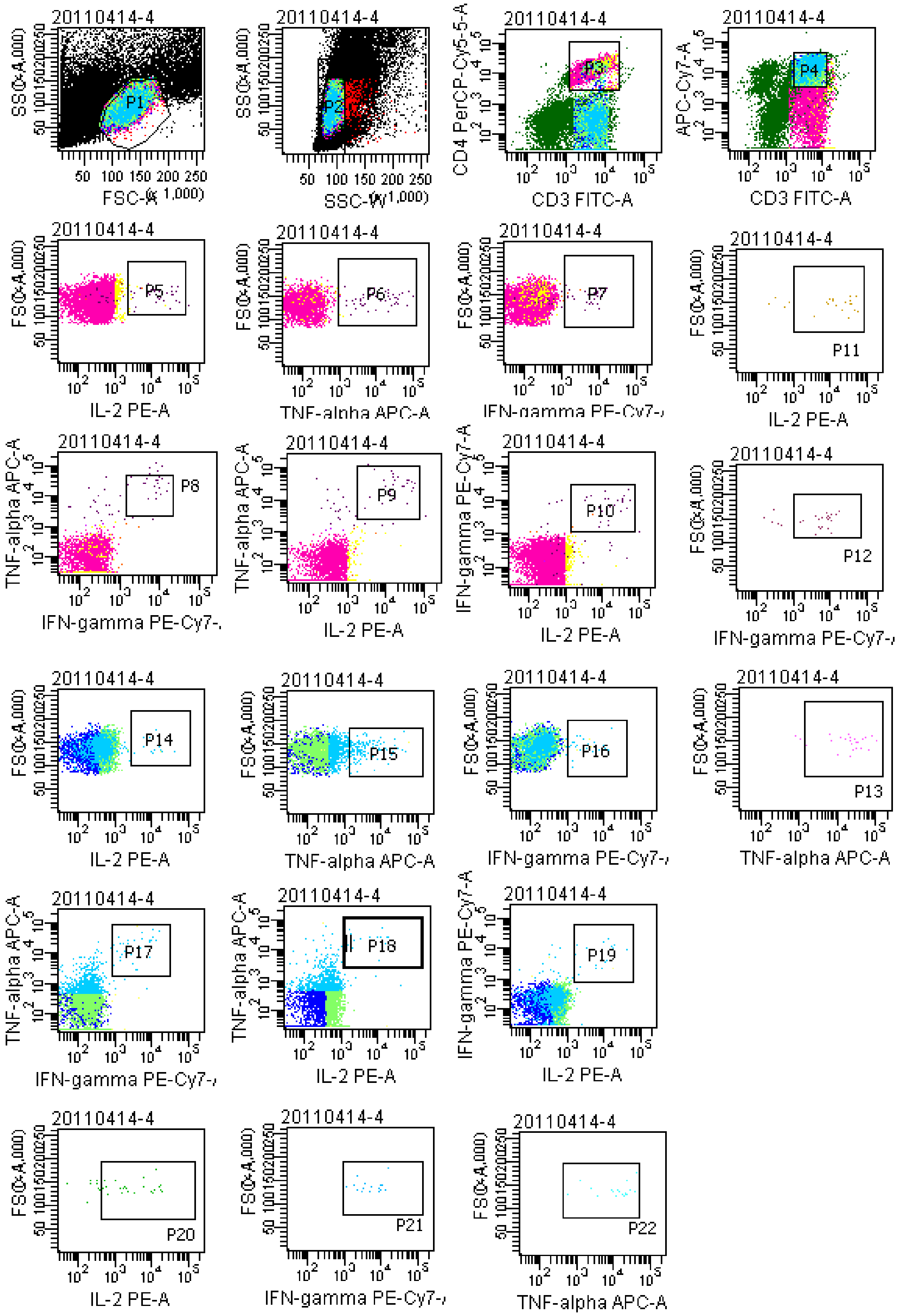

FACSDiva Version 6.0

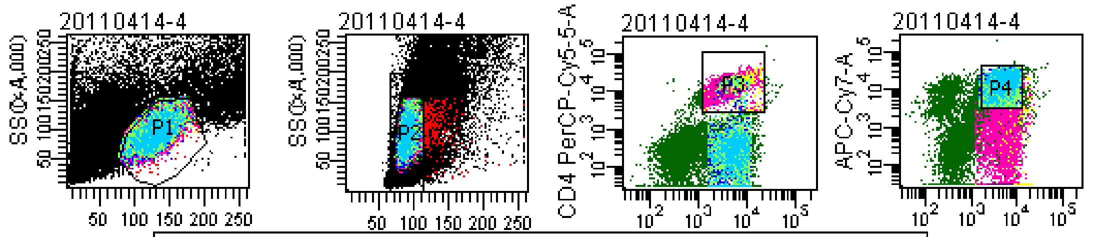

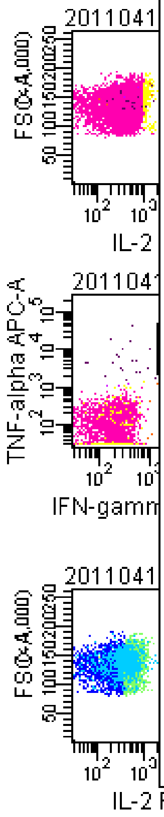

Tube: 4

Population	#Events	%Parent	%Total
All Events	100,000	####	100.0
P1	22,558	22.6	22.6
P2	22,203	98.4	22.2
P3	9,059	40.8	9.1
P5	43	0.5	0.0
P6	59	0.7	0.1
P7	31	0.3	0.0
P8	21	0.2	0.0
P9	27	0.3	0.0
P10	26	0.3	0.0
P11	264	2.9	0.3
P12	36	0.4	0.0
P13	52	0.6	0.1
P4	6,675	30.1	6.7
P14	14	0.2	0.0
P15	162	2.4	0.2
P16	35	0.5	0.0
P17	31	0.5	0.0
P18	17	0.3	0.0
P19	20	0.3	0.0
P20	2,864	42.9	2.9
P21	39	0.6	0.0
P22	927	13.9	0.9

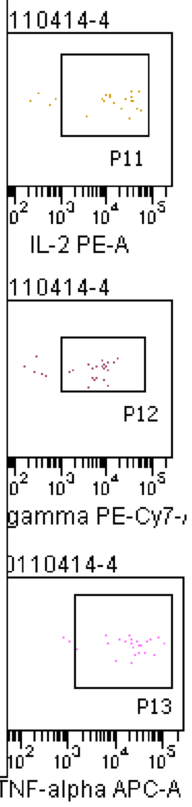

TNF-alpha APC-A IFN-gamma PE-Cy7-

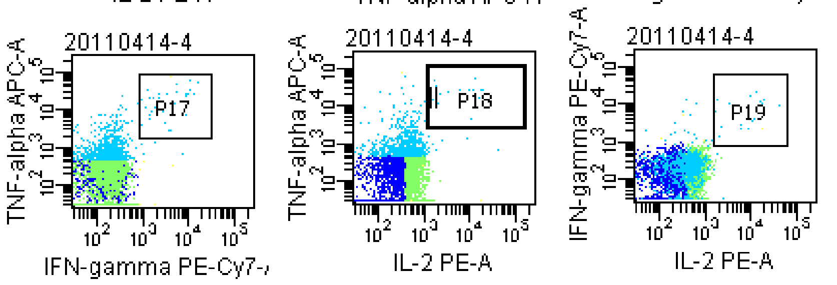

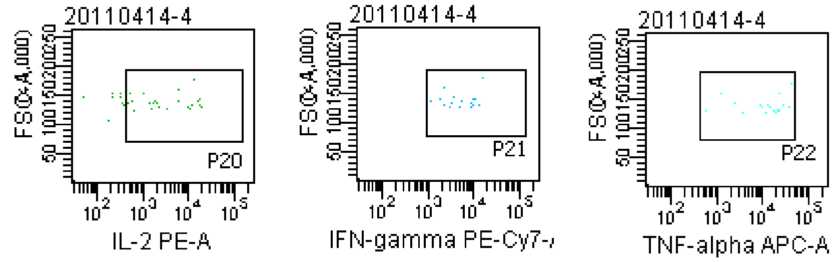

248W-4vector:99R0035 (negative)

FACSDiva Version 6.0

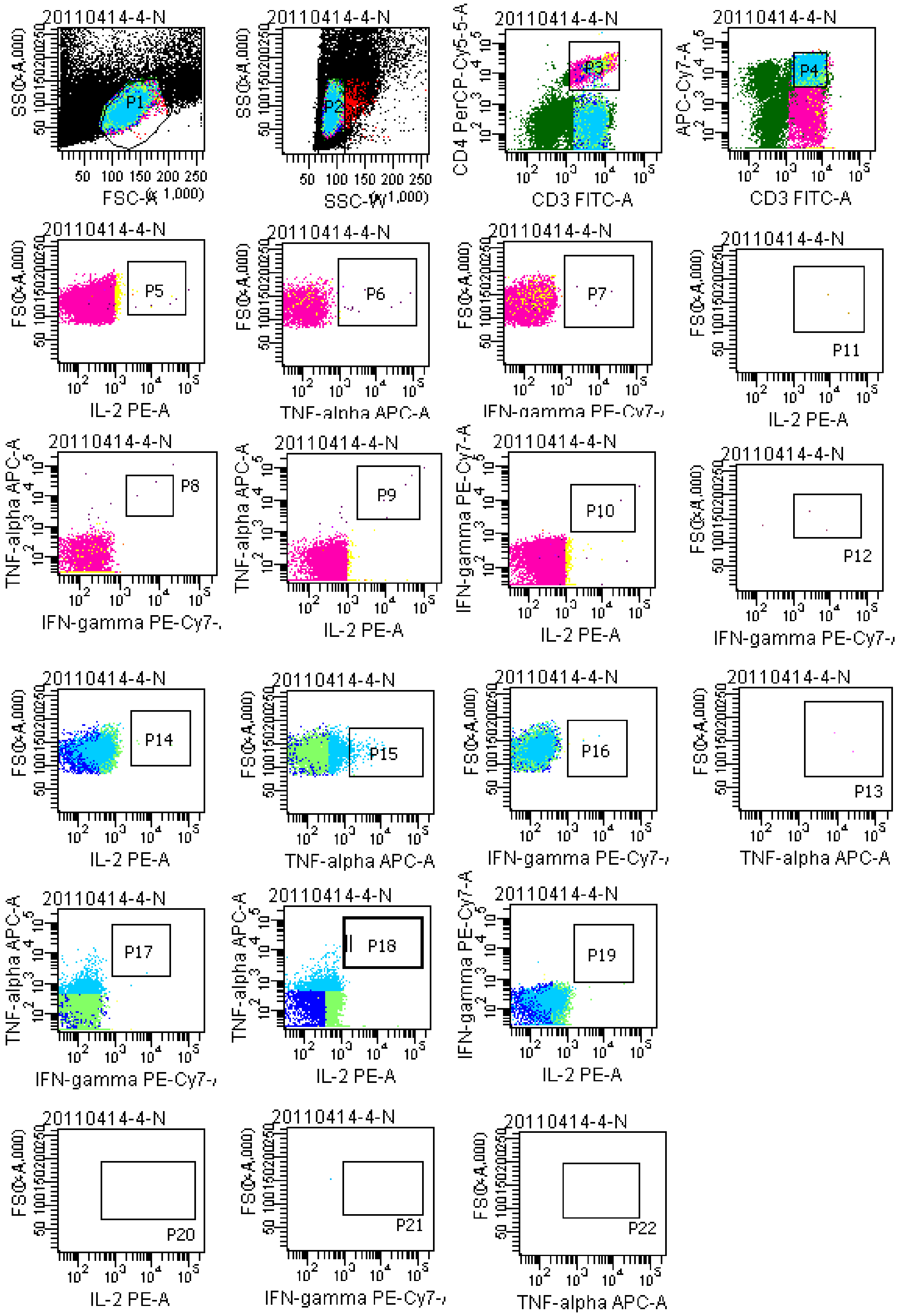

248W-4vector:99R0035 (negative)

FACSDiva Version 6.0

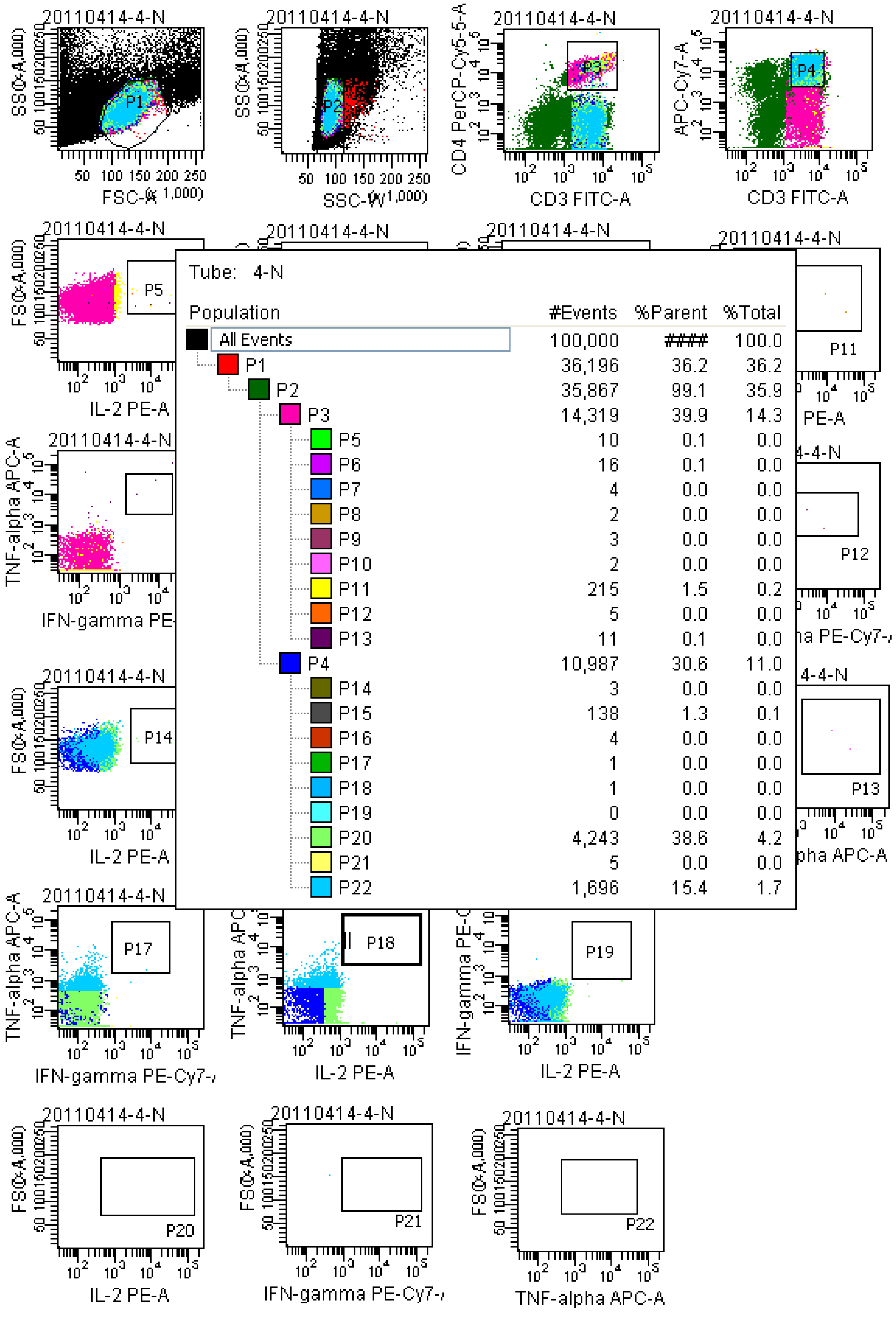

Tube: 4-N

Population	#Events	%Parent	%Total
All Events	100,000	####	100.0
P1	36,196	36.2	36.2
P2	35,867	99.1	35.9
P3	14,319	39.9	14.3
P5	10	0.1	0.0
P6	16	0.1	0.0
P7	4	0.0	0.0
P8	2	0.0	0.0
P9	3	0.0	0.0
P10	2	0.0	0.0
P11	215	1.5	0.2
P12	5	0.0	0.0
P13	11	0.1	0.0
P4	10,987	30.6	11.0
P14	3	0.0	0.0
P15	138	1.3	0.1
P16	4	0.0	0.0
P17	1	0.0	0.0
P18	1	0.0	0.0
P19	0	0.0	0.0
P20	4,243	38.6	4.2
P21	5	0.0	0.0
P22	1,696	15.4	1.7

248W-4vector:00R0227 (peptide)

FACSDiva Version 6.0

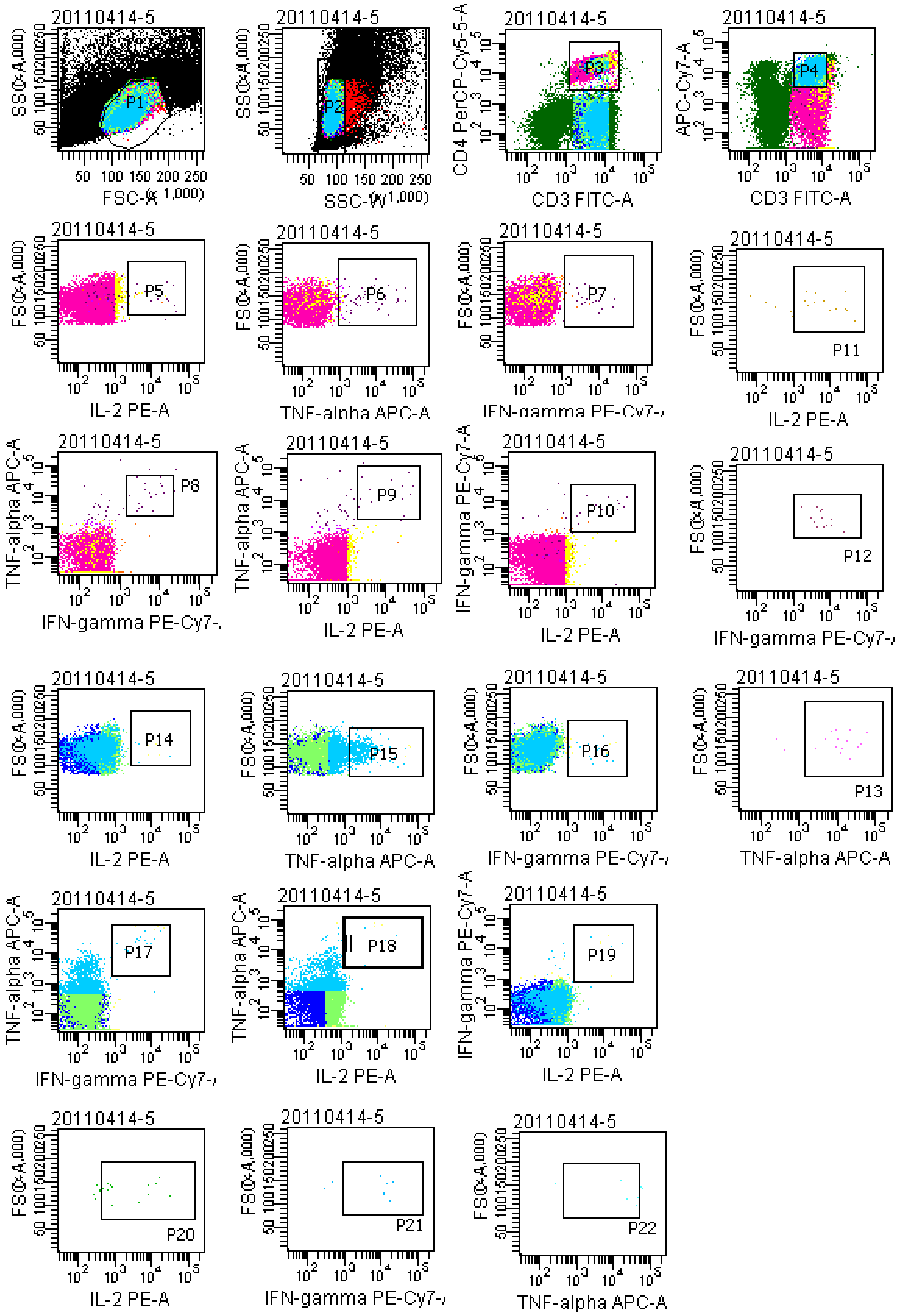

248W-4vector:00R0227 (peptide)

FACSDiva Version 6.0

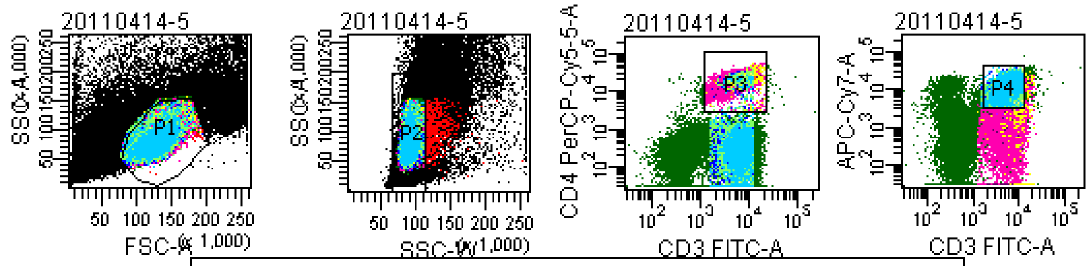

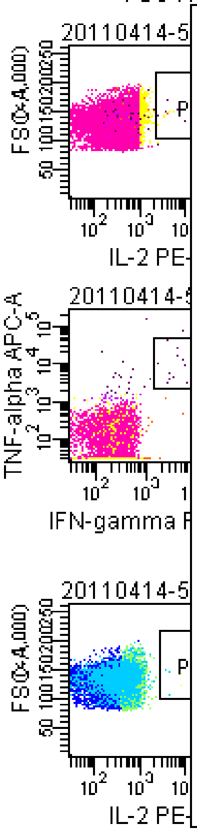

Tube: 5

Population	#Events	%Parent	%Total
All Events	100,000	####	100.0
P1	42,462	42.5	42.5
P2	41,774	98.4	41.8
P3	14,177	33.9	14.2
P5	25	0.2	0.0
P6	78	0.6	0.1
P7	29	0.2	0.0
P8	18	0.1	0.0
P9	14	0.1	0.0
P10	17	0.1	0.0
P11	352	2.5	0.4
P12	34	0.2	0.0
P13	45	0.3	0.0
P4	14,881	35.6	14.9
P14	10	0.1	0.0
P15	460	3.1	0.5
P16	27	0.2	0.0
P17	19	0.1	0.0
P18	8	0.1	0.0
P19	8	0.1	0.0
P20	6,277	42.2	6.3
P21	33	0.2	0.0
P22	2,274	15.3	2.3

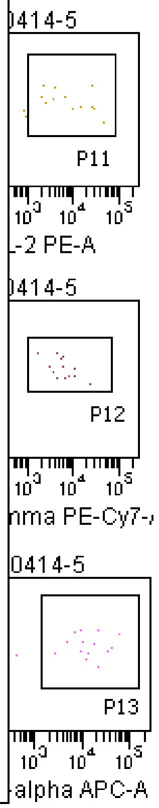

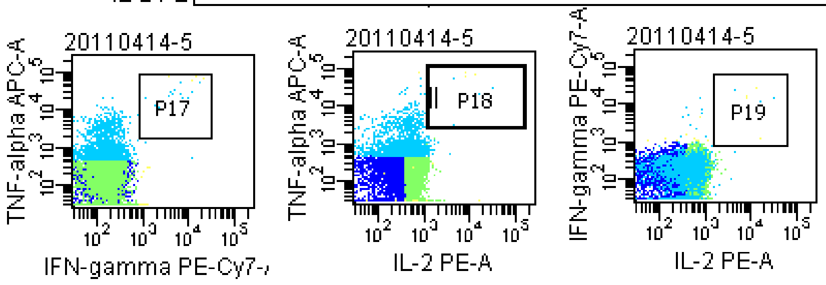

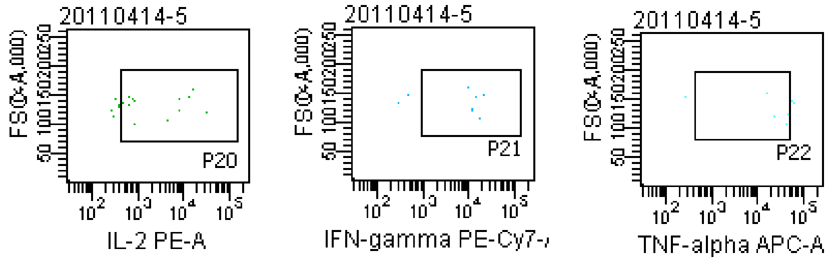

248W-4vector:00R0227 (negative)

FACSDiva Version 6.0

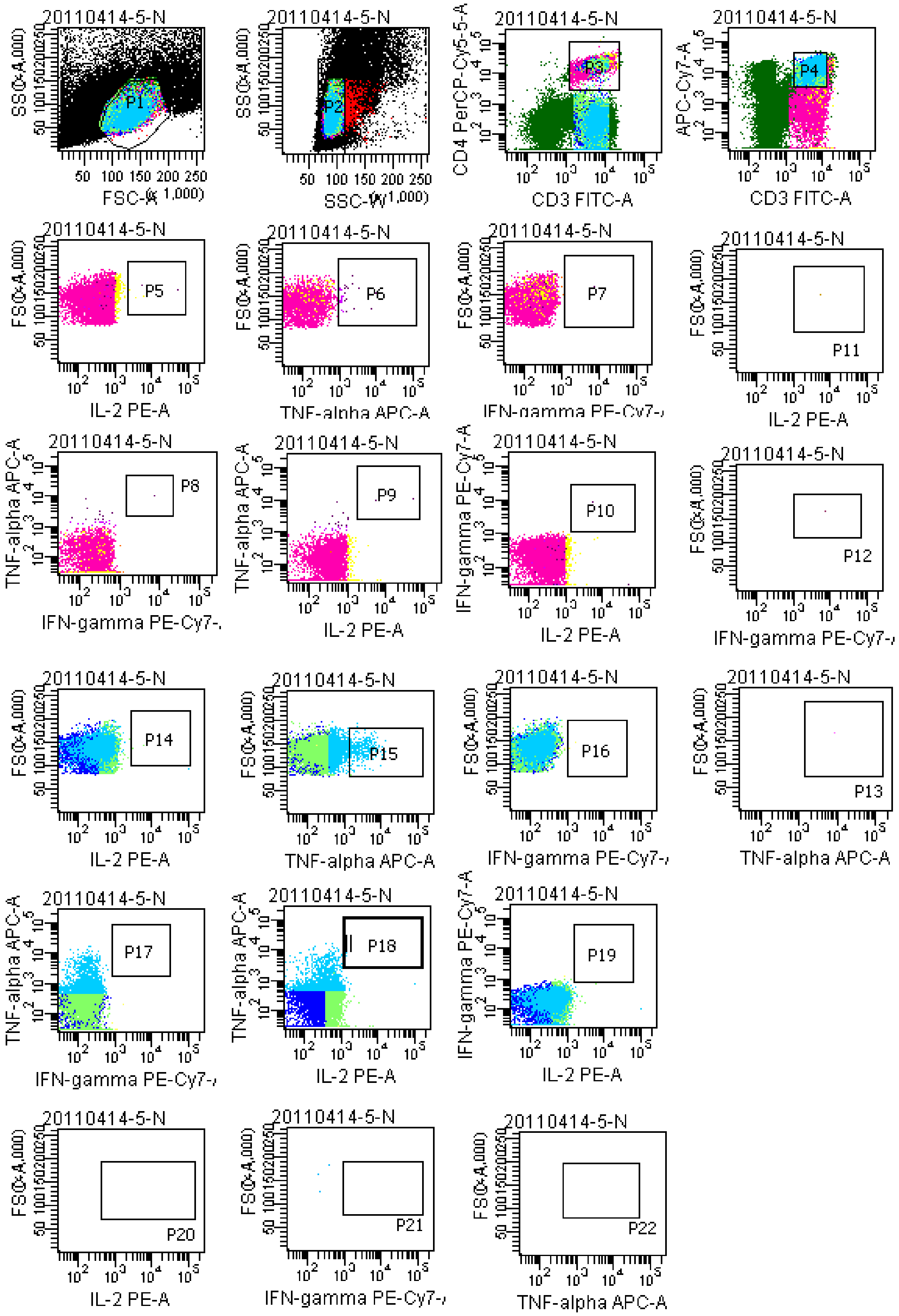

248W-4vector:00R0227 (negative)

FACSDiva Version 6.0

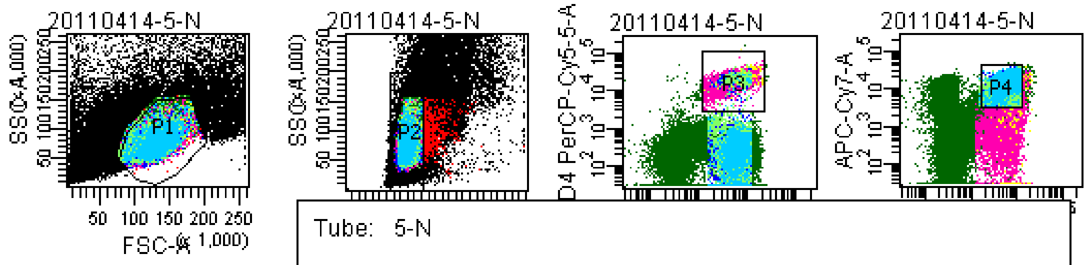

Tube: 5-N

Population	#Events	%Parent	%Total
All Events	100,000	####	100.0
P1	39,843	39.8	39.8
P2	39,251	98.5	39.3
P3	10,964	27.9	11.0
P5	6	0.1	0.0
P6	33	0.3	0.0
P7	2	0.0	0.0
P8	1	0.0	0.0
P9	2	0.0	0.0
P10	1	0.0	0.0
P11	184	1.7	0.2
P12	5	0.0	0.0
P13	16	0.1	0.0
P4	13,310	33.9	13.3
P14	3	0.0	0.0
P15	384	2.9	0.4
P16	3	0.0	0.0
P17	0	0.0	0.0
P18	3	0.0	0.0
P19	0	0.0	0.0
P20	5,176	38.9	5.2
P21	4	0.0	0.0
P22	2,353	17.7	2.4

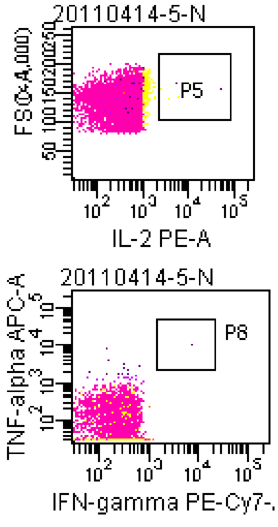

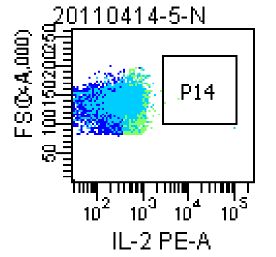

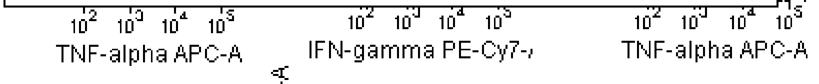

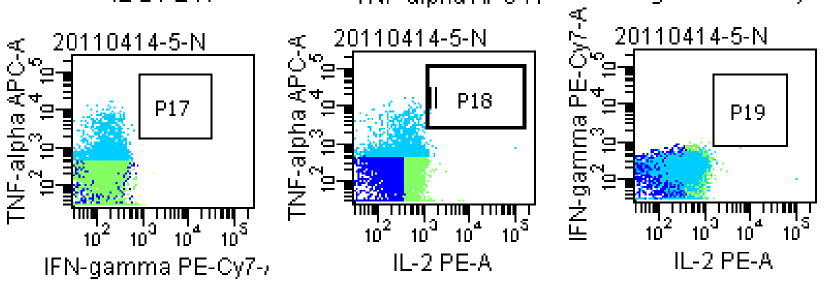

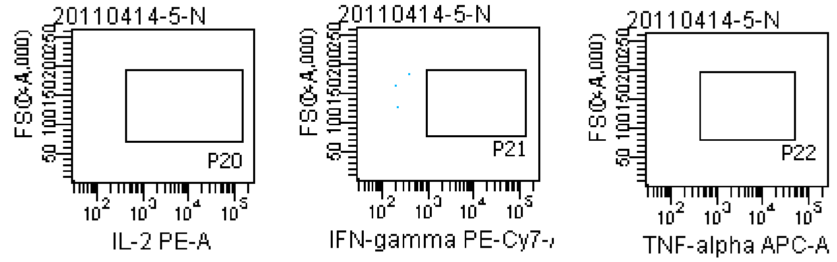

248W-4vector:98R0013 (peptide)

FACSDiva Version 6.0

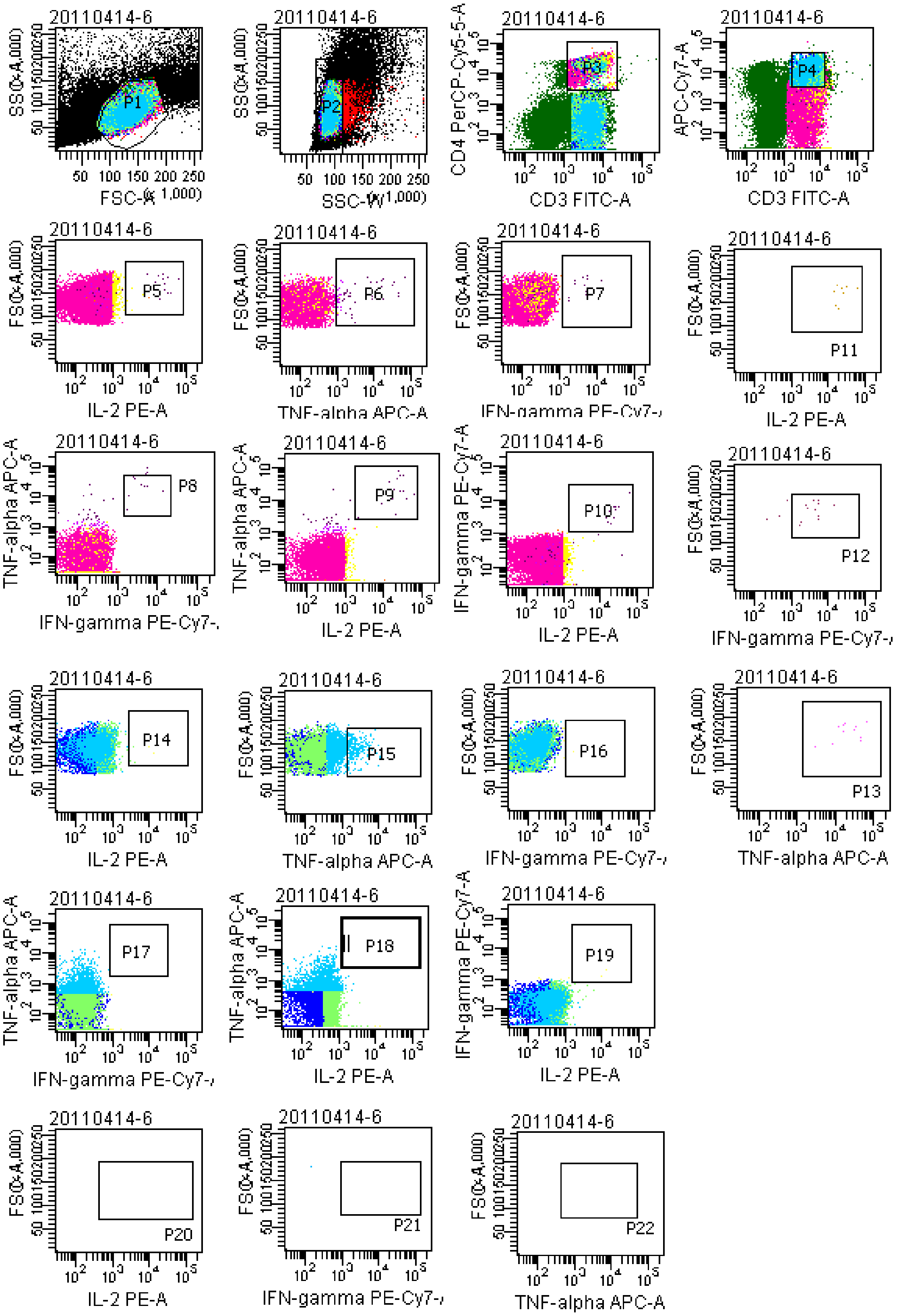

248W-4vector:98R0013 (peptide)

FACSDiva Version 6.0

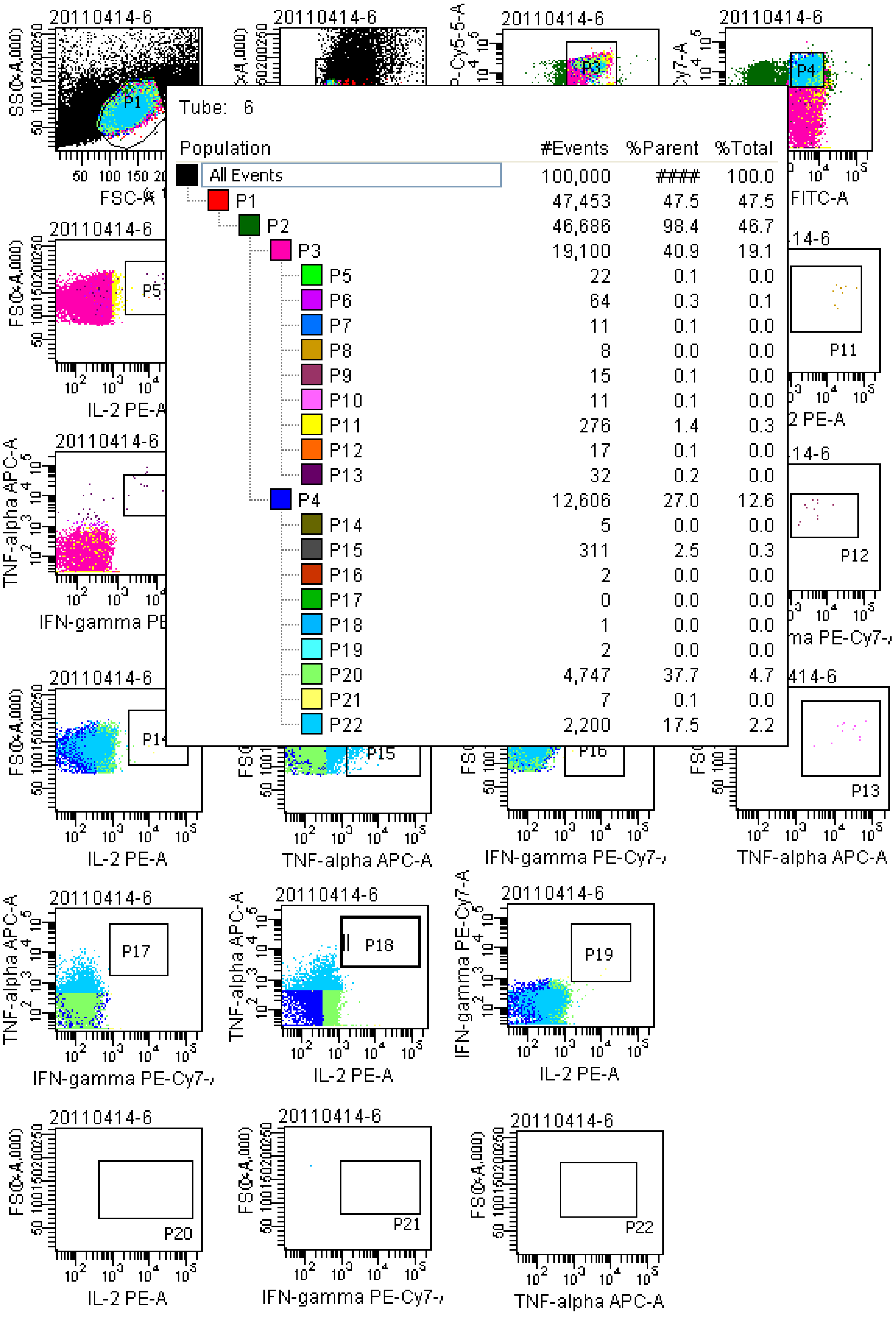

Tube: 6

Population	#Events	%Parent	%Total
All Events	100,000	####	100.0
P1	47,453	47.5	47.5
P2	46,686	98.4	46.7
P3	19,100	40.9	19.1
P5	22	0.1	0.0
P6	64	0.3	0.1
P7	11	0.1	0.0
P8	8	0.0	0.0
P9	15	0.1	0.0
P10	11	0.1	0.0
P11	276	1.4	0.3
P12	17	0.1	0.0
P13	32	0.2	0.0
P4	12,606	27.0	12.6
P14	5	0.0	0.0
P15	311	2.5	0.3
P16	2	0.0	0.0
P17	0	0.0	0.0
P18	1	0.0	0.0
P19	2	0.0	0.0
P20	4,747	37.7	4.7
P21	7	0.1	0.0
P22	2,200	17.5	2.2

248W-4vector:98R0013 (negative)

FACSDiva Version 6.0

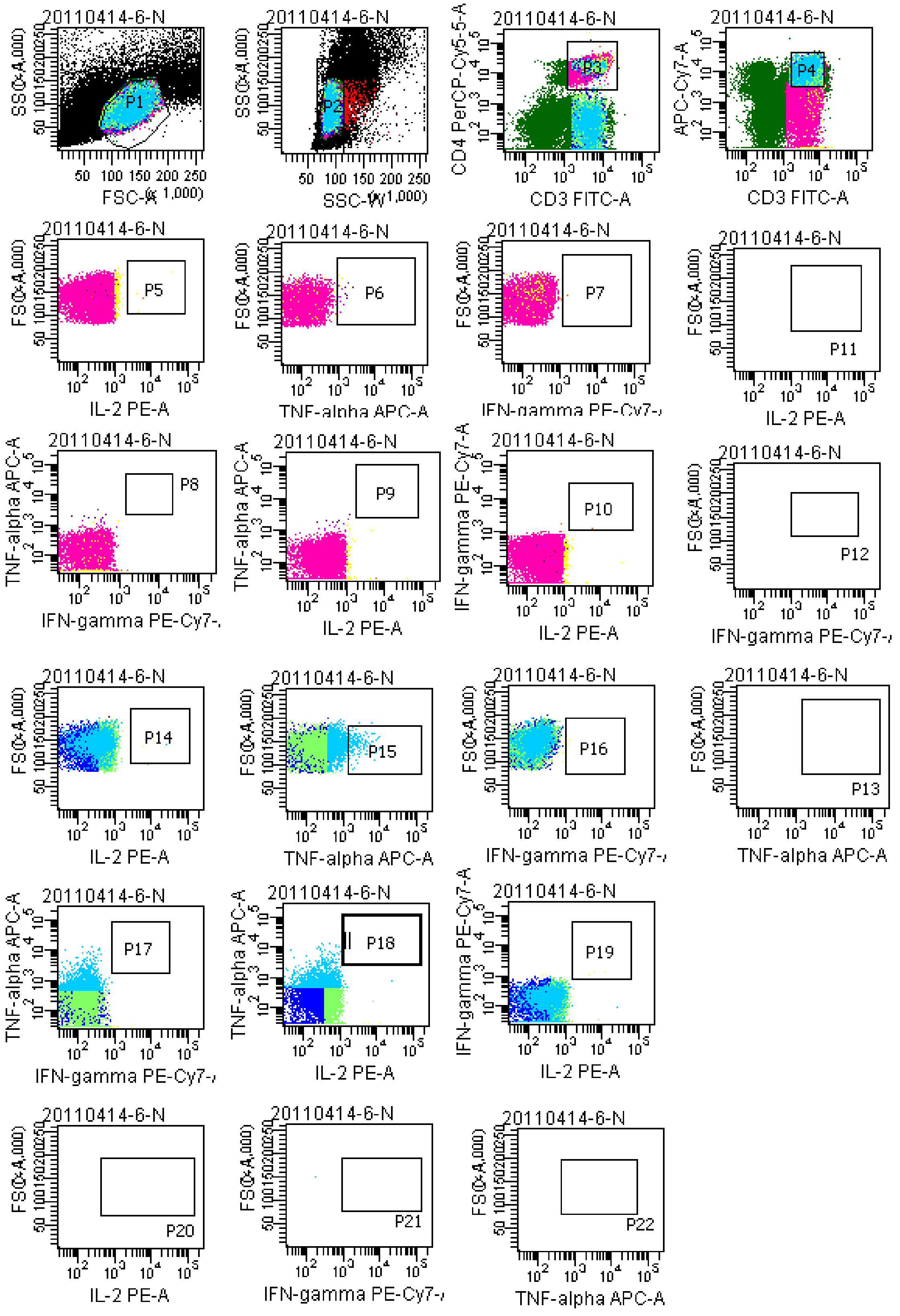

248W-4vector:98R0013 (negative)

FACSDiva Version 6.0

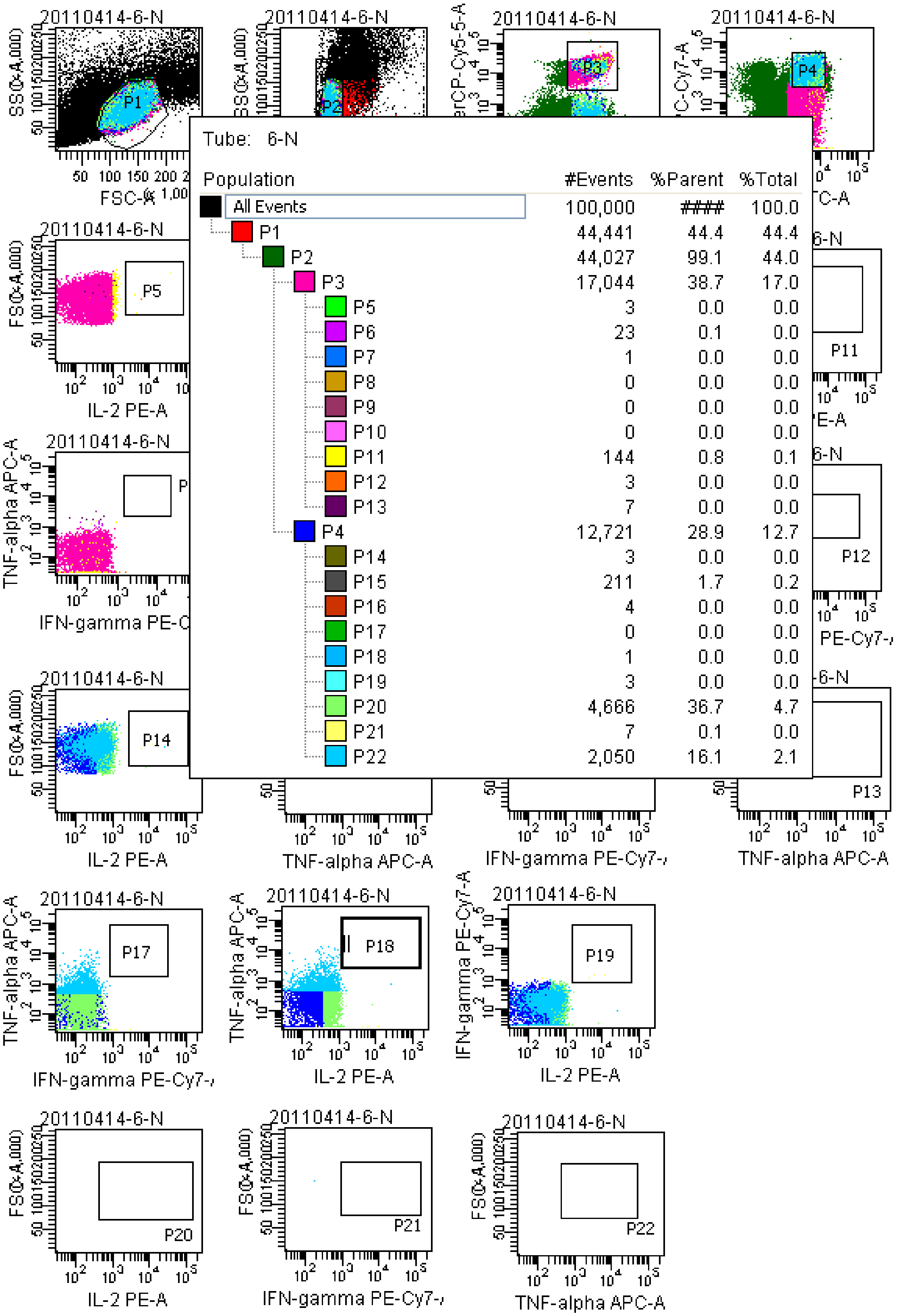

Tube: 6-N

Population	#Events	%Parent	%Total
All Events	100,000	####	100.0
P1	44,441	44.4	44.4
P2	44,027	99.1	44.0
P3	17,044	38.7	17.0
P5	3	0.0	0.0
P6	23	0.1	0.0
P7	1	0.0	0.0
P8	0	0.0	0.0
P9	0	0.0	0.0
P10	0	0.0	0.0
P11	144	0.8	0.1
P12	3	0.0	0.0
P13	7	0.0	0.0
P4	12,721	28.9	12.7
P14	3	0.0	0.0
P15	211	1.7	0.2
P16	4	0.0	0.0
P17	0	0.0	0.0
P18	1	0.0	0.0
P19	3	0.0	0.0
P20	4,666	36.7	4.7
P21	7	0.1	0.0
P22	2,050	16.1	2.1

FACSDiva Version 6.0

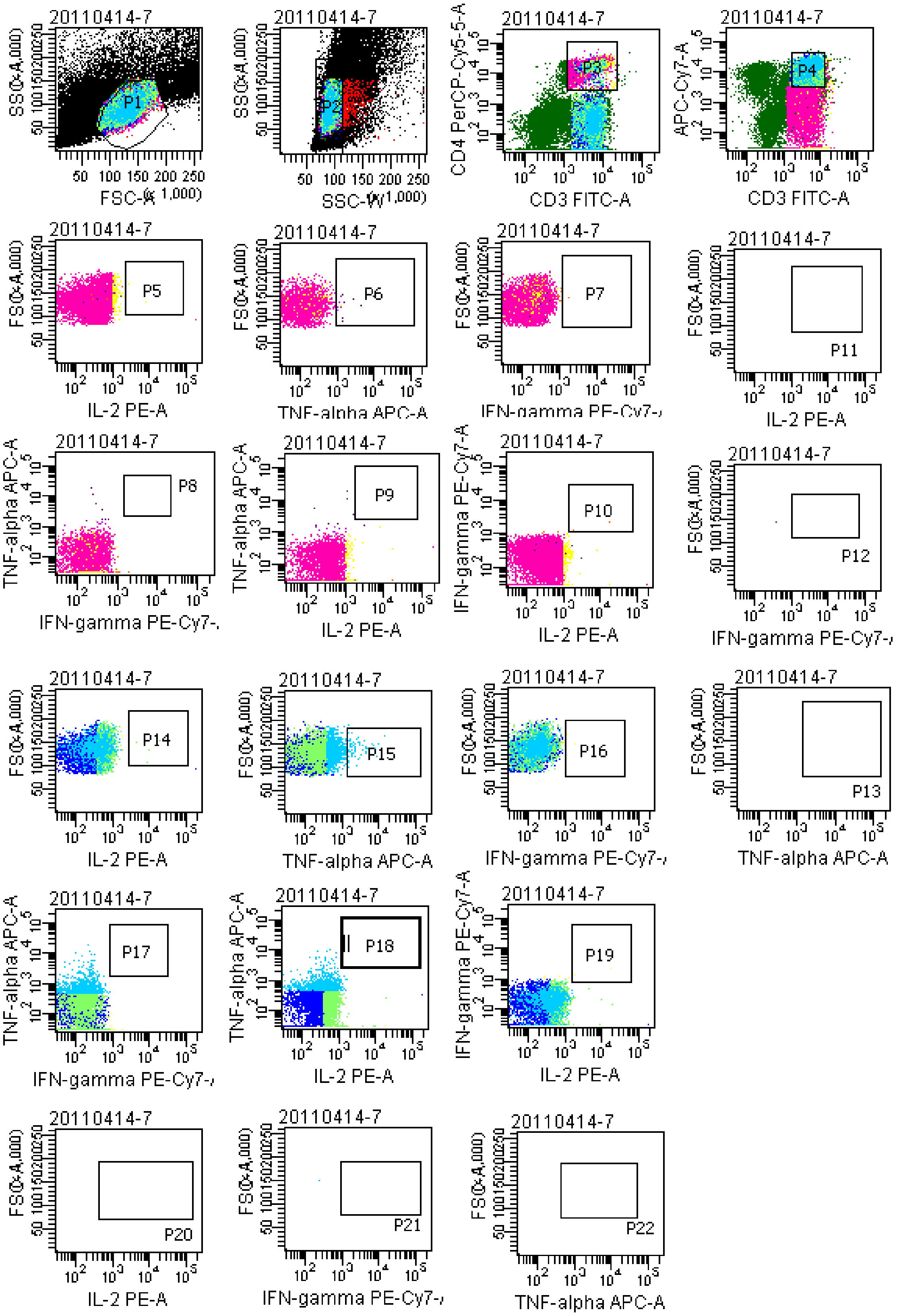

FACSDiva Version 6.0

Tube: 7

Population	#Events	%Parent	%Total
All Events	100,000	####	100.0
P1	30,247	30.2	30.2
P2	29,792	98.5	29.8
P3	12,560	42.2	12.6
P5	3	0.0	0.0
P6	17	0.1	0.0
P7	1	0.0	0.0
P8	0	0.0	0.0
P9	1	0.0	0.0
P10	1	0.0	0.0
P11	155	1.2	0.2
P12	8	0.1	0.0
P13	8	0.1	0.0
P4	9,444	31.7	9.4
P14	5	0.1	0.0
P15	91	1.0	0.1
P16	8	0.1	0.0
P17	0	0.0	0.0
P18	1	0.0	0.0
P19	2	0.0	0.0
P20	3,347	35.4	3.3
P21	12	0.1	0.0
P22	1,168	12.4	1.2

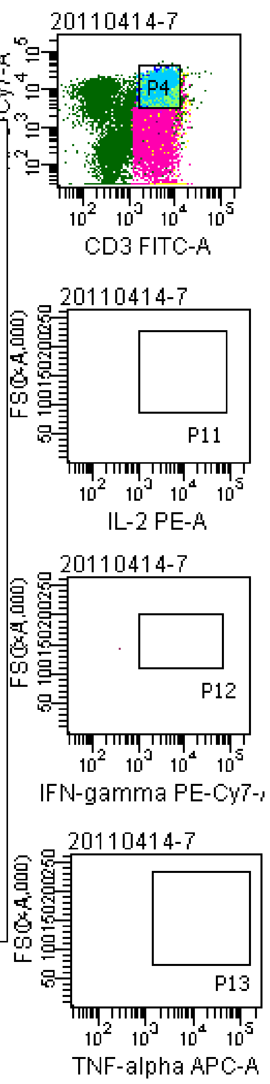

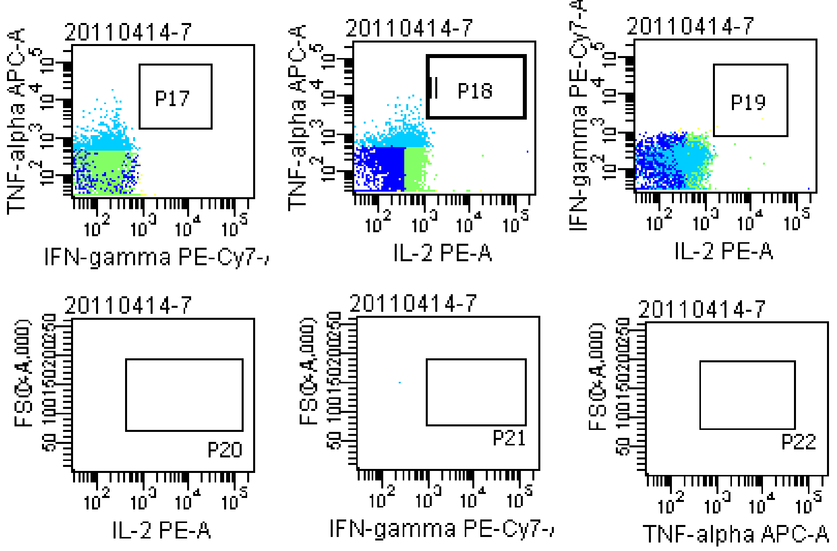

248W-control:98R0009 (negative)

FACSDiva Version 6.0

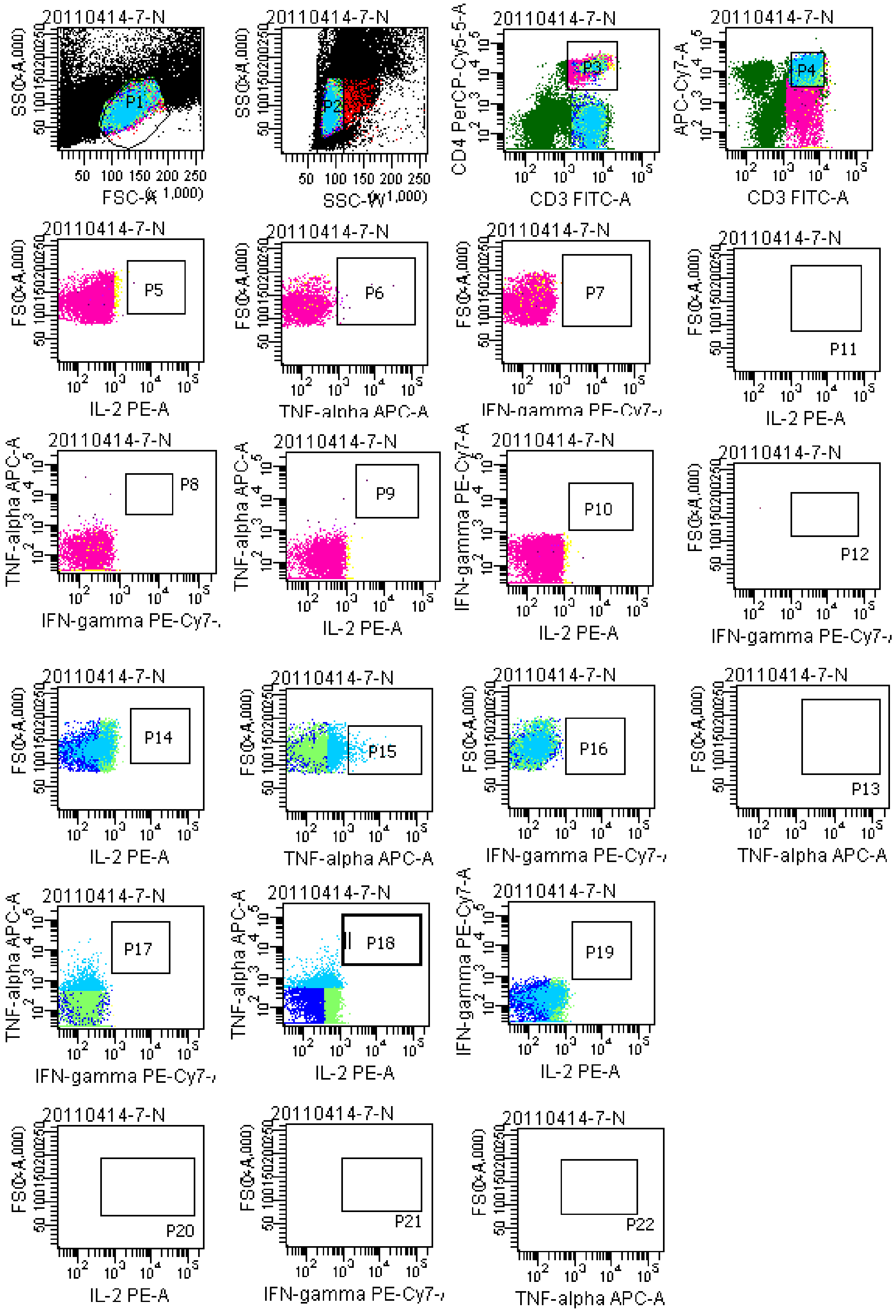

248W-control:98R0009 (negative)

FACSDiva Version 6.0

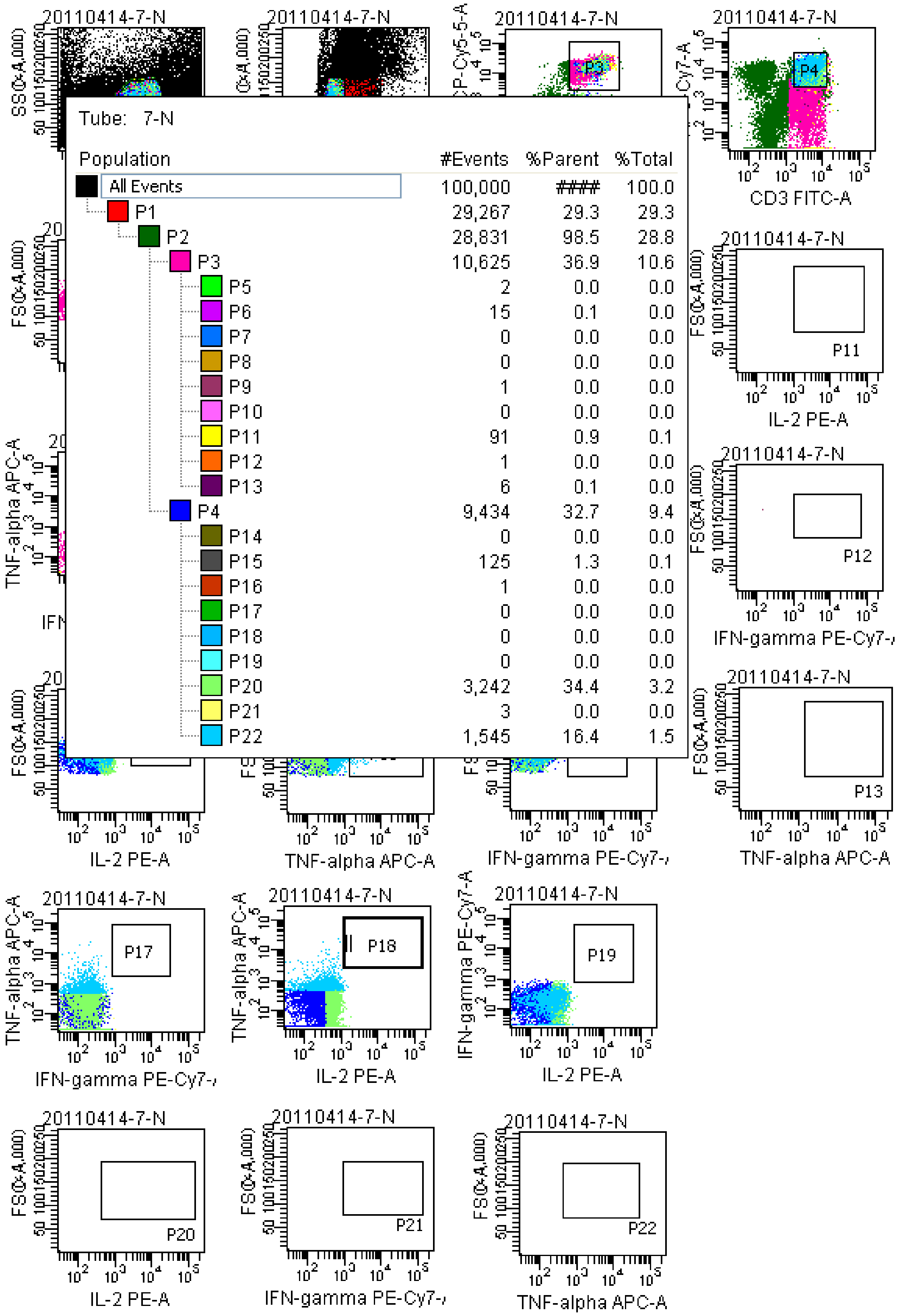

Tube: 7-N

Population	#Events	%Parent	%Total
All Events	100,000	####	100.0
P1	29,267	29.3	29.3
P2	28,831	98.5	28.8
P3	10,625	36.9	10.6
P5	2	0.0	0.0
P6	15	0.1	0.0
P7	0	0.0	0.0
P8	0	0.0	0.0
P9	1	0.0	0.0
P10	0	0.0	0.0
P11	91	0.9	0.1
P12	1	0.0	0.0
P13	6	0.1	0.0
P4	9,434	32.7	9.4
P14	0	0.0	0.0
P15	125	1.3	0.1
P16	1	0.0	0.0
P17	0	0.0	0.0
P18	0	0.0	0.0
P19	0	0.0	0.0
P20	3,242	34.4	3.2
P21	3	0.0	0.0
P22	1,545	16.4	1.5

248W-control:00R0023 (peptide)

FACSDiva Version 6.0

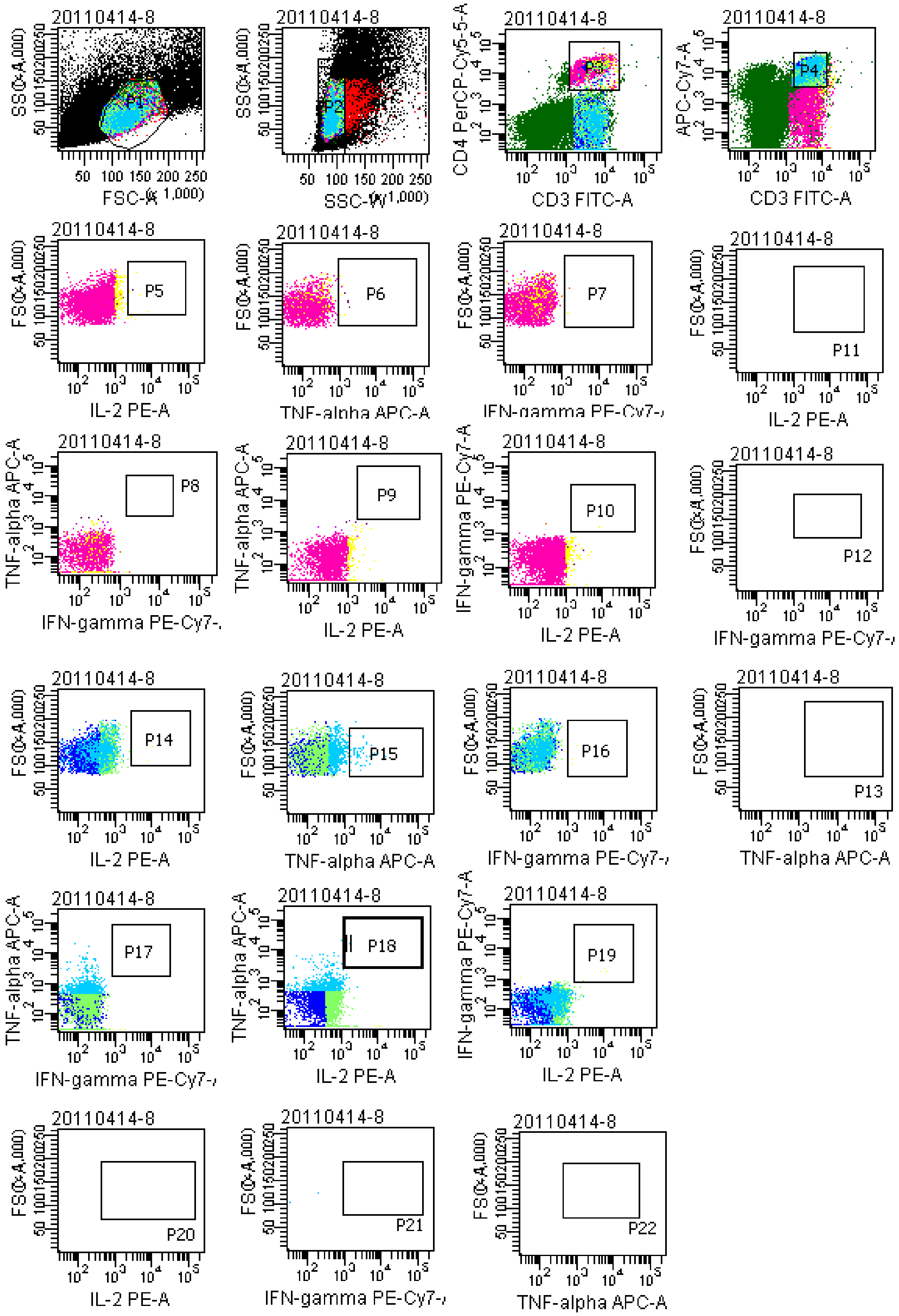

FACSDiva Version 6.0

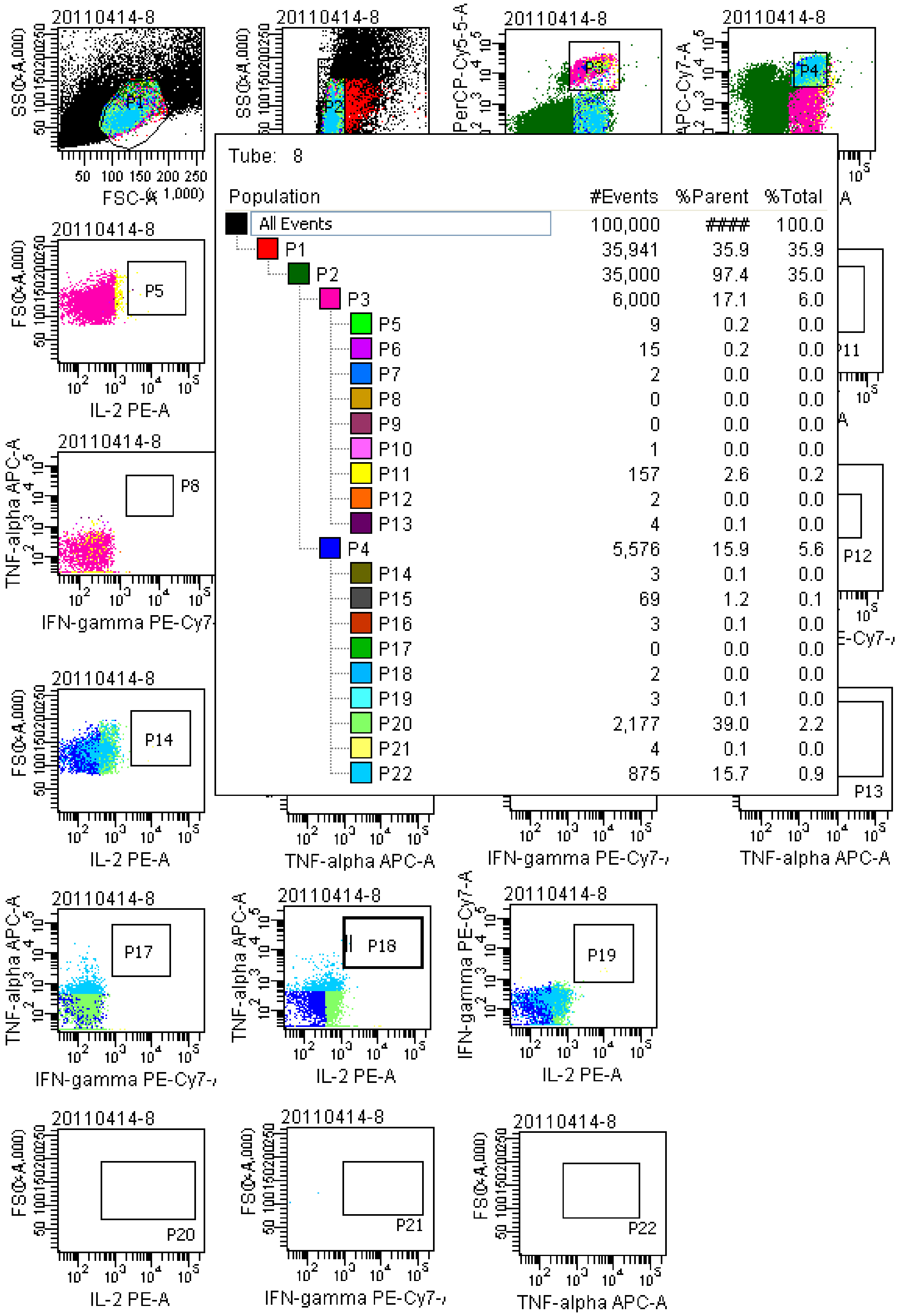

Tube: 8

Population	#Events	%Parent	%Total
All Events	100,000	####	100.0
P1	35,941	35.9	35.9
P2	35,000	97.4	35.0
P3	6,000	17.1	6.0
P5	9	0.2	0.0
P6	15	0.2	0.0
P7	2	0.0	0.0
P8	0	0.0	0.0
P9	0	0.0	0.0
P10	1	0.0	0.0
P11	157	2.6	0.2
P12	2	0.0	0.0
P13	4	0.1	0.0
P4	5,576	15.9	5.6
P14	3	0.1	0.0
P15	69	1.2	0.1
P16	3	0.1	0.0
P17	0	0.0	0.0
P18	2	0.0	0.0
P19	3	0.1	0.0
P20	2,177	39.0	2.2
P21	4	0.1	0.0
P22	875	15.7	0.9

248W-control:00R0023 (negative)

FACSDiva Version 6.0

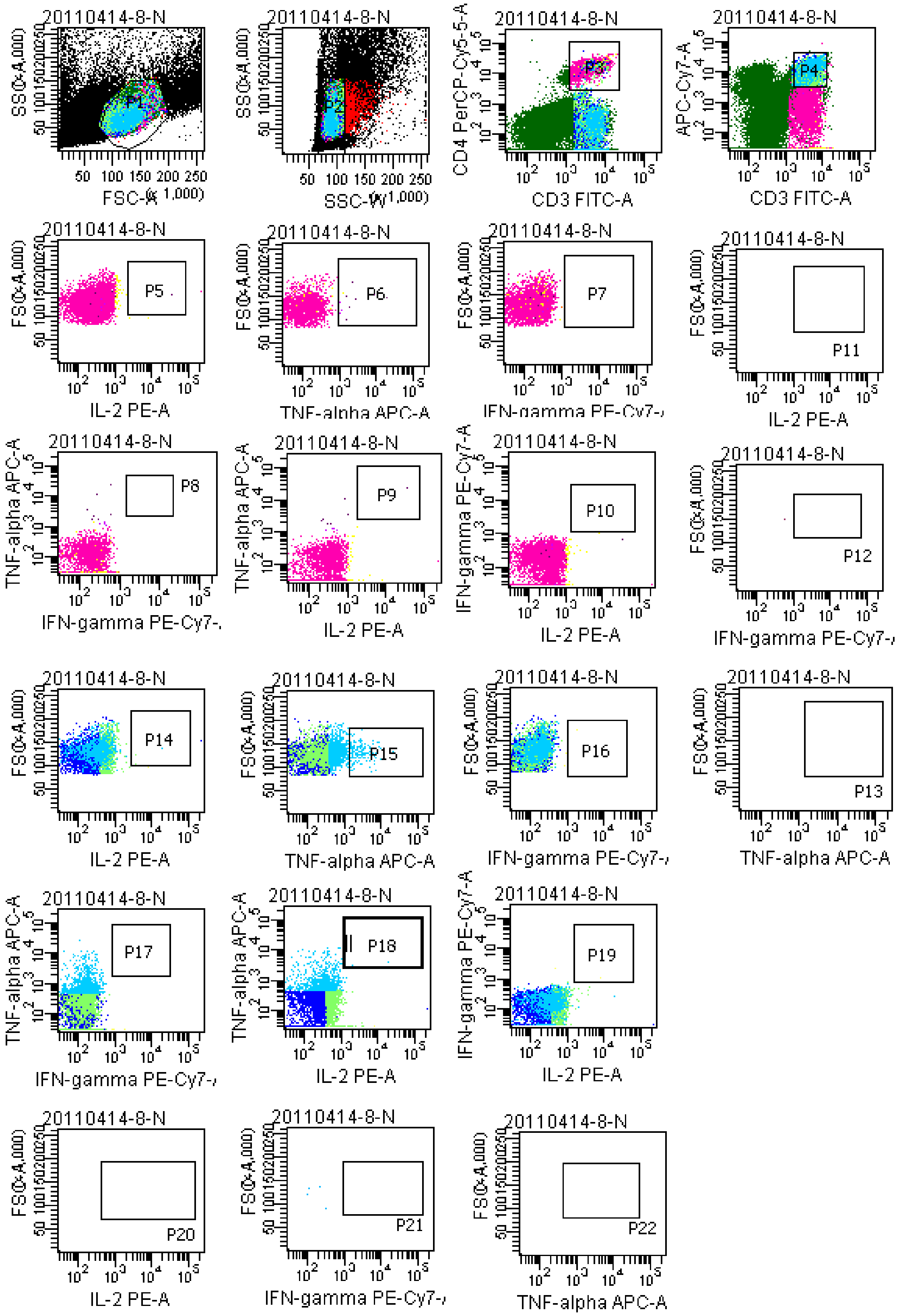

248W-control:00R0023 (negative)

FACSDiva Version 6.0

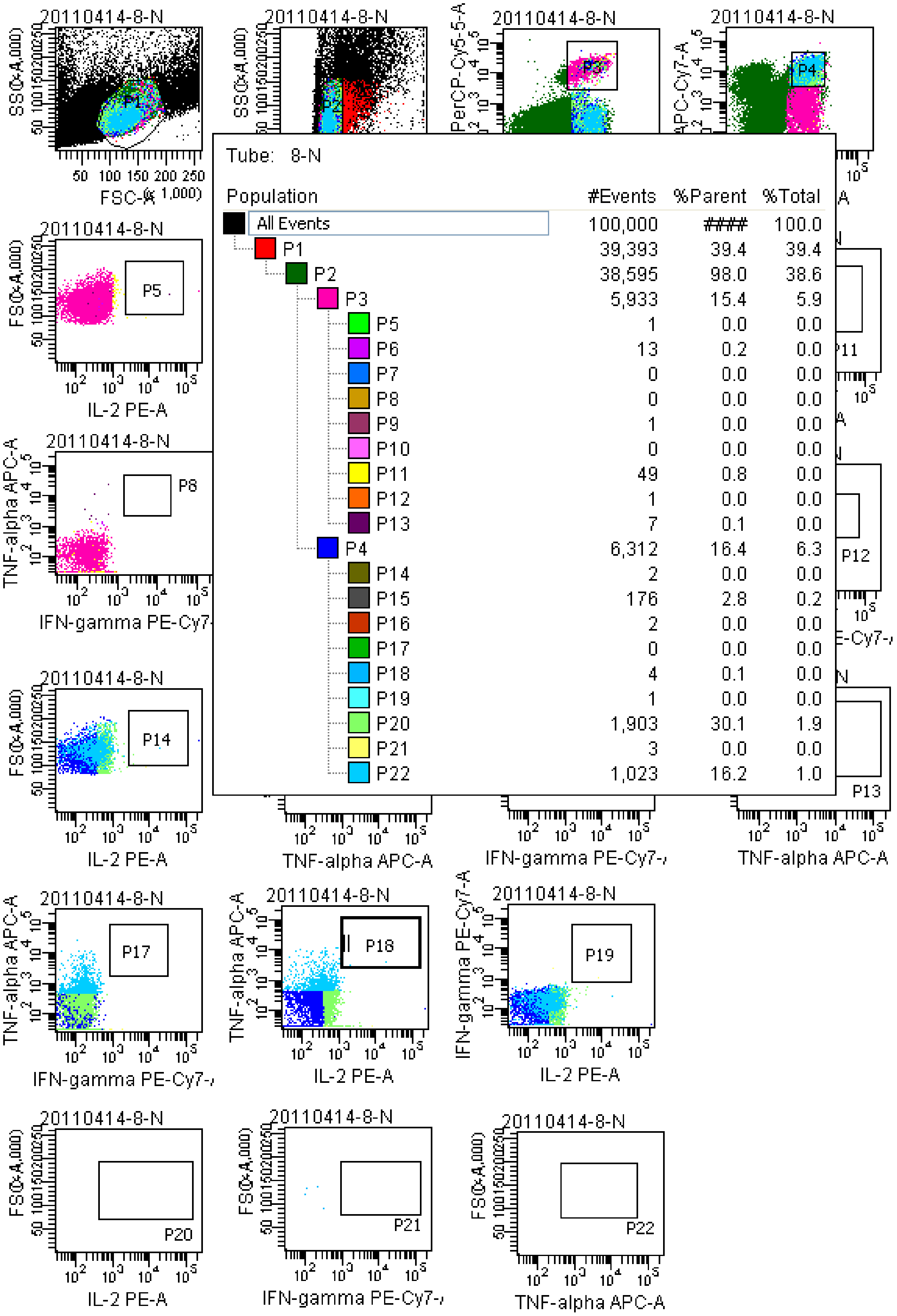

Tube: 8-N

Population	#Events	%Parent	%Total
All Events	100,000	####	100.0
P1	39,393	39.4	39.4
P2	38,595	98.0	38.6
P3	5,933	15.4	5.9
P5	1	0.0	0.0
P6	13	0.2	0.0
P7	0	0.0	0.0
P8	0	0.0	0.0
P9	1	0.0	0.0
P10	0	0.0	0.0
P11	49	0.8	0.0
P12	1	0.0	0.0
P13	7	0.1	0.0
P4	6,312	16.4	6.3
P14	2	0.0	0.0
P15	176	2.8	0.2
P16	2	0.0	0.0
P17	0	0.0	0.0
P18	4	0.1	0.0
P19	1	0.0	0.0
P20	1,903	30.1	1.9
P21	3	0.0	0.0
P22	1,023	16.2	1.0

284W-4vector:98R0053 (peptide)

FACSDiva Version 6.0

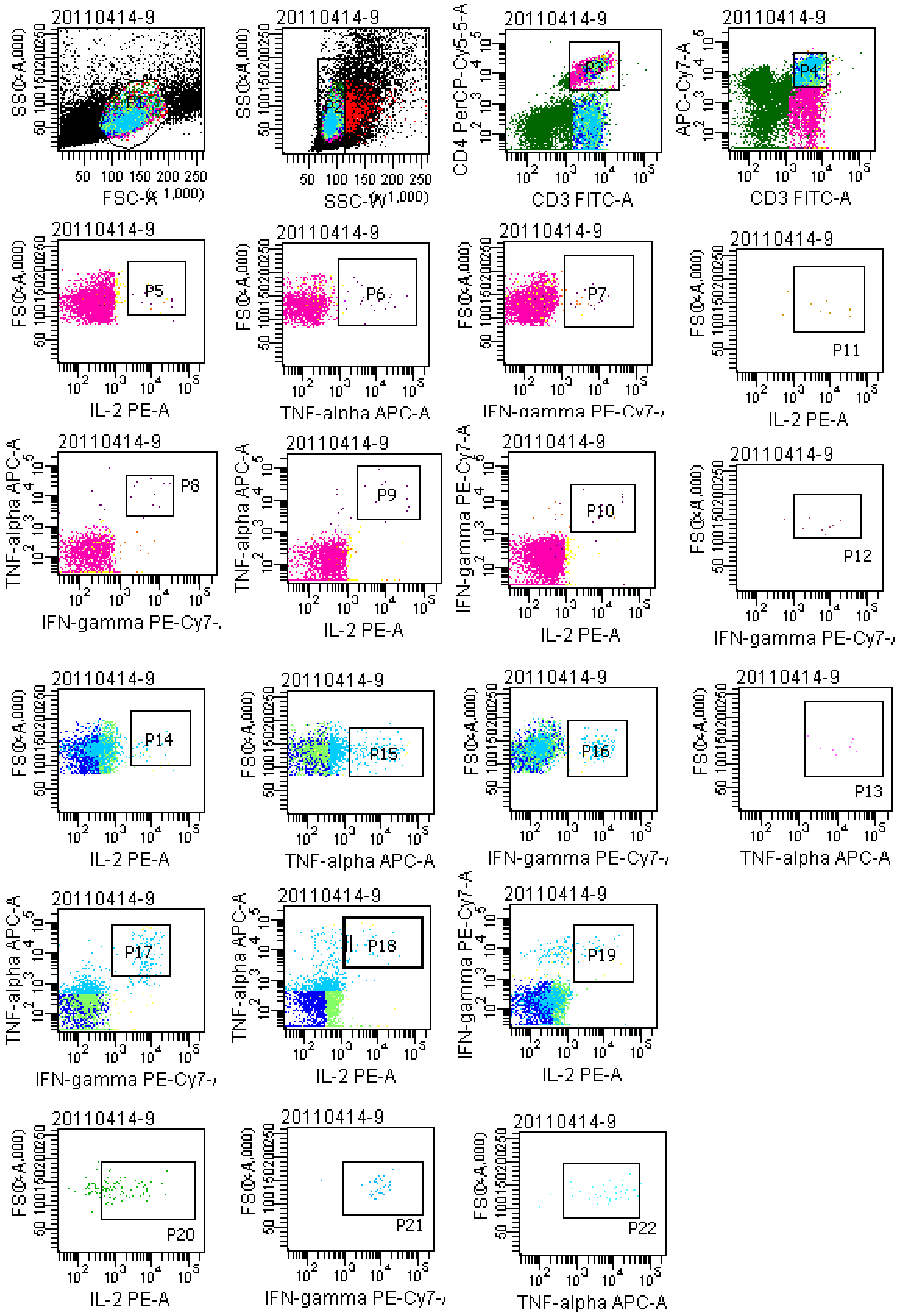

284W-4vector:98R0053 (peptide)

FACSDiva Version 6.0

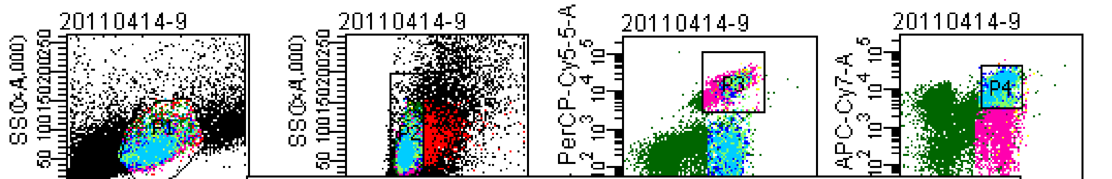

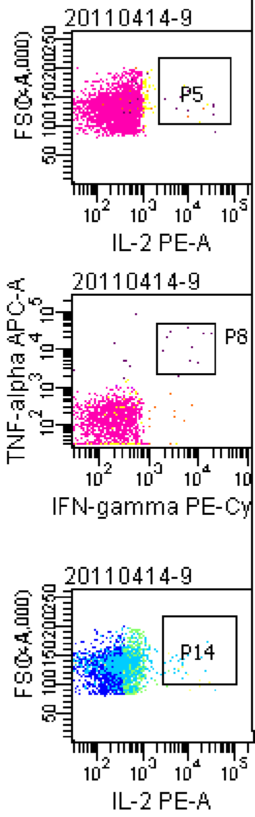

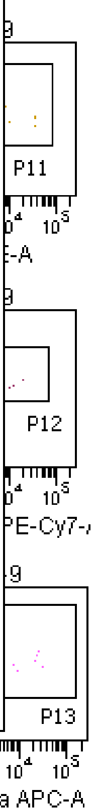

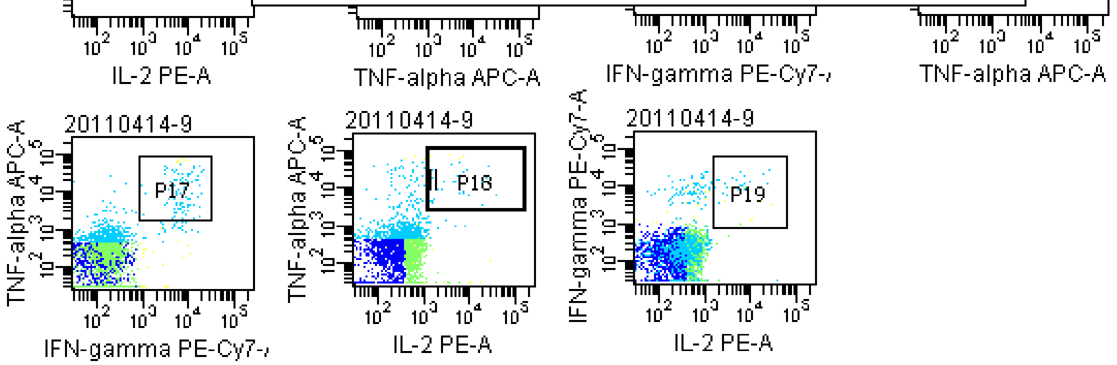

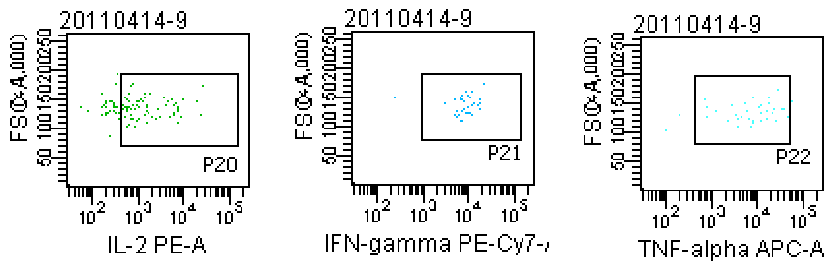

Tube: 9

Population	#Events	%Parent	%Total
All Events	100,000	####	100.0
P1	13,919	13.9	13.9
P2	13,273	95.4	13.3
P3	4,551	34.3	4.6
P5	14	0.3	0.0
P6	22	0.5	0.0
P7	18	0.4	0.0
P8	9	0.2	0.0
P9	9	0.2	0.0
P10	10	0.2	0.0
P11	67	1.5	0.1
P12	22	0.5	0.0
P13	18	0.4	0.0
P4	3,845	29.0	3.8
P14	30	0.8	0.0
P15	139	3.6	0.1
P16	142	3.7	0.1
P17	96	2.5	0.1
P18	34	0.9	0.0
P19	45	1.2	0.0
P20	1,347	35.0	1.3
P21	145	3.8	0.1
P22	691	18.0	0.7

284W-4vector:98R0053 (negative)

FACSDiva Version 6.0

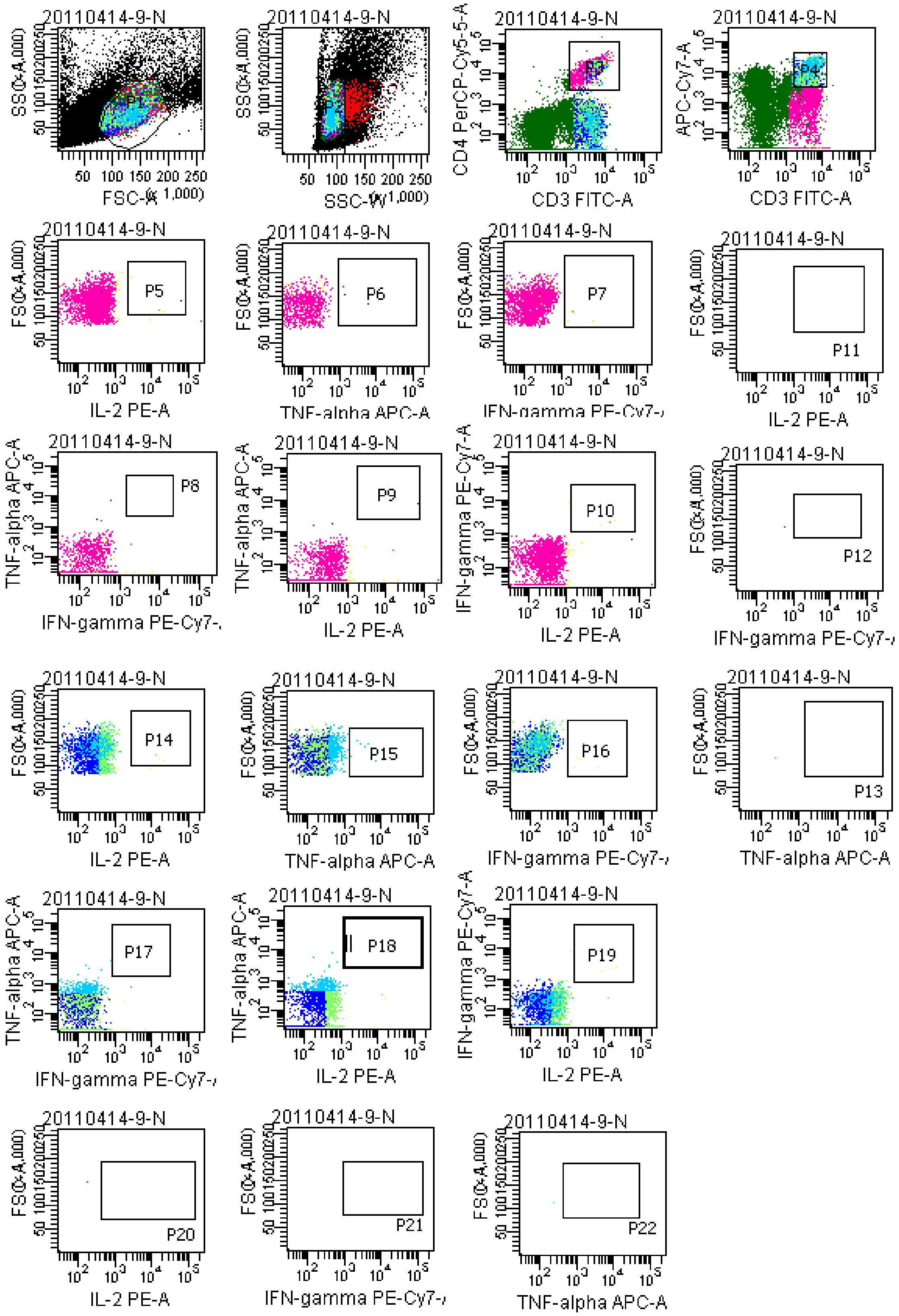

FACSDiva Version 6.0

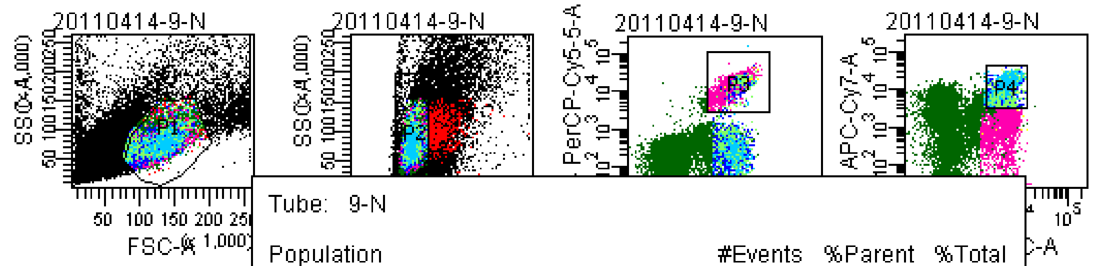

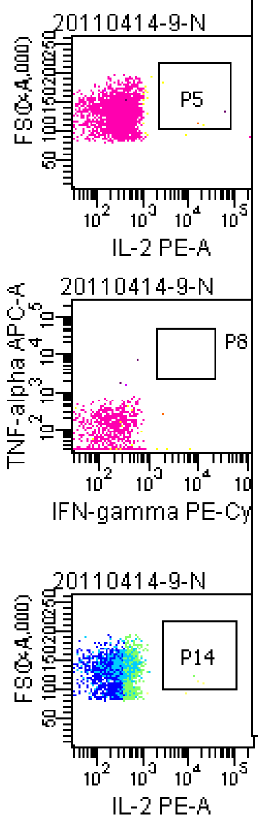

Tube: 9-N

Population	#Events	%Parent	%Total
All Events	100,000	####	100.0
P1	12,562	12.6	12.6
P2	12,010	95.6	12.0
P3	2,975	24.8	3.0
P5	4	0.1	0.0
P6	3	0.1	0.0
P7	4	0.1	0.0
P8	0	0.0	0.0
P9	1	0.0	0.0
P10	3	0.1	0.0
P11	19	0.6	0.0
P12	1	0.0	0.0
P13	2	0.1	0.0
P4	2,802	23.3	2.8
P14	3	0.1	0.0
P15	7	0.2	0.0
P16	5	0.2	0.0
P17	1	0.0	0.0
P18	0	0.0	0.0
P19	3	0.1	0.0
P20	841	30.0	0.8
P21	6	0.2	0.0
P22	373	13.3	0.4

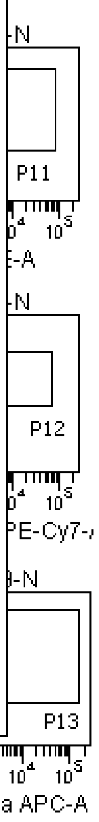

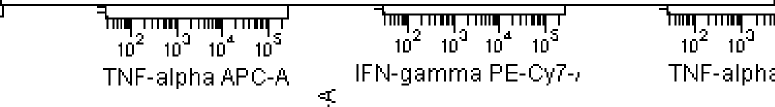

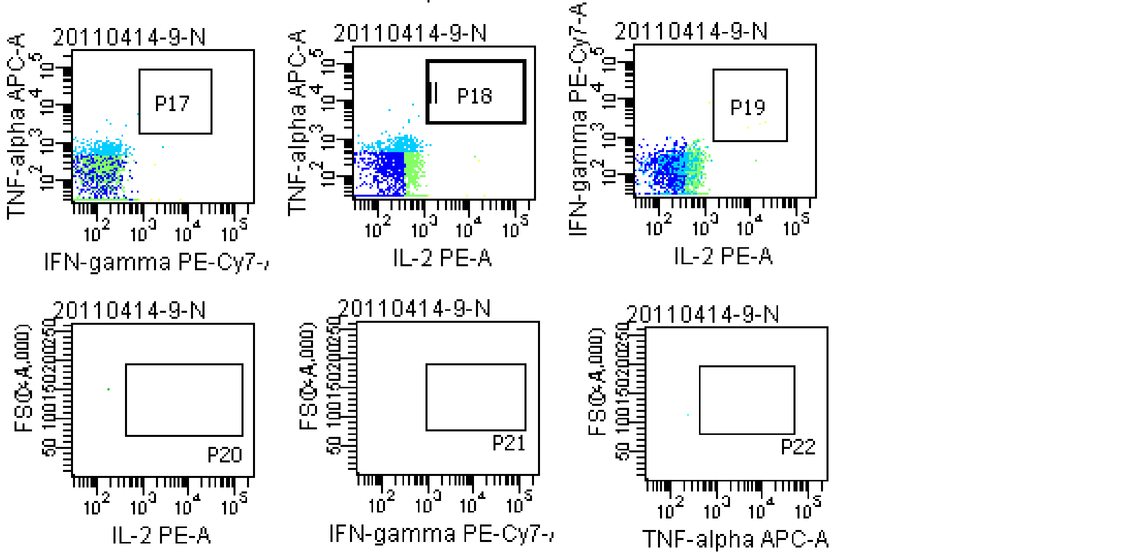

284W-4vector:00R0019 (peptide)

FACSDiva Version 6.0

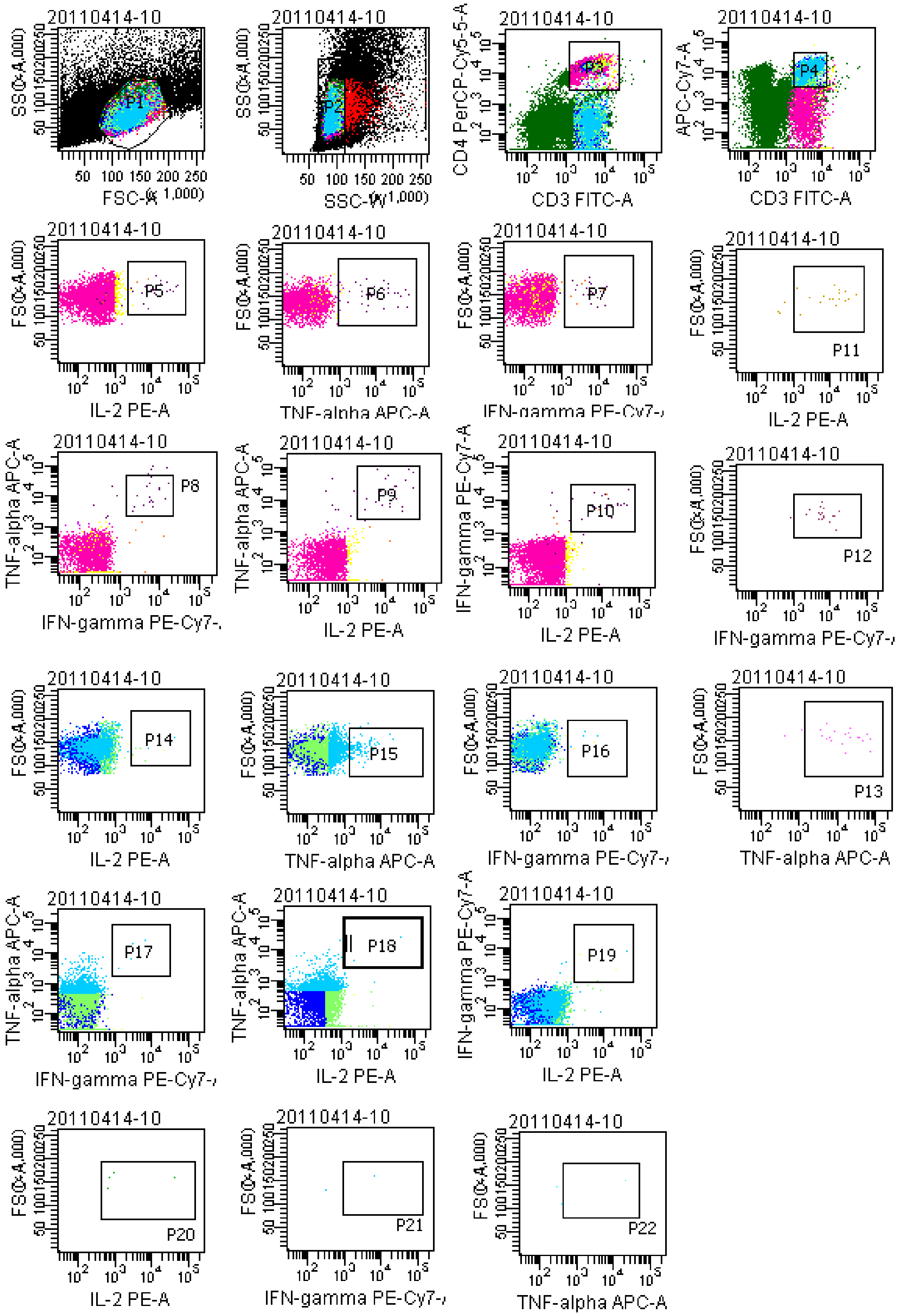

284W-4vector:00R0019 (peptide)

FACSDiva Version 6.0

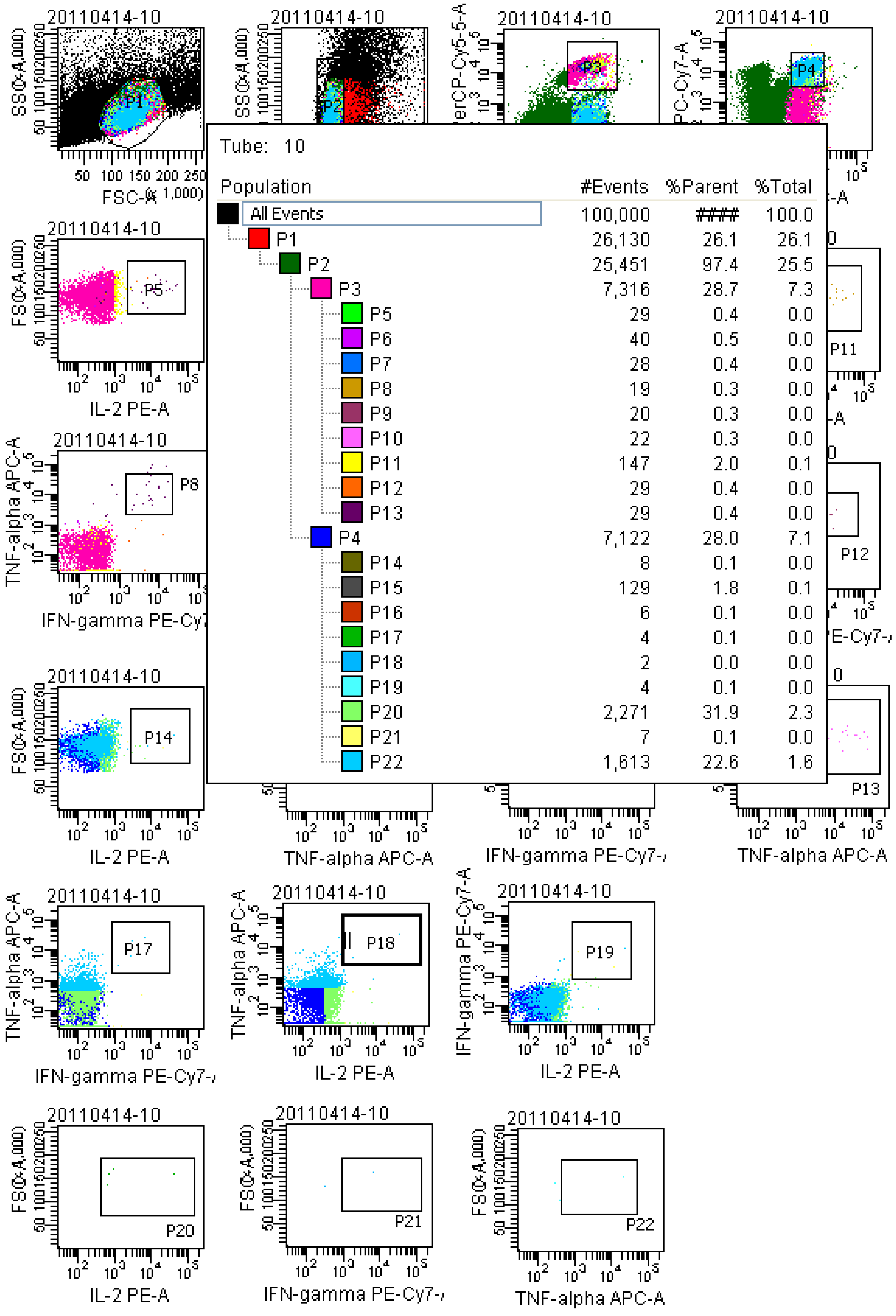

Tube: 10

Population	#Events	%Parent	%Total
All Events	100,000	####	100.0
P1	26,130	26.1	26.1
P2	25,451	97.4	25.5
P3	7,316	28.7	7.3
P5	29	0.4	0.0
P6	40	0.5	0.0
P7	28	0.4	0.0
P8	19	0.3	0.0
P9	20	0.3	0.0
P10	22	0.3	0.0
P11	147	2.0	0.1
P12	29	0.4	0.0
P13	29	0.4	0.0
P4	7,122	28.0	7.1
P14	8	0.1	0.0
P15	129	1.8	0.1
P16	6	0.1	0.0
P17	4	0.1	0.0
P18	2	0.0	0.0
P19	4	0.1	0.0
P20	2,271	31.9	2.3
P21	7	0.1	0.0
P22	1,613	22.6	1.6

284W-4vector:00R0019 (negative)

FACSDiva Version 6.0

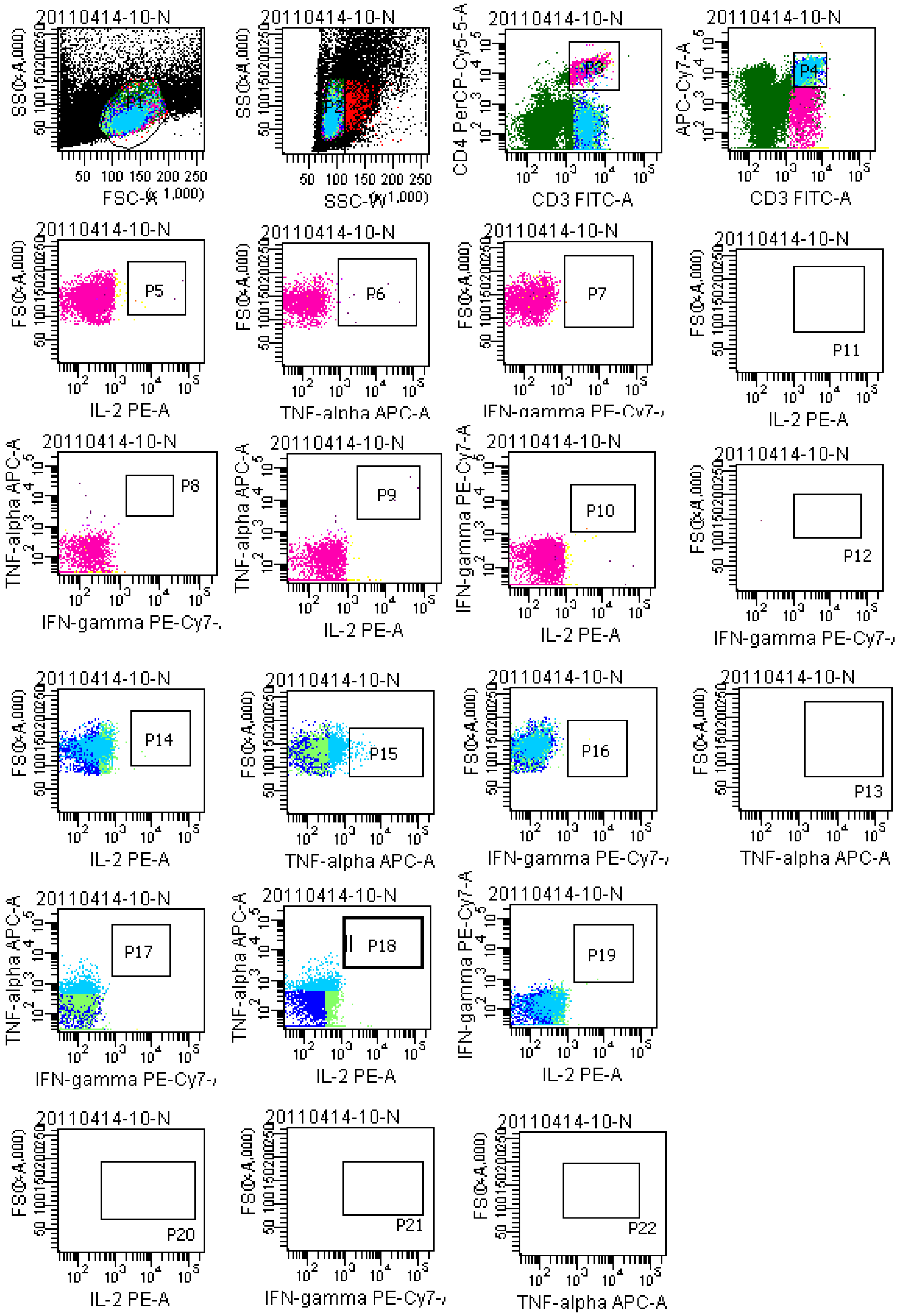

284W-4vector:00R0019 (negative)

FACSDiva Version 6.0

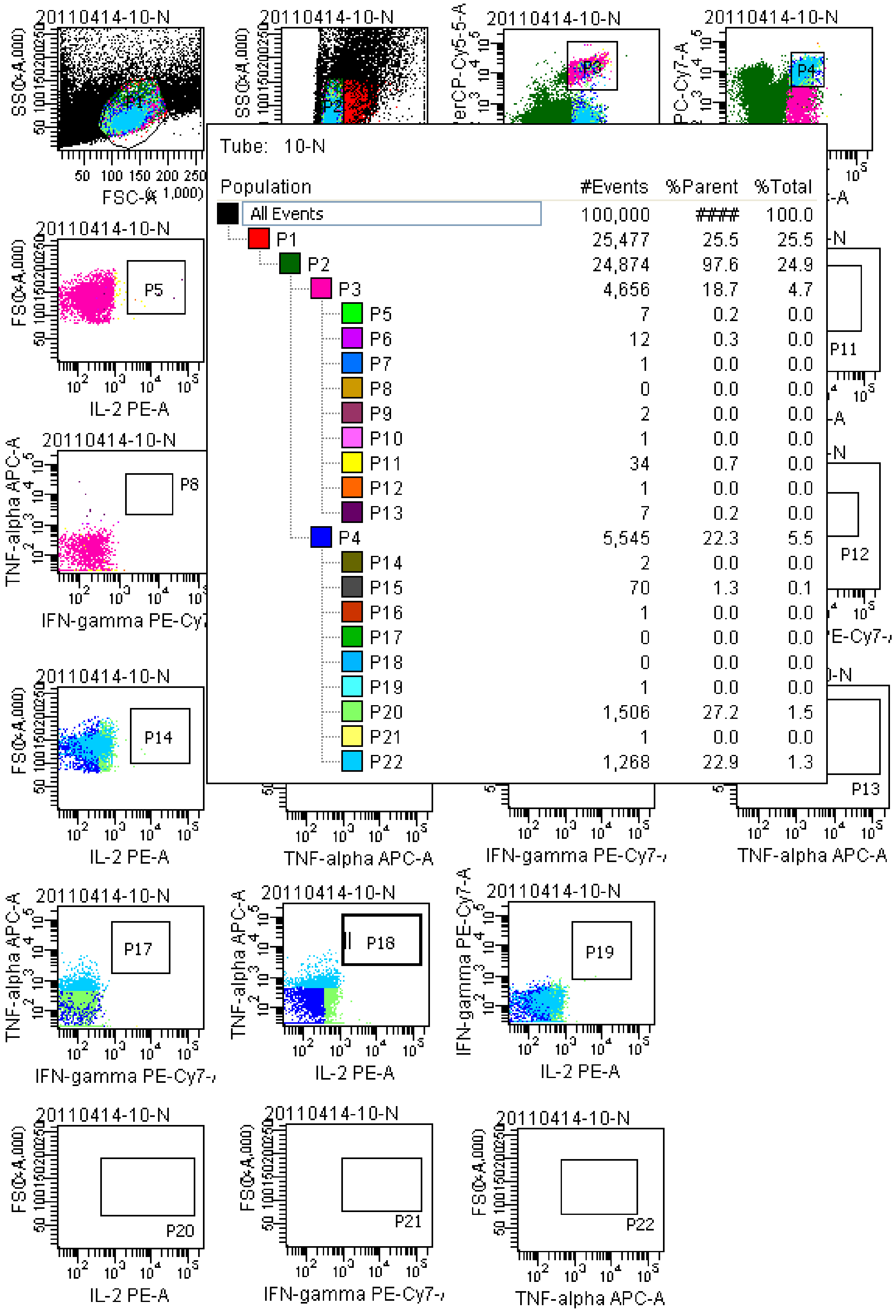

Tube: 10-N

Population	#Events	%Parent	%Total
All Events	100,000	####	100.0
P1	25,477	25.5	25.5
P2	24,874	97.6	24.9
P3	4,656	18.7	4.7
P5	7	0.2	0.0
P6	12	0.3	0.0
P7	1	0.0	0.0
P8	0	0.0	0.0
P9	2	0.0	0.0
P10	1	0.0	0.0
P11	34	0.7	0.0
P12	1	0.0	0.0
P13	7	0.2	0.0
P4	5,545	22.3	5.5
P14	2	0.0	0.0
P15	70	1.3	0.1
P16	1	0.0	0.0
P17	0	0.0	0.0
P18	0	0.0	0.0
P19	1	0.0	0.0
P20	1,506	27.2	1.5
P21	1	0.0	0.0
P22	1,268	22.9	1.3

284W-2vector:98R0037 (peptide)

FACSDiva Version 6.0

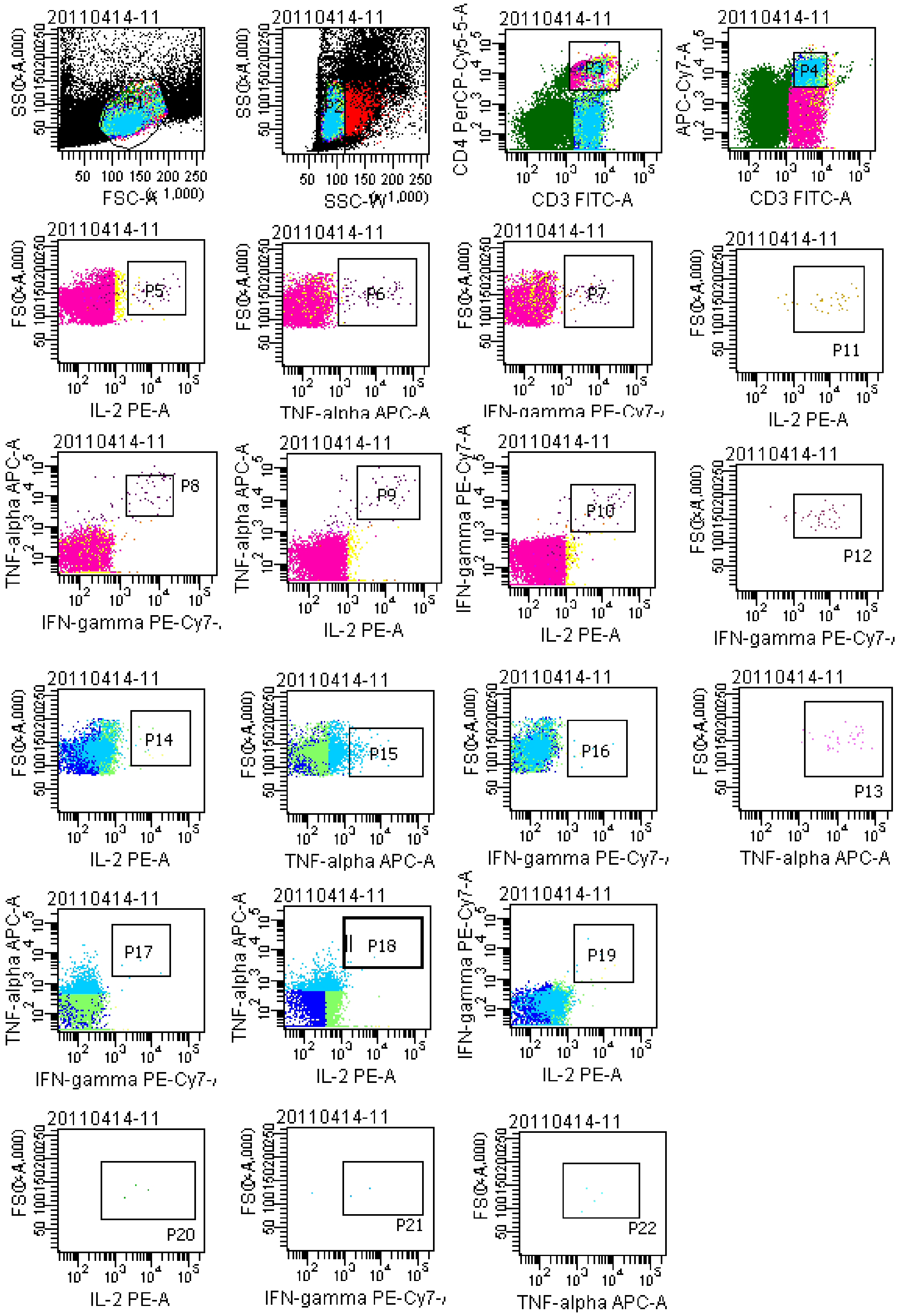

FACSDiva Version 6.0

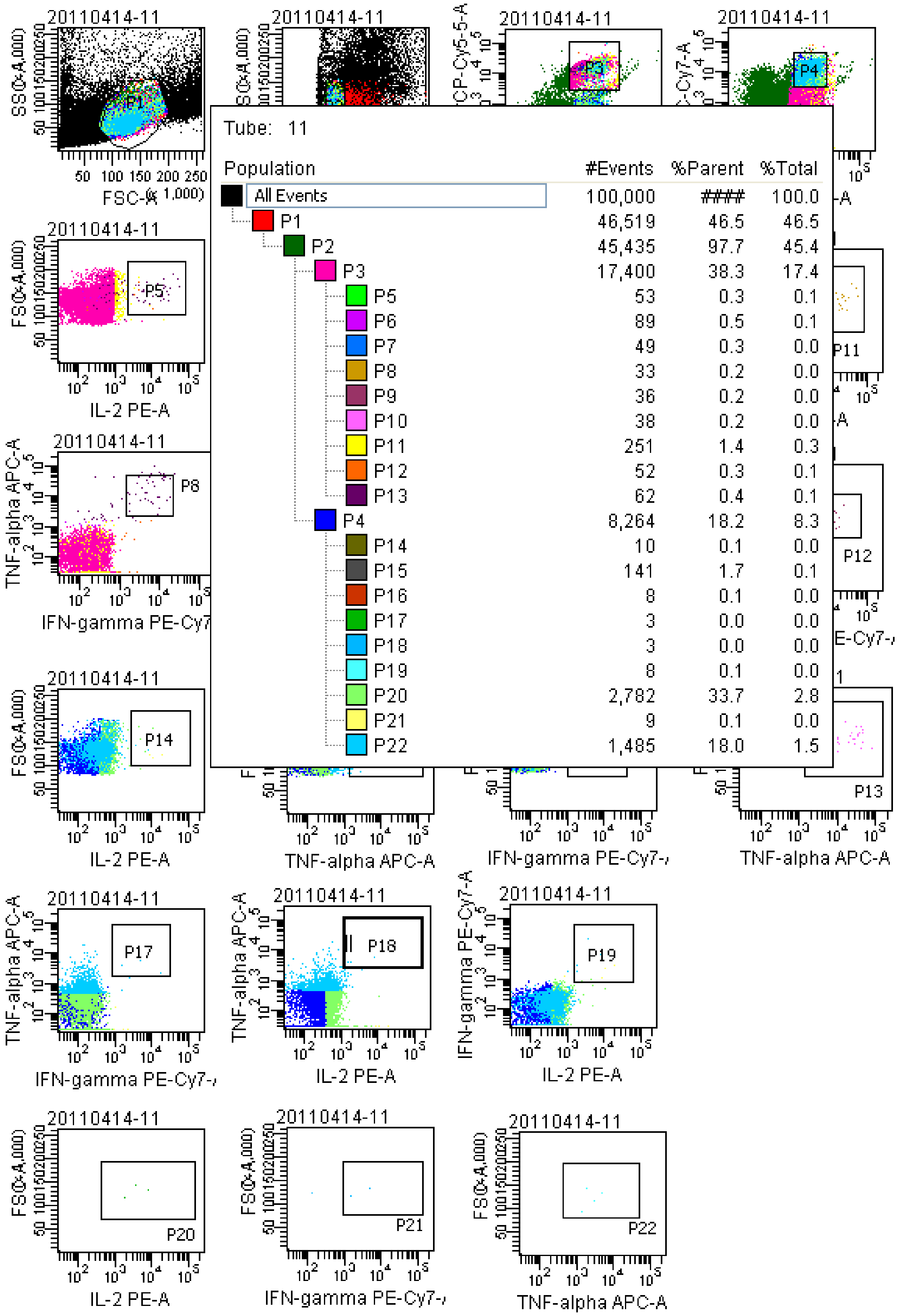

Tube: 11

Population	#Events	%Parent	%Total
All Events	100,000	####	100.0
P1	46,519	46.5	46.5
P2	45,435	97.7	45.4
P3	17,400	38.3	17.4
P5	53	0.3	0.1
P6	89	0.5	0.1
P7	49	0.3	0.0
P8	33	0.2	0.0
P9	36	0.2	0.0
P10	38	0.2	0.0
P11	251	1.4	0.3
P12	52	0.3	0.1
P13	62	0.4	0.1
P4	8,264	18.2	8.3
P14	10	0.1	0.0
P15	141	1.7	0.1
P16	8	0.1	0.0
P17	3	0.0	0.0
P18	3	0.0	0.0
P19	8	0.1	0.0
P20	2,782	33.7	2.8
P21	9	0.1	0.0
P22	1,485	18.0	1.5

284W-2vector:98R0037 (negative)

FACSDiva Version 6.0

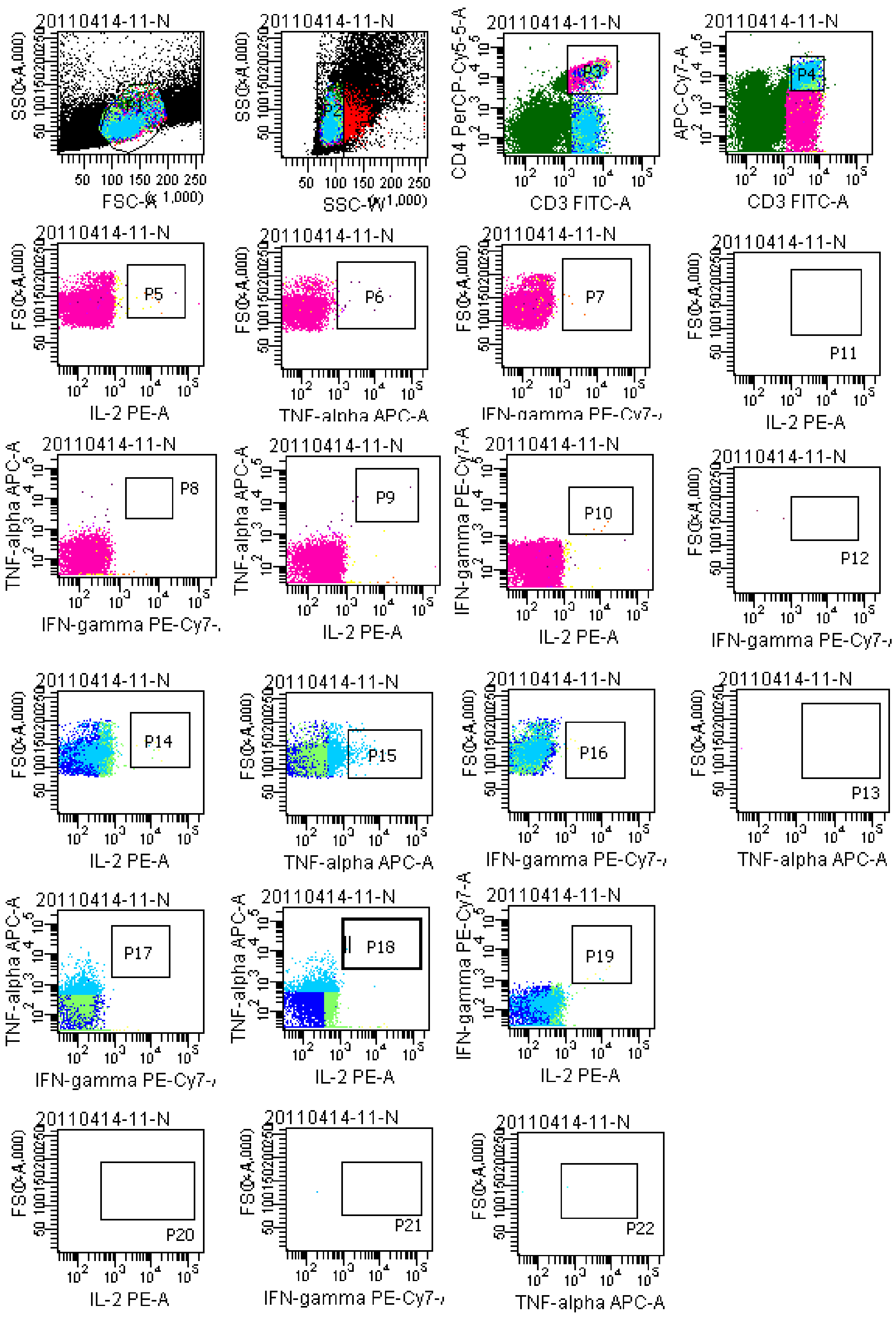

FACSDiva Version 6.0

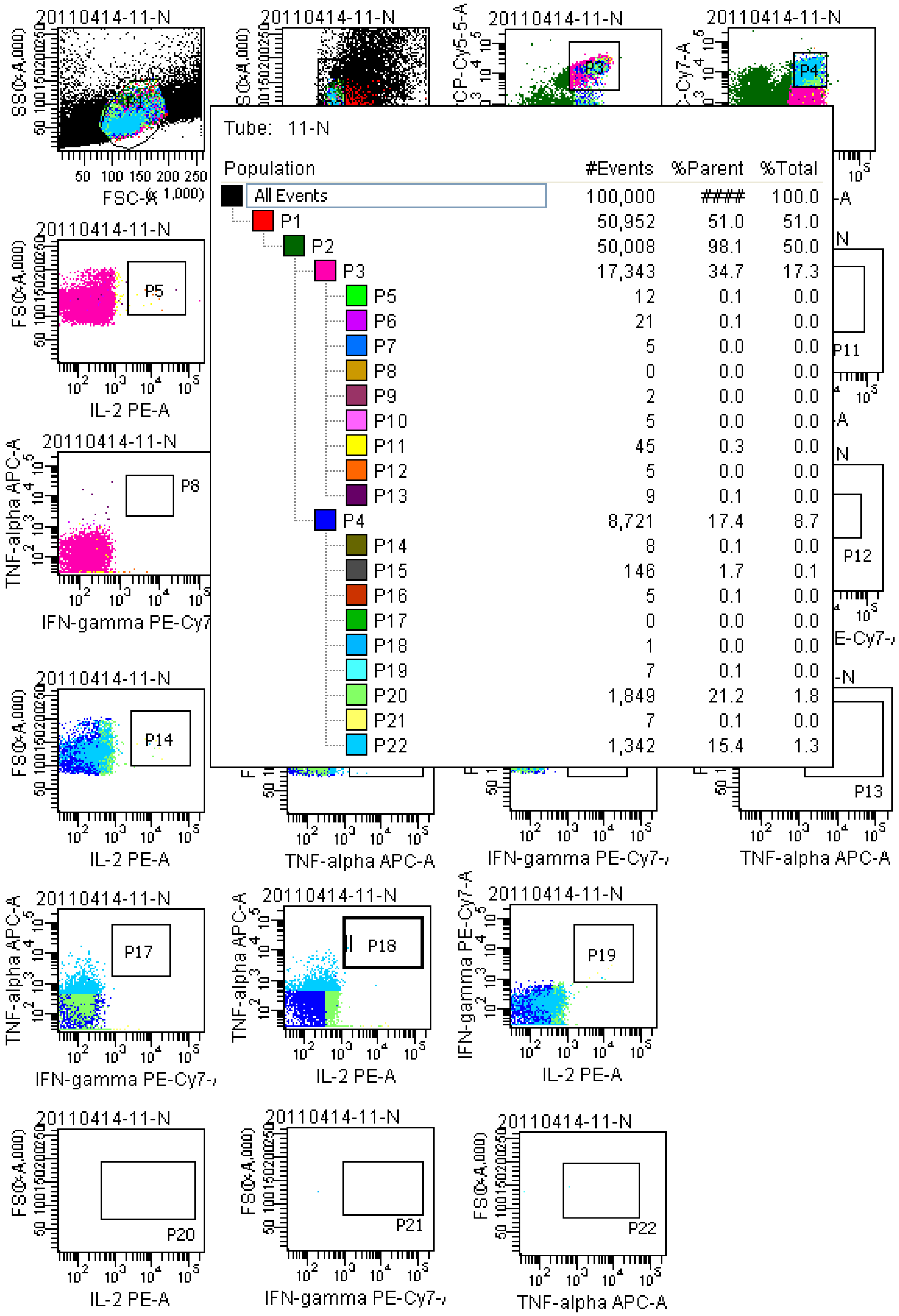

Tube: 11-N

Population	#Events	%Parent	%Total
All Events	100,000	####	100.0
P1	50,952	51.0	51.0
P2	50,008	98.1	50.0
P3	17,343	34.7	17.3
P5	12	0.1	0.0
P6	21	0.1	0.0
P7	5	0.0	0.0
P8	0	0.0	0.0
P9	2	0.0	0.0
P10	5	0.0	0.0
P11	45	0.3	0.0
P12	5	0.0	0.0
P13	9	0.1	0.0
P4	8,721	17.4	8.7
P14	8	0.1	0.0
P15	146	1.7	0.1
P16	5	0.1	0.0
P17	0	0.0	0.0
P18	1	0.0	0.0
P19	7	0.1	0.0
P20	1,849	21.2	1.8
P21	7	0.1	0.0
P22	1,342	15.4	1.3

284W-4vector:99R0035 (peptide)

FACSDiva Version 6.0

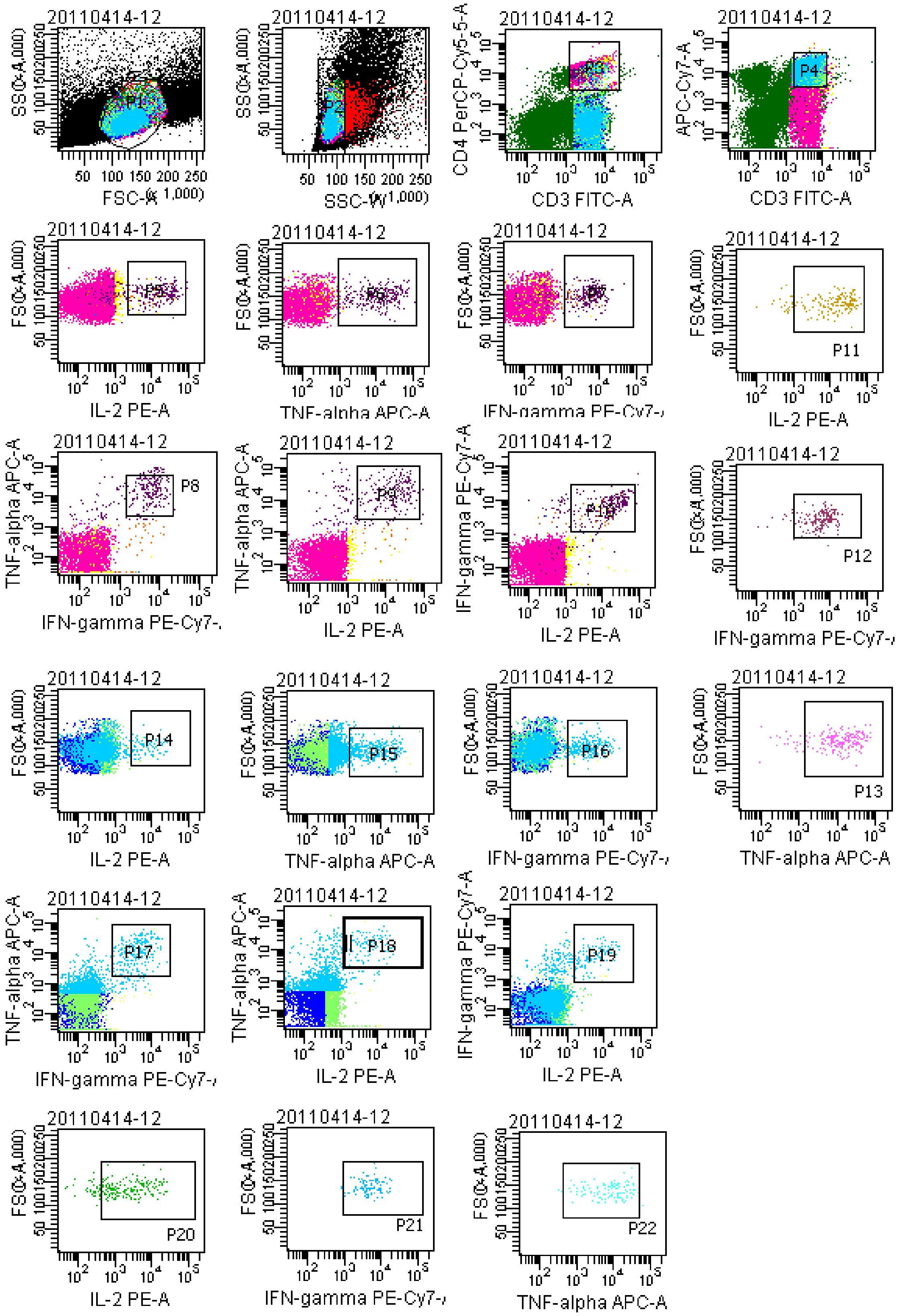

FACSDiva Version 6.0

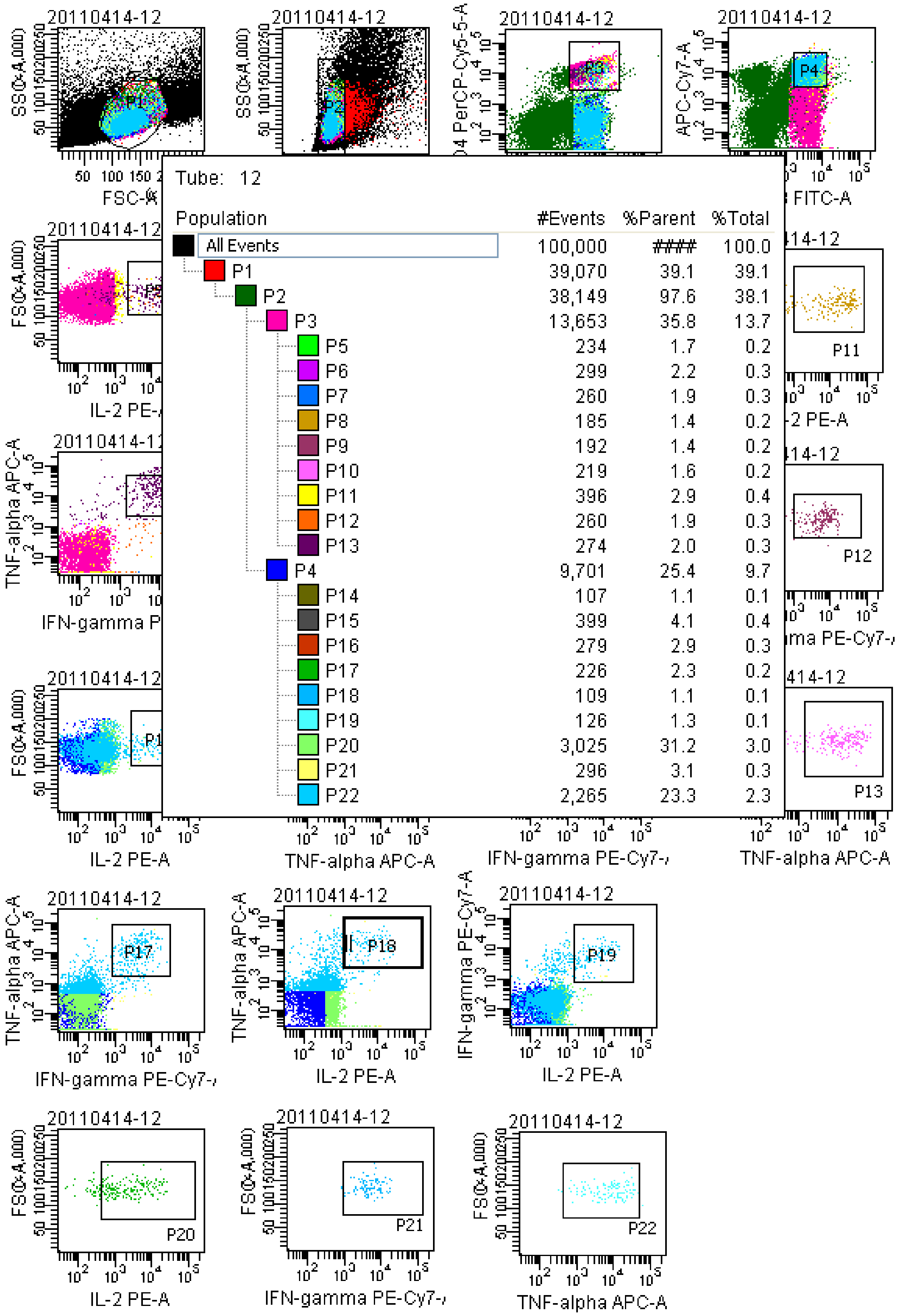

Tube: 12

Population	#Events	%Parent	%Total
All Events	100,000	####	100.0
P1	39,070	39.1	39.1
P2	38,149	97.6	38.1
P3	13,653	35.8	13.7
P5	234	1.7	0.2
P6	299	2.2	0.3
P7	260	1.9	0.3
P8	185	1.4	0.2
P9	192	1.4	0.2
P10	219	1.6	0.2
P11	396	2.9	0.4
P12	260	1.9	0.3
P13	274	2.0	0.3
P4	9,701	25.4	9.7
P14	107	1.1	0.1
P15	399	4.1	0.4
P16	279	2.9	0.3
P17	226	2.3	0.2
P18	109	1.1	0.1
P19	126	1.3	0.1
P20	3,025	31.2	3.0
P21	296	3.1	0.3
P22	2,265	23.3	2.3

284W-4vector:99R0035 (negative)

FACSDiva Version 6.0

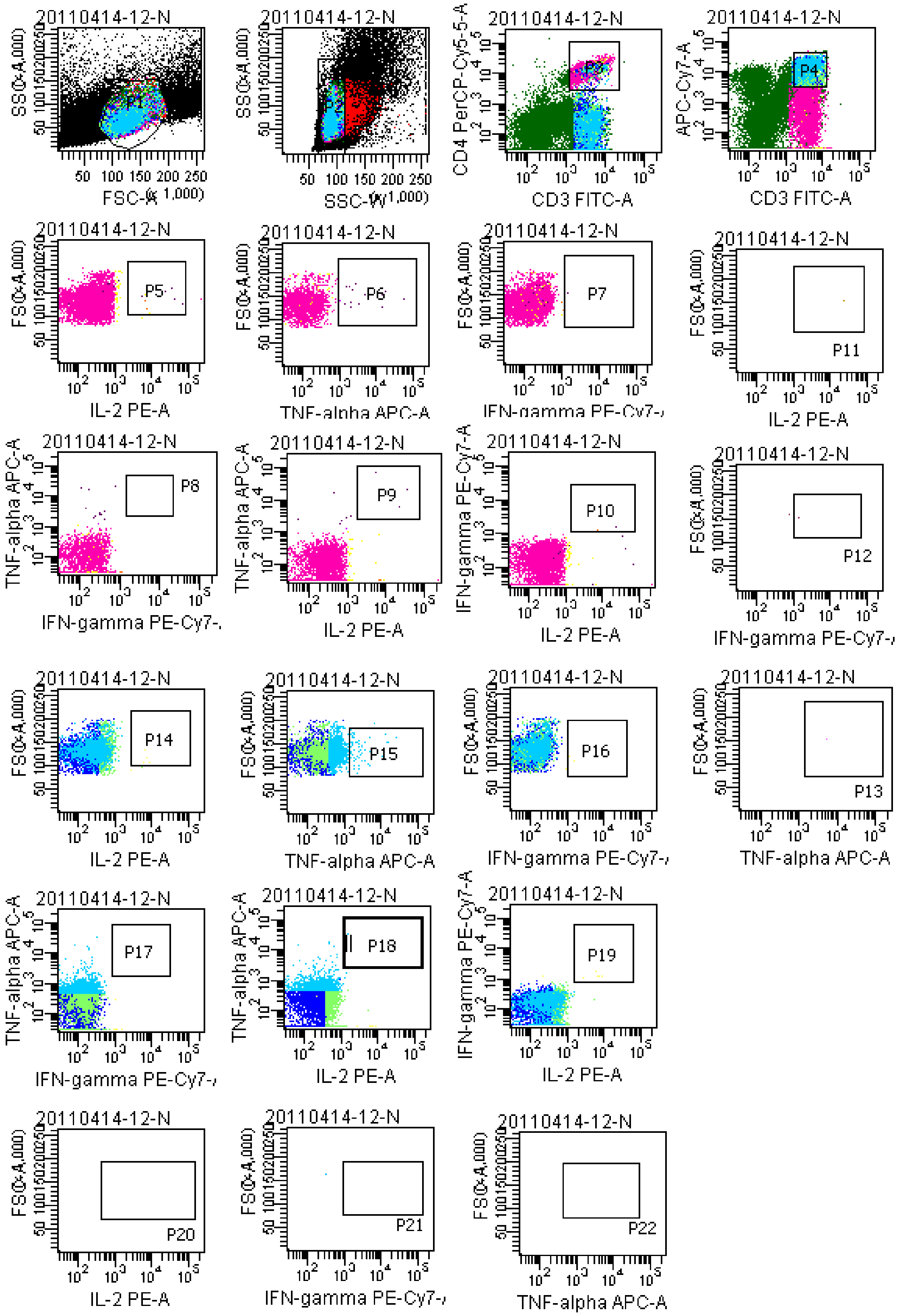

FACSDiva Version 6.0

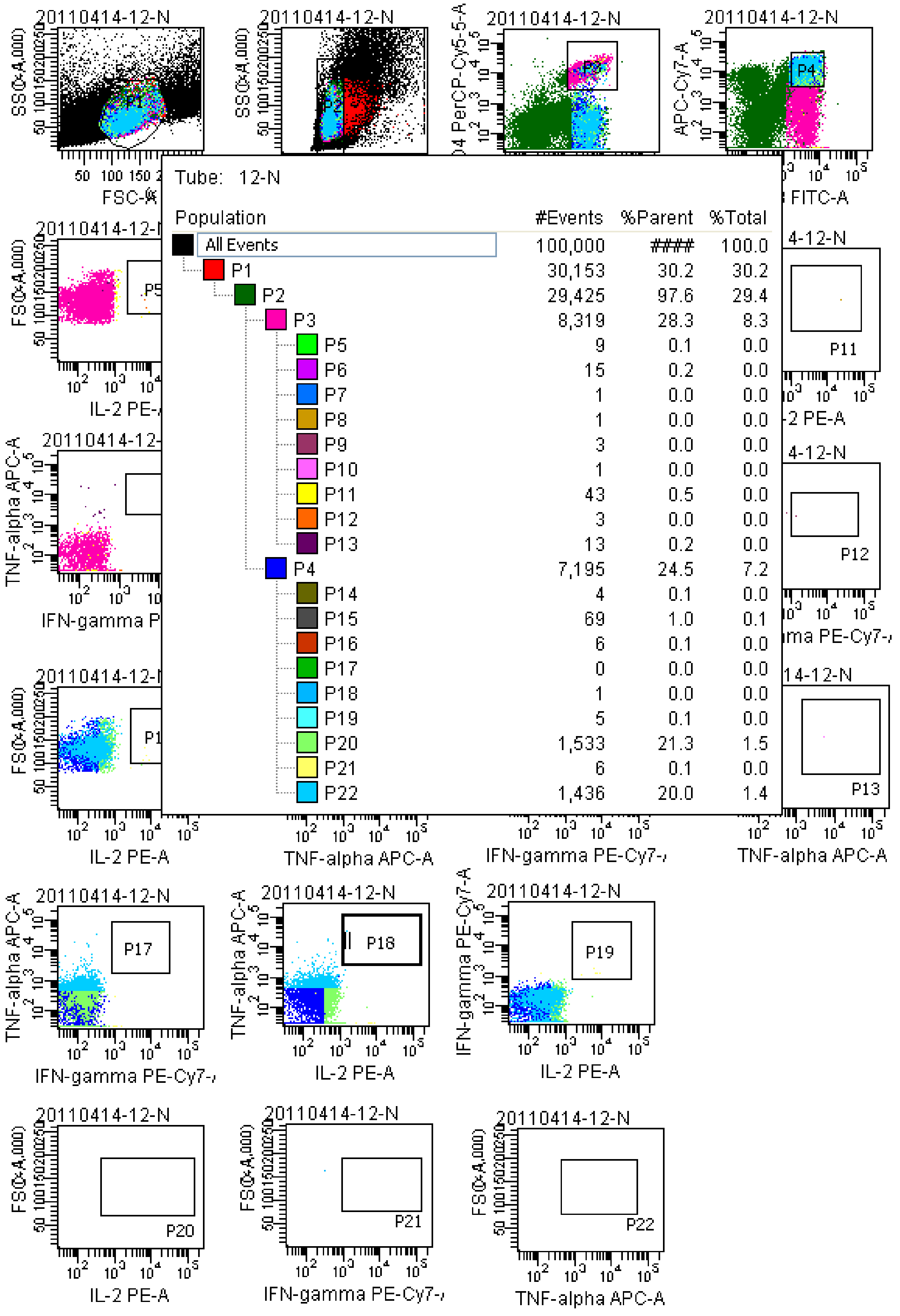

Tube: 12-N

Population	#Events	%Parent	%Total
All Events	100,000	####	100.0
P1	30,153	30.2	30.2
P2	29,425	97.6	29.4
P3	8,319	28.3	8.3
P5	9	0.1	0.0
P6	15	0.2	0.0
P7	1	0.0	0.0
P8	1	0.0	0.0
P9	3	0.0	0.0
P10	1	0.0	0.0
P11	43	0.5	0.0
P12	3	0.0	0.0
P13	13	0.2	0.0
P4	7,195	24.5	7.2
P14	4	0.1	0.0
P15	69	1.0	0.1
P16	6	0.1	0.0
P17	0	0.0	0.0
P18	1	0.0	0.0
P19	5	0.1	0.0
P20	1,533	21.3	1.5
P21	6	0.1	0.0
P22	1,436	20.0	1.4

FACSDiva Version 6.0

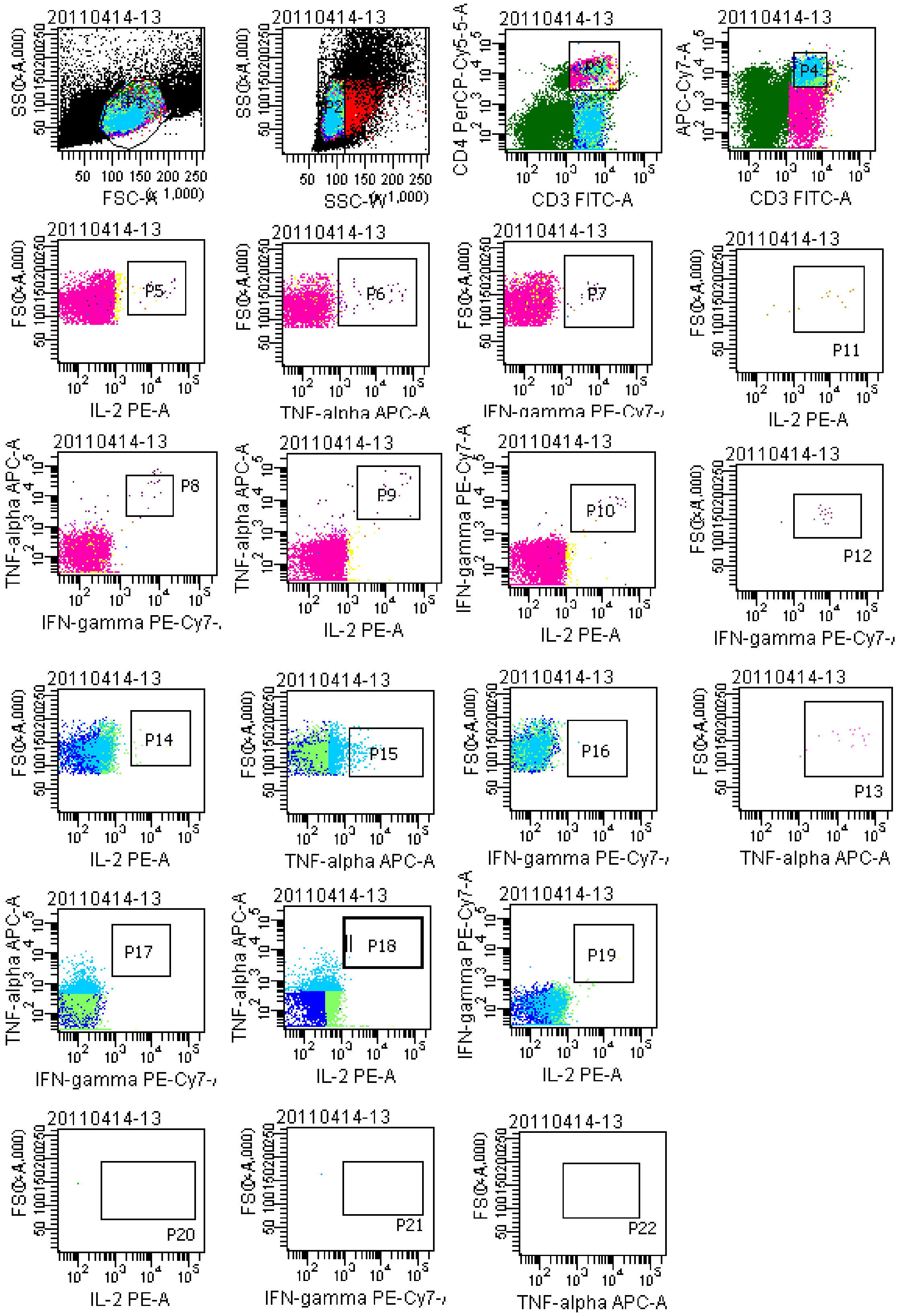

284W-4vector:98R0013 (peptide)

FACSDiva Version 6.0

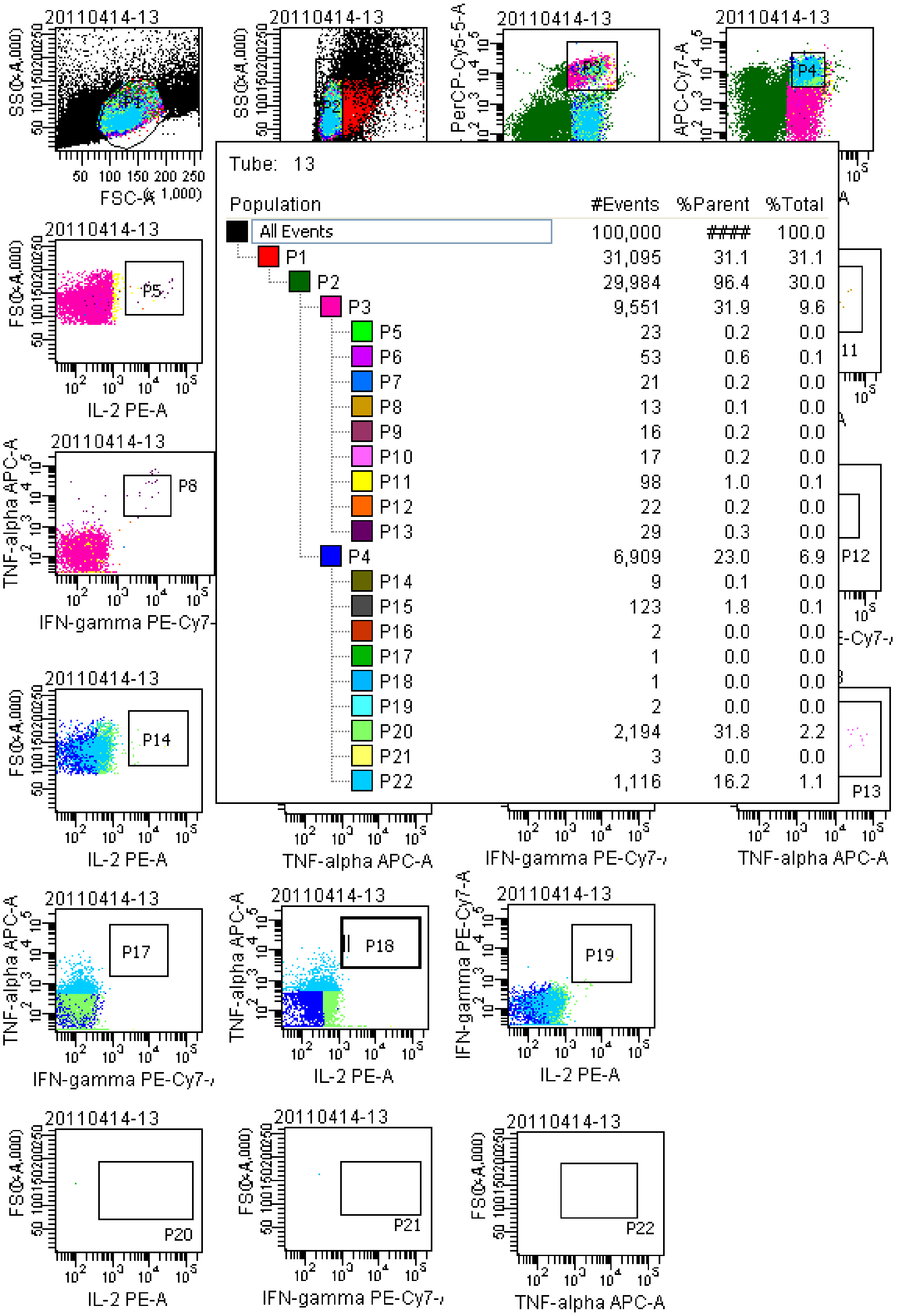

Tube: 13

Population	#Events	%Parent	%Total
All Events	100,000	####	100.0
P1	31,095	31.1	31.1
P2	29,984	96.4	30.0
P3	9,551	31.9	9.6
P5	23	0.2	0.0
P6	53	0.6	0.1
P7	21	0.2	0.0
P8	13	0.1	0.0
P9	16	0.2	0.0
P10	17	0.2	0.0
P11	98	1.0	0.1
P12	22	0.2	0.0
P13	29	0.3	0.0
P4	6,909	23.0	6.9
P14	9	0.1	0.0
P15	123	1.8	0.1
P16	2	0.0	0.0
P17	1	0.0	0.0
P18	1	0.0	0.0
P19	2	0.0	0.0
P20	2,194	31.8	2.2
P21	3	0.0	0.0
P22	1,116	16.2	1.1

284W-4vector:98R0013 (negative)

FACSDiva Version 6.0

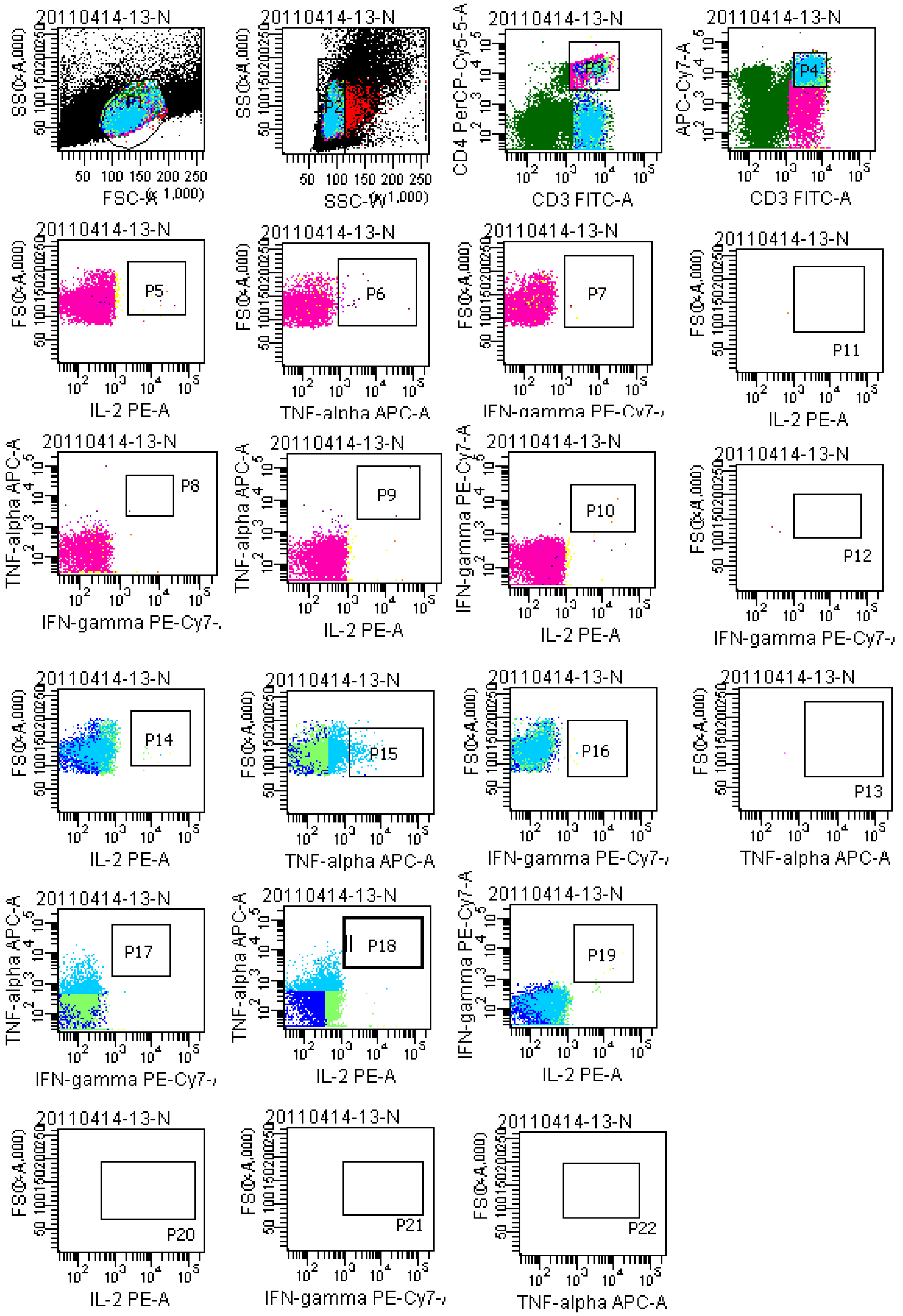

284W-4vector:98R0013 (negative)

FACSDiva Version 6.0

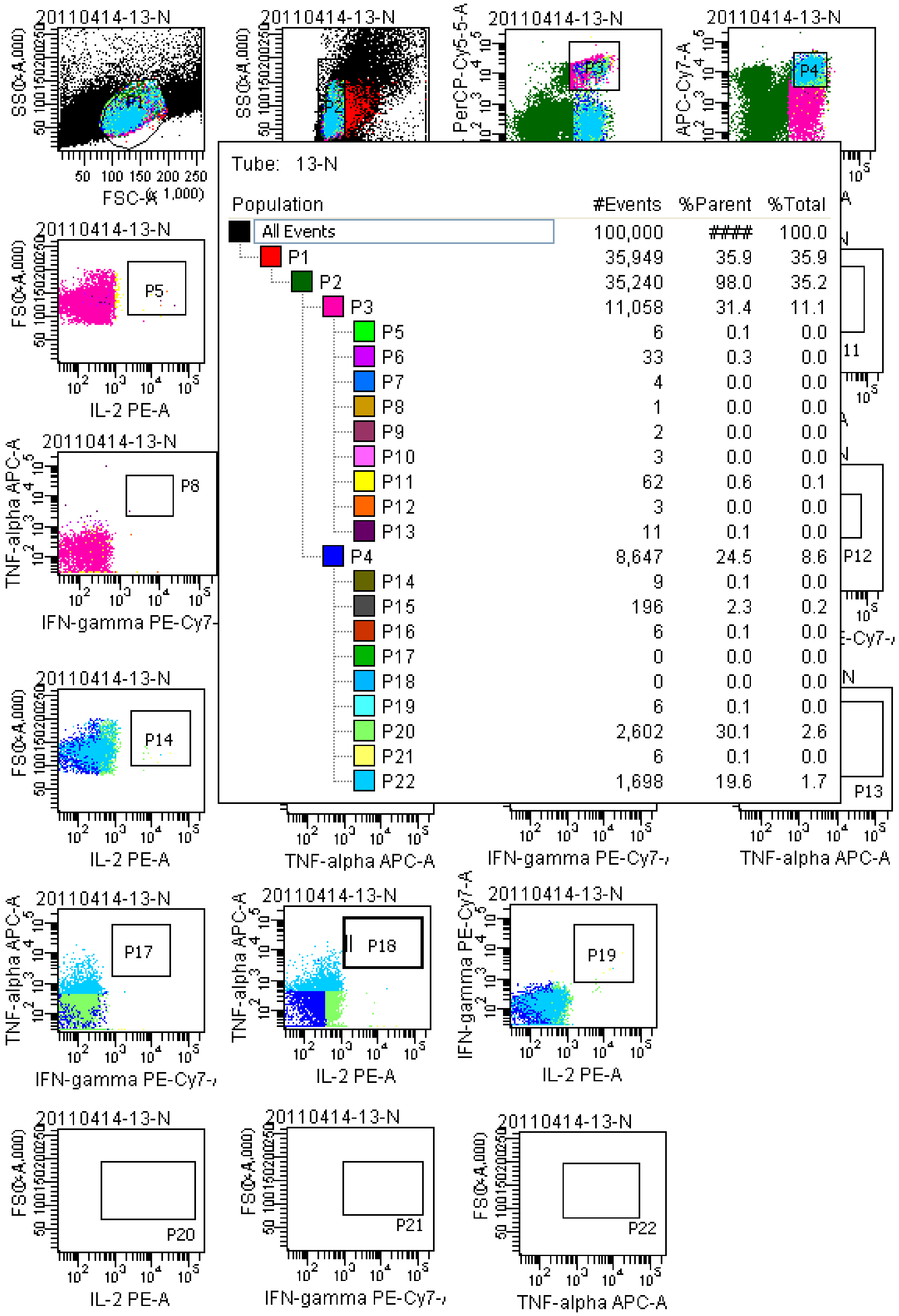

Tube: 13-N

Population	#Events	%Parent	%Total
All Events	100,000	####	100.0
P1	35,949	35.9	35.9
P2	35,240	98.0	35.2
P3	11,058	31.4	11.1
P5	6	0.1	0.0
P6	33	0.3	0.0
P7	4	0.0	0.0
P8	1	0.0	0.0
P9	2	0.0	0.0
P10	3	0.0	0.0
P11	62	0.6	0.1
P12	3	0.0	0.0
P13	11	0.1	0.0
P4	8,647	24.5	8.6
P14	9	0.1	0.0
P15	196	2.3	0.2
P16	6	0.1	0.0
P17	0	0.0	0.0
P18	0	0.0	0.0
P19	6	0.1	0.0
P20	2,602	30.1	2.6
P21	6	0.1	0.0
P22	1,698	19.6	1.7

284W-control:98R0009 (peptide)

FACSDiva Version 6.0

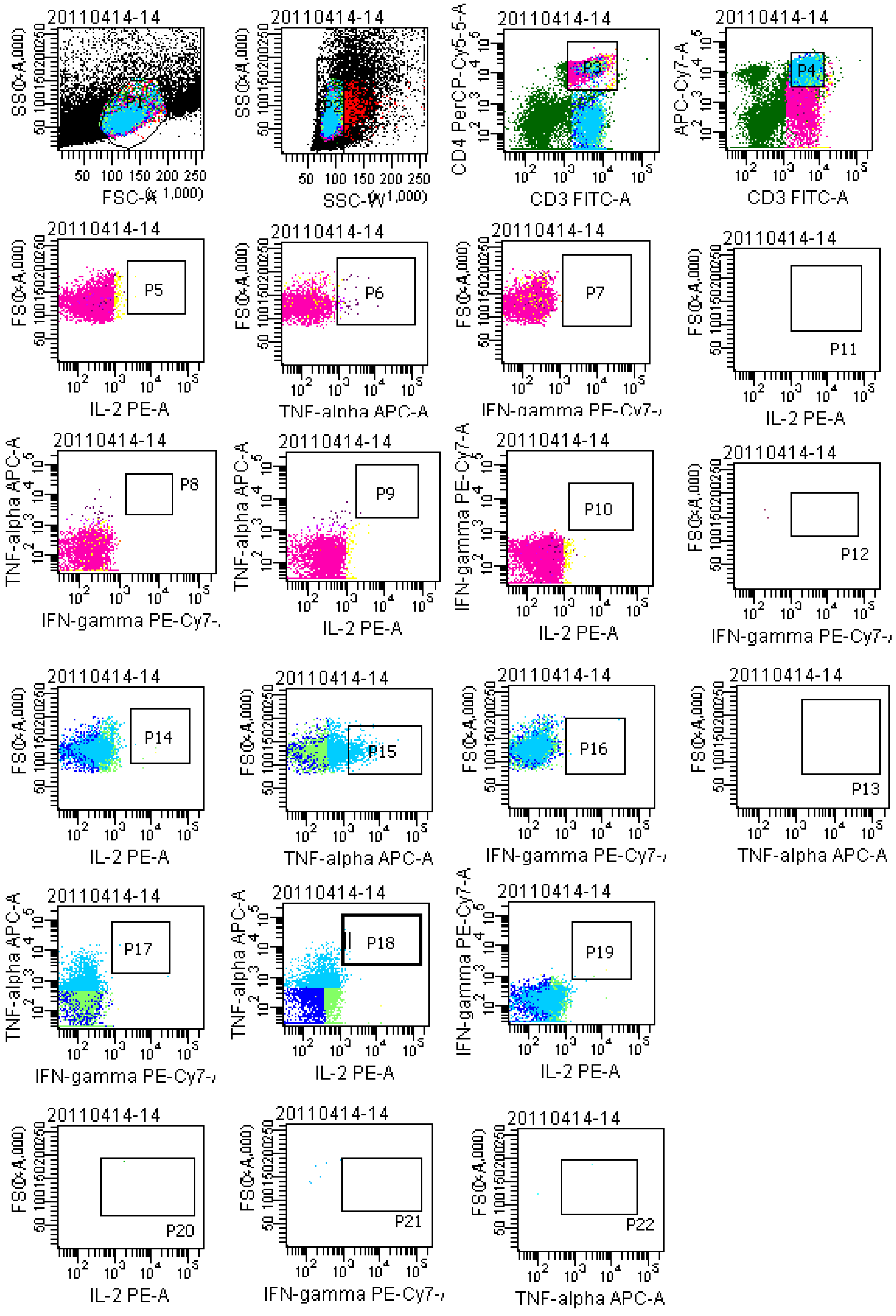

284W-control:98R0009 (peptide)

FACSDiva Version 6.0

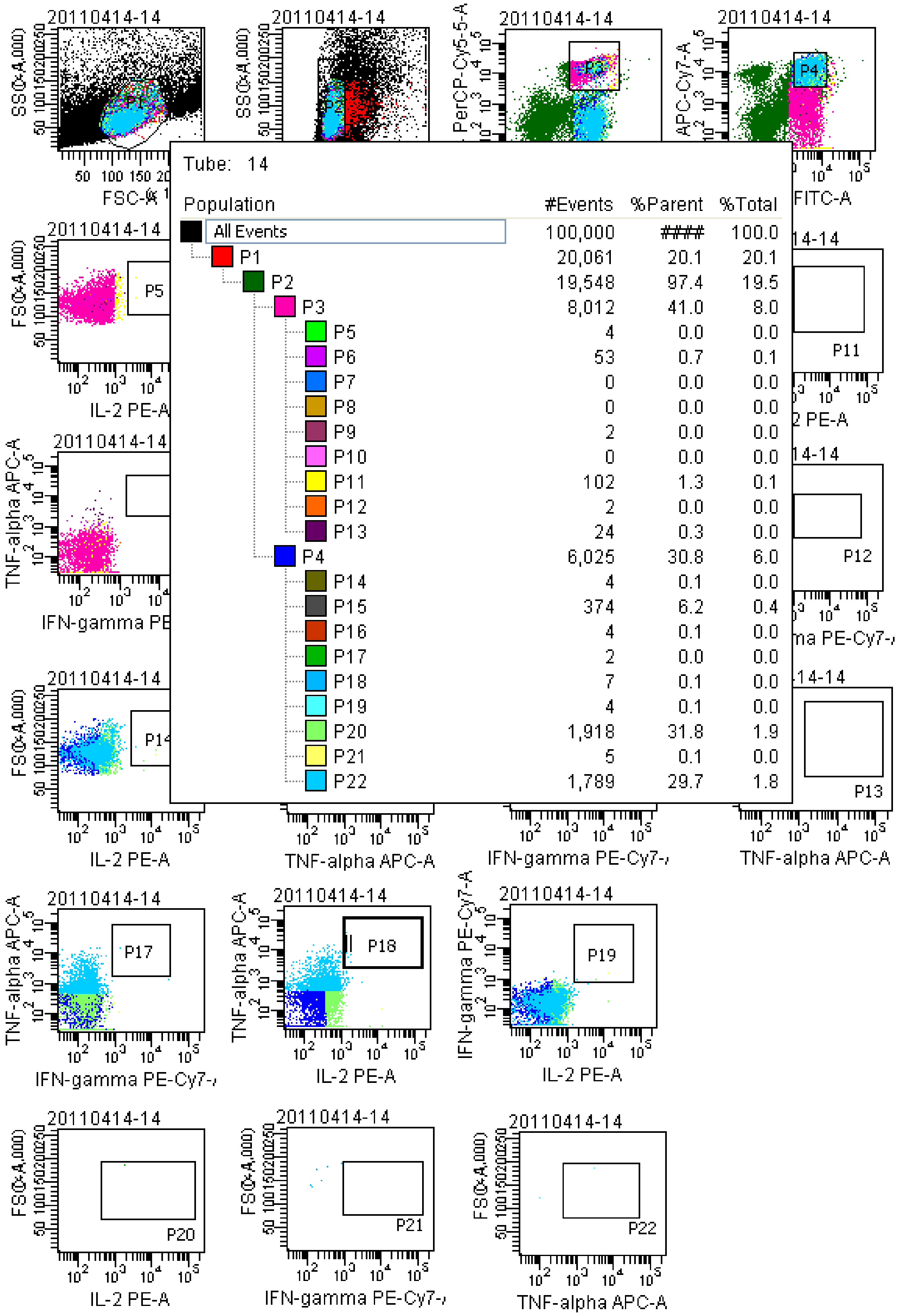

Tube: 14

Population	#Events	%Parent	%Total
All Events	100,000	####	100.0
P1	20,061	20.1	20.1
P2	19,548	97.4	19.5
P3	8,012	41.0	8.0
P5	4	0.0	0.0
P6	53	0.7	0.1
P7	0	0.0	0.0
P8	0	0.0	0.0
P9	2	0.0	0.0
P10	0	0.0	0.0
P11	102	1.3	0.1
P12	2	0.0	0.0
P13	24	0.3	0.0
P4	6,025	30.8	6.0
P14	4	0.1	0.0
P15	374	6.2	0.4
P16	4	0.1	0.0
P17	2	0.0	0.0
P18	7	0.1	0.0
P19	4	0.1	0.0
P20	1,918	31.8	1.9
P21	5	0.1	0.0
P22	1,789	29.7	1.8

284W-control:98R0009 (negative)

FACSDiva Version 6.0

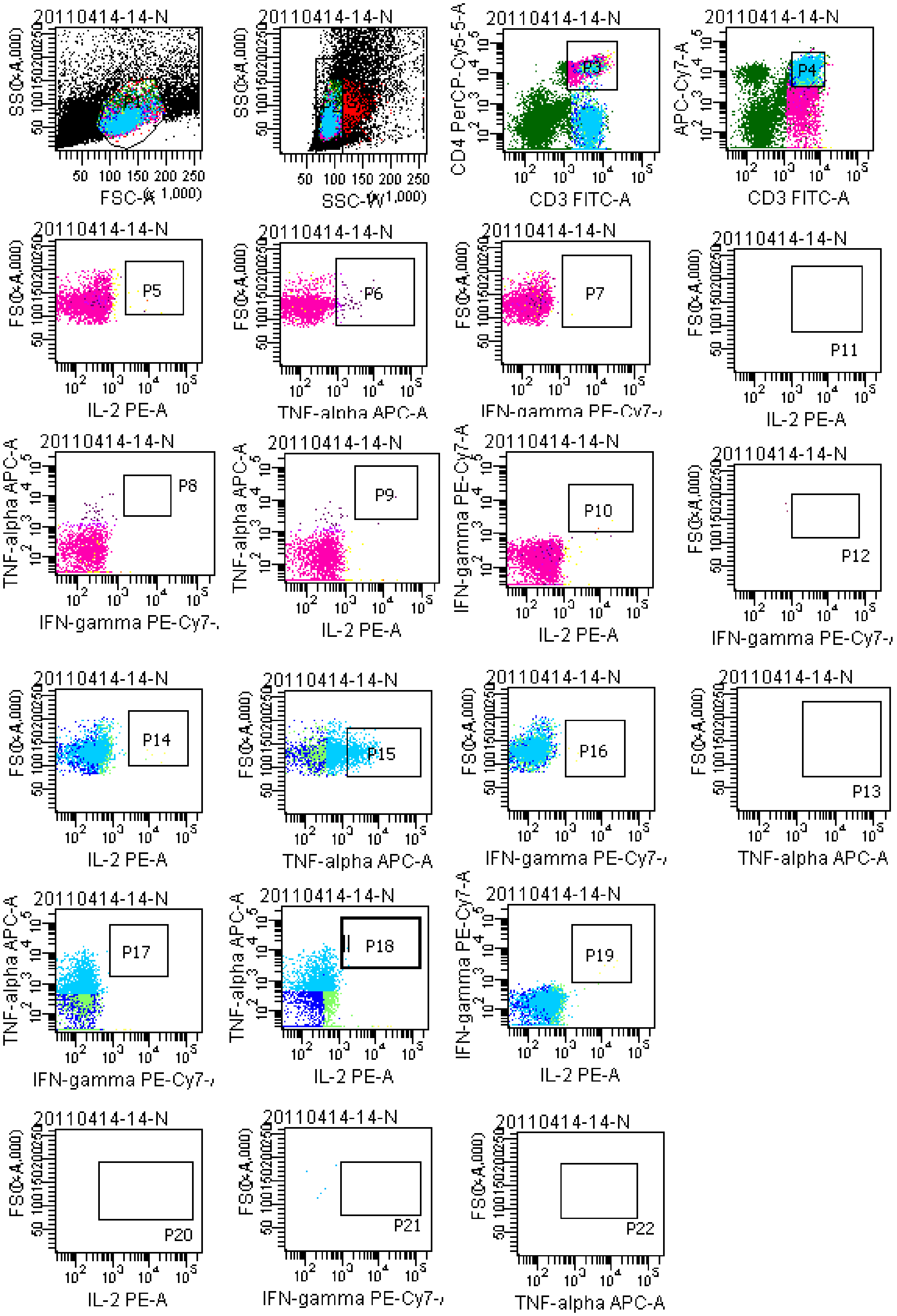

284W-control:98R0009 (negative)

FACSDiva Version 6.0

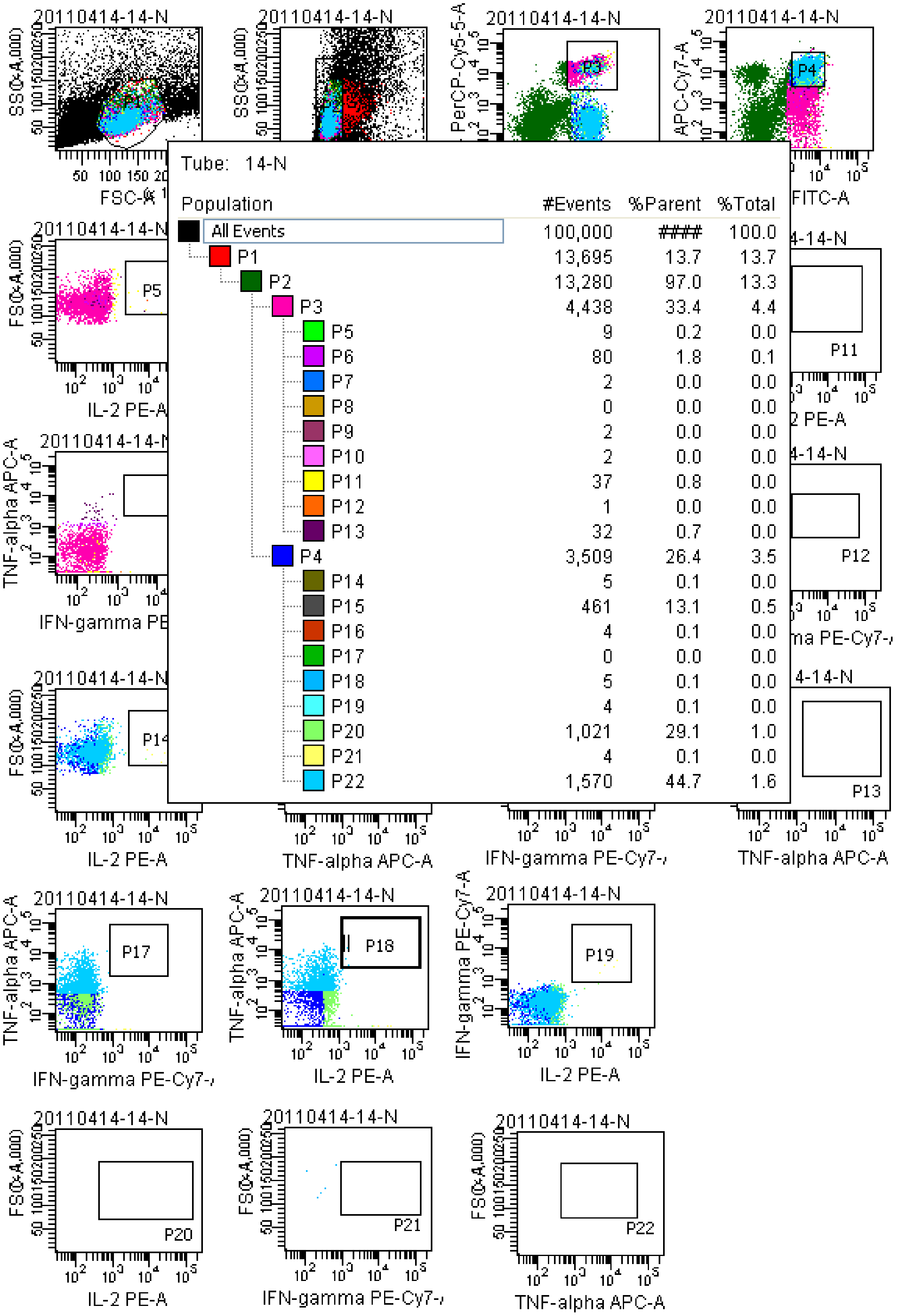

Tube: 14-N

Population	#Events	%Parent	%Total
All Events	100,000	####	100.0
P1	13,695	13.7	13.7
P2	13,280	97.0	13.3
P3	4,438	33.4	4.4
P5	9	0.2	0.0
P6	80	1.8	0.1
P7	2	0.0	0.0
P8	0	0.0	0.0
P9	2	0.0	0.0
P10	2	0.0	0.0
P11	37	0.8	0.0
P12	1	0.0	0.0
P13	32	0.7	0.0
P4	3,509	26.4	3.5
P14	5	0.1	0.0
P15	461	13.1	0.5
P16	4	0.1	0.0
P17	0	0.0	0.0
P18	5	0.1	0.0
P19	4	0.1	0.0
P20	1,021	29.1	1.0
P21	4	0.1	0.0
P22	1,570	44.7	1.6

284W-control:00R0023 (peptide)

FACSDiva Version 6.0

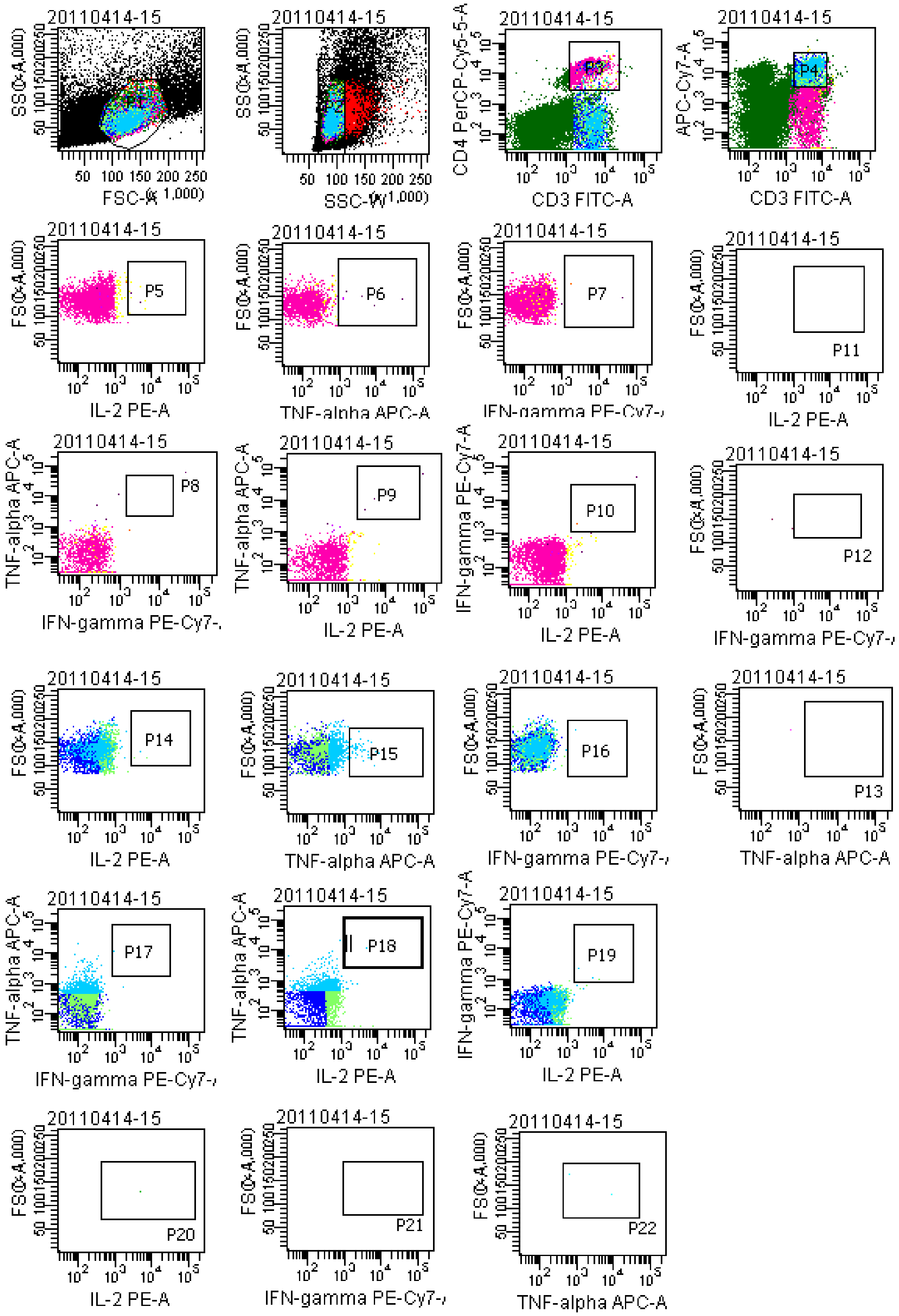

284W-control:00R0023 (peptide)

FACSDiva Version 6.0

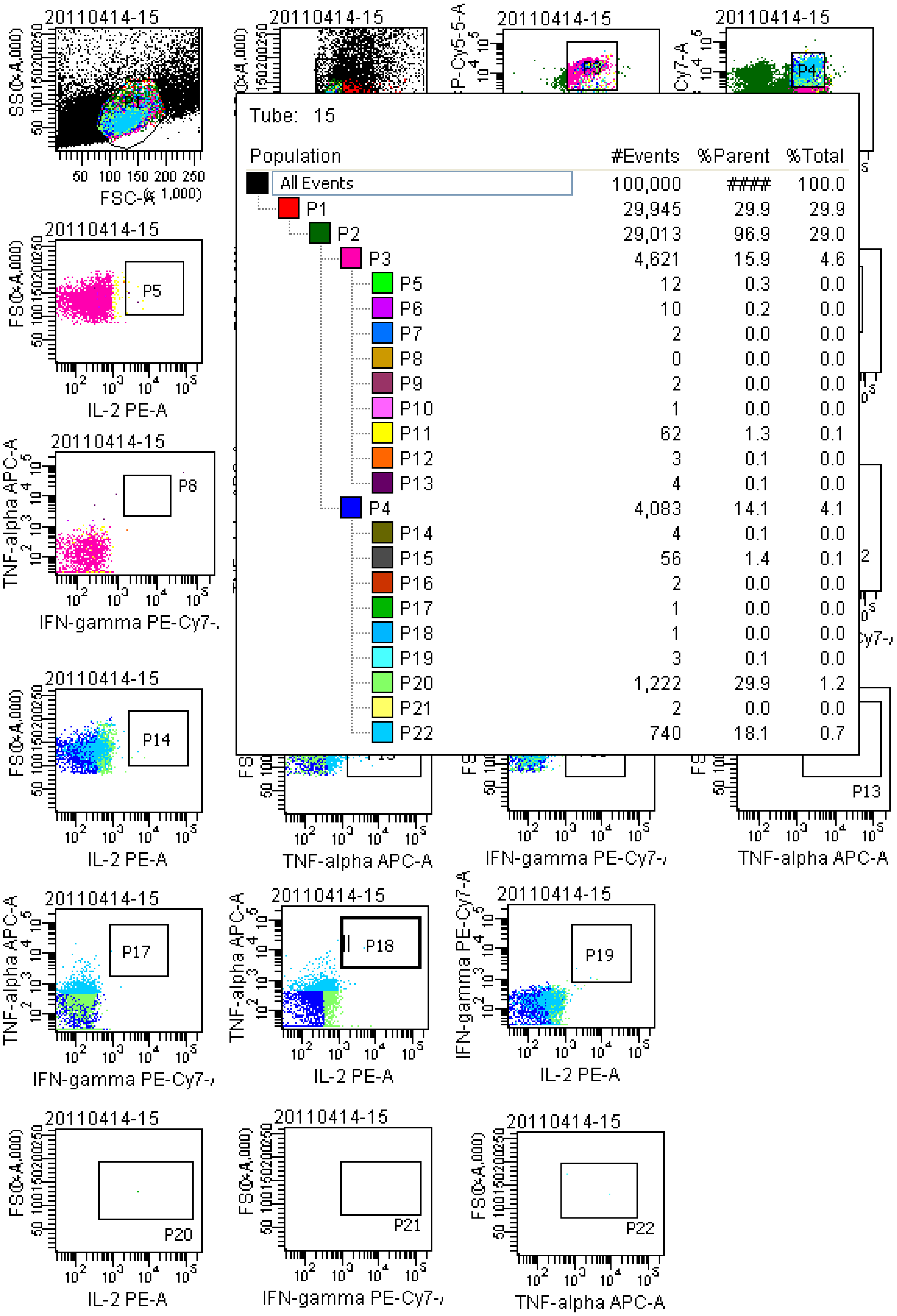

Tube: 15

Population	#Events	%Parent	%Total
All Events	100,000	####	100.0
P1	29,945	29.9	29.9
P2	29,013	96.9	29.0
P3	4,621	15.9	4.6
P5	12	0.3	0.0
P6	10	0.2	0.0
P7	2	0.0	0.0
P8	0	0.0	0.0
P9	2	0.0	0.0
P10	1	0.0	0.0
P11	62	1.3	0.1
P12	3	0.1	0.0
P13	4	0.1	0.0
P4	4,083	14.1	4.1
P14	4	0.1	0.0
P15	56	1.4	0.1
P16	2	0.0	0.0
P17	1	0.0	0.0
P18	1	0.0	0.0
P19	3	0.1	0.0
P20	1,222	29.9	1.2
P21	2	0.0	0.0
P22	740	18.1	0.7

284W-control:00R0023 (negative)

FACSDiva Version 6.0

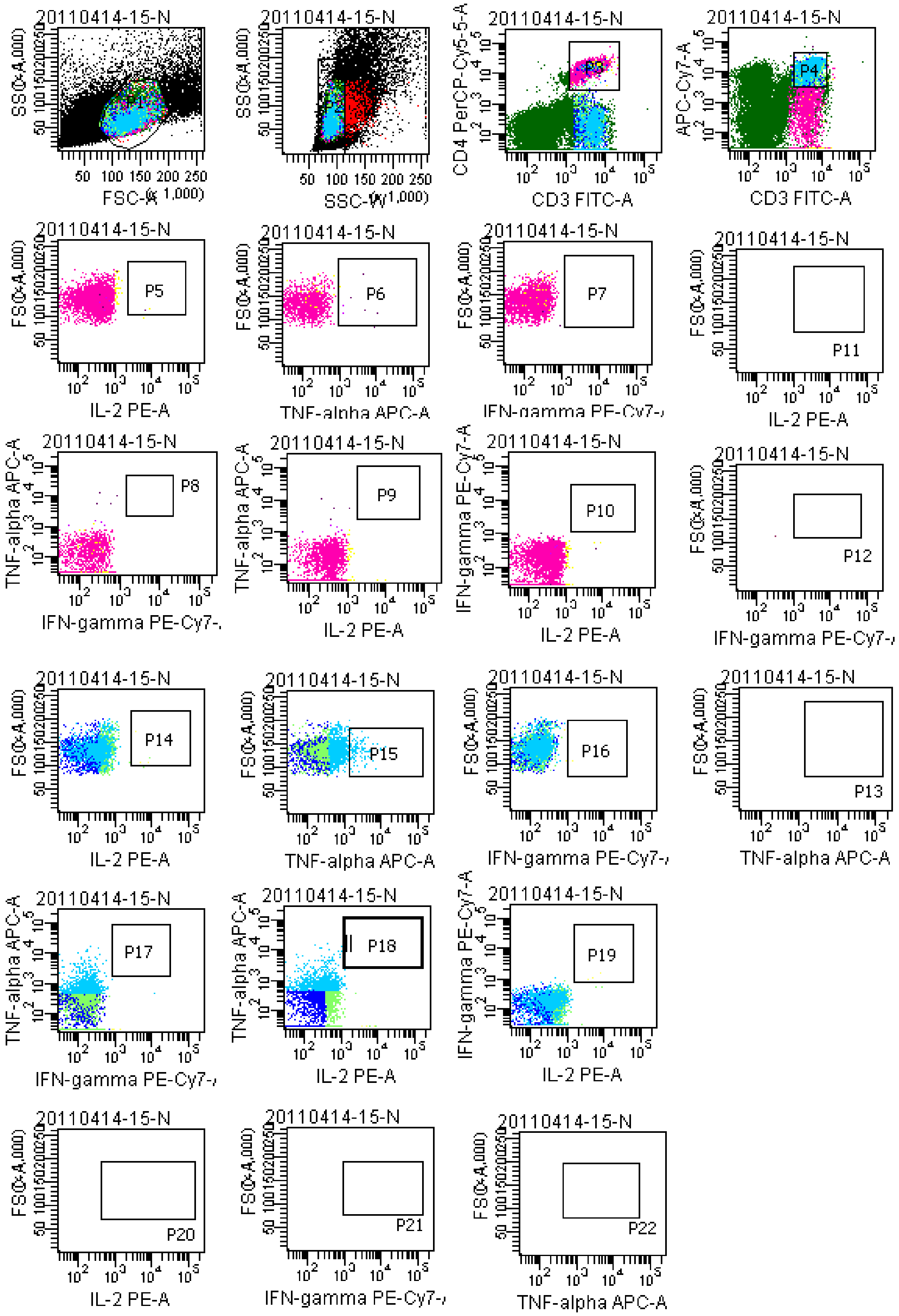

284W-control:00R0023 (negative)

FACSDiva Version 6.0

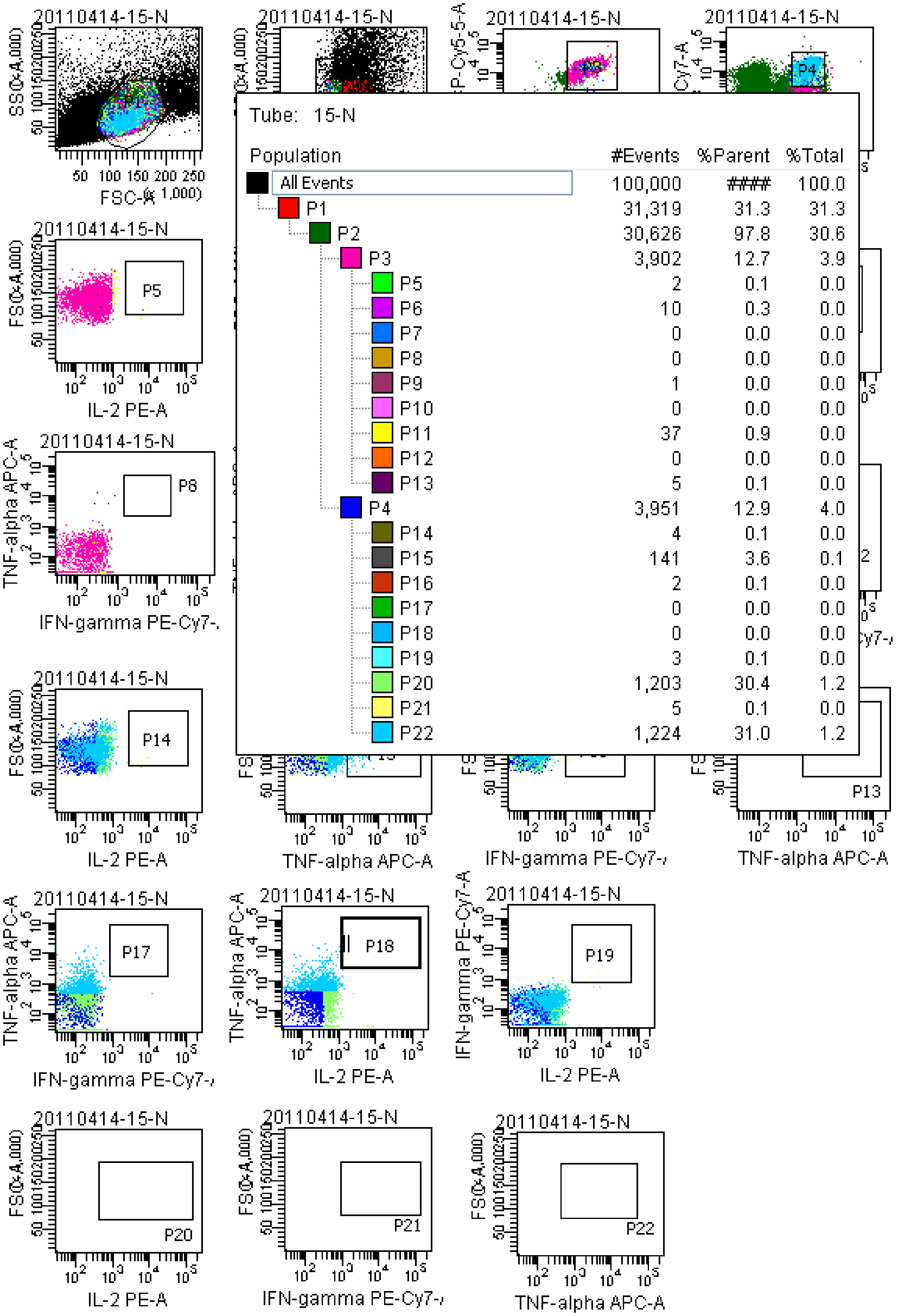

Tube: 15-N

Population	#Events	%Parent	%Total
All Events	100,000	####	100.0
P1	31,319	31.3	31.3
P2	30,626	97.8	30.6
P3	3,902	12.7	3.9
P5	2	0.1	0.0
P6	10	0.3	0.0
P7	0	0.0	0.0
P8	0	0.0	0.0
P9	1	0.0	0.0
P10	0	0.0	0.0
P11	37	0.9	0.0
P12	0	0.0	0.0
P13	5	0.1	0.0
P4	3,951	12.9	4.0
P14	4	0.1	0.0
P15	141	3.6	0.1
P16	2	0.1	0.0
P17	0	0.0	0.0
P18	0	0.0	0.0
P19	3	0.1	0.0
P20	1,203	30.4	1.2
P21	5	0.1	0.0
P22	1,224	31.0	1.2
